# Supplementary material for: Immune features are associated with response to neoadjuvant chemo-immunotherapy for muscle-invasive bladder cancer
Source: Nat Commun. 2024 May 24;15:4448. doi: 10.1038/s41467-024-48480-1 (PMC11126571; doi:10.1038/s41467-024-48480-1)
Supplement: Supplementary file 1 — Supplementary Information [file 41467_2024_48480_MOESM1_ESM.pdf]

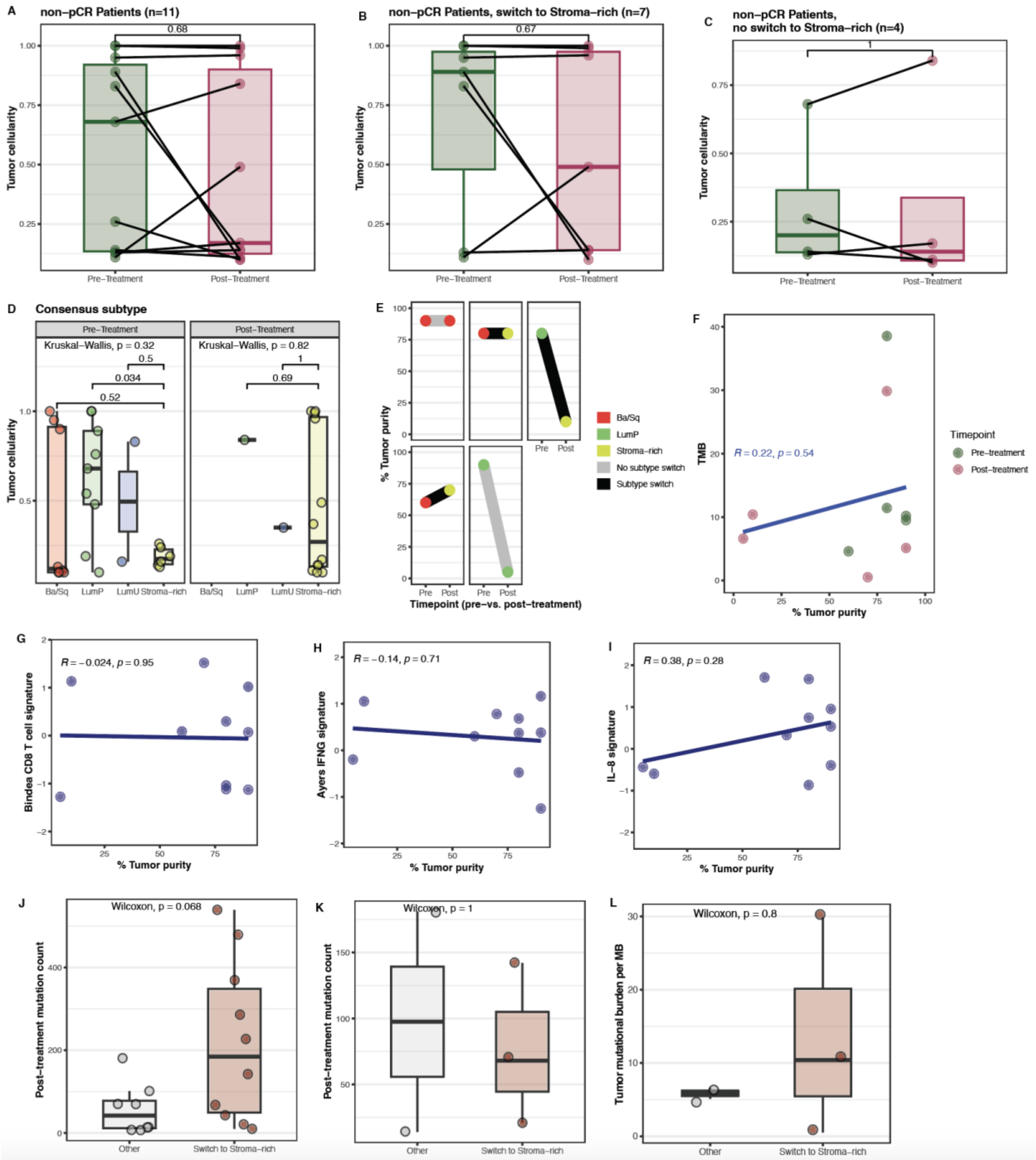

Supplementary Figure 1. Cellularity of pre-and post-treatment tumors. (A) Pre-vs. post-treatment tumor cellularity values (as calculated by Sequenza) in non-pCR tumors (n=11). (B) Pre-vs. post-treatment cellularity in non-pCR tumors that changed to Stroma-rich (n=11). (C) Pre-versus post-treatment cellularity values in non-pCR patients with tumors that did not change to Stroma-rich (n=11). (D) Pre-treatment (all patients, n=24) vs. post-treatment (non-pCR patients, n=14) cellularity by

Consensus subtype (Pre-Treatment: 8 Ba/Sq, 8 LumP, 2 LumU, 6 Stroma-rich; Post-Treatment: 0 Ba/Sq, 1 LumP, 1 LumU, 12 Stroma-rich). (E) Changes in tumor purity and Consensus subtype across treatment (n=5 patients; Pre-Treatment: 3 Ba/Sq, 2 LumP, 0 Stroma-rich; Post-Treatment: 1 Ba/Sq, 1 LumP, 3 Stroma-rich). (F) Percent tumor purity versus TMB in pre-and post-treatment tumors (n=5). Correlations of percent tumor purity in pre-and post-treatment samples with three immune gene signatures: Bindea_CD8_T_cells (G), Ayers_IFNG (H), and IL-8 signature (I; n=5). (J) Post-treatment mutation counts in pre-treatment tumors that did (n=10) or did not (n=8) switch to Stroma-rich subtype from pre-to post-treatment. (K) Post-treatment mutation counts in pre-treatment tumors with pathologic purity data that did (n=3) or did not (n=2) switch to Stroma-rich subtype from pre-to post-treatment. (L) Tumor mutational burden in pre-treatment tumors with pathologic purity data that did (n=3) or did not (n=2) switch to Stroma-rich subtype from pre-to post-treatment.

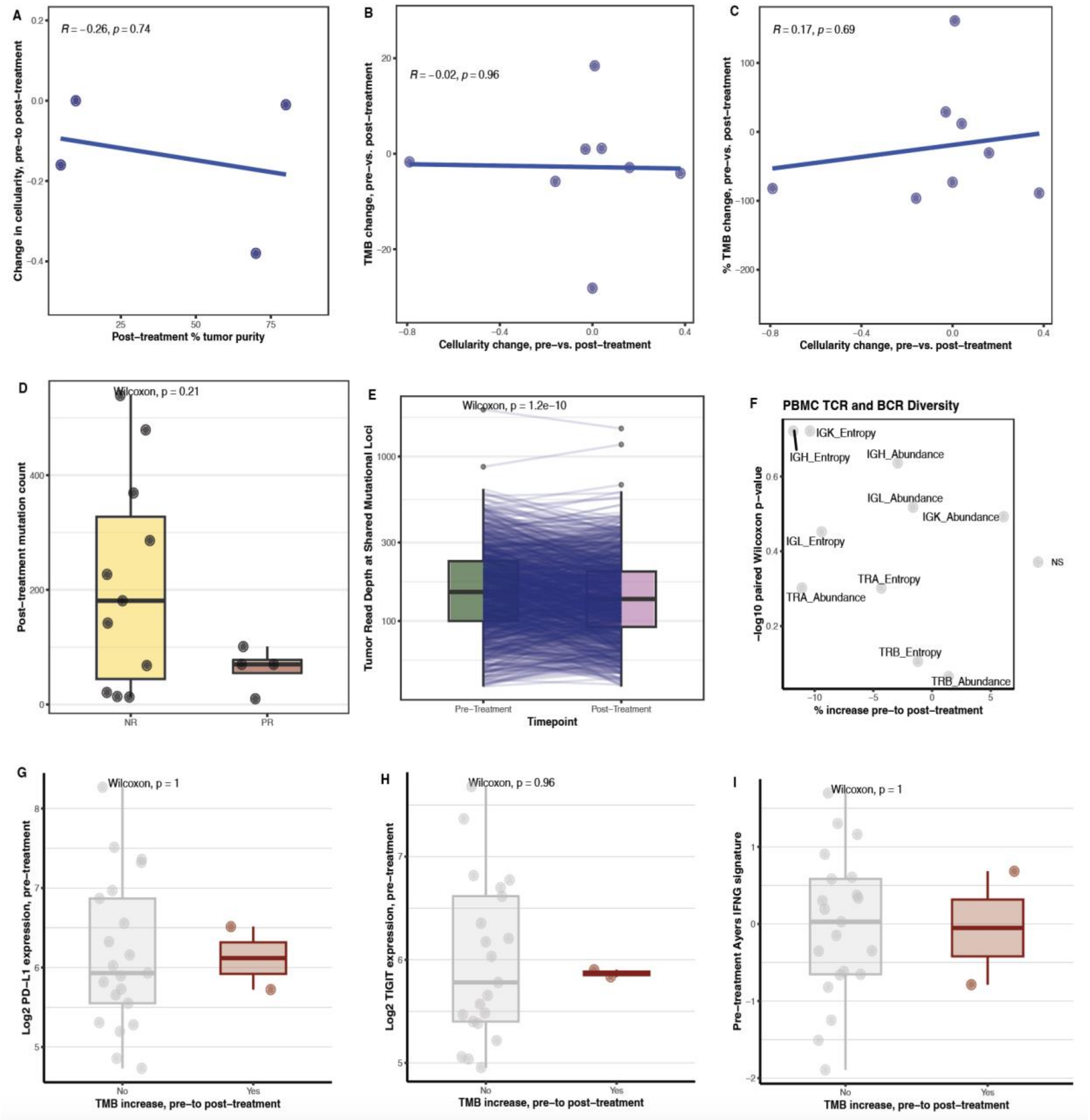

Supplementary Figure 2. Changes in molecular features during treatment. (A) Post-treatment percent tumor purity versus change in cellularity across treatment (n=4). (B) Change in TMB versus change in cellularity across treatment (n=8). (C) Percent change in TMB versus change in cellularity across treatment (n=8). (D) Post-treatment mutation counts from maftools compared in NR versus PR among tumors with paired TMB data (n=15). (E) Tumor read depth at mutational loci shared between tumors from pre-treatment to post-treatment. (F) Change in TCR and BCR diversity in PBMCs across treatment (n=26). PD-L1 expression (G), TIGIT expression (H), and Ayers_IFNG immune gene signature

(I) in the two patients with a large increase in TMB across treatment versus the other non-pCR patients (n=23).

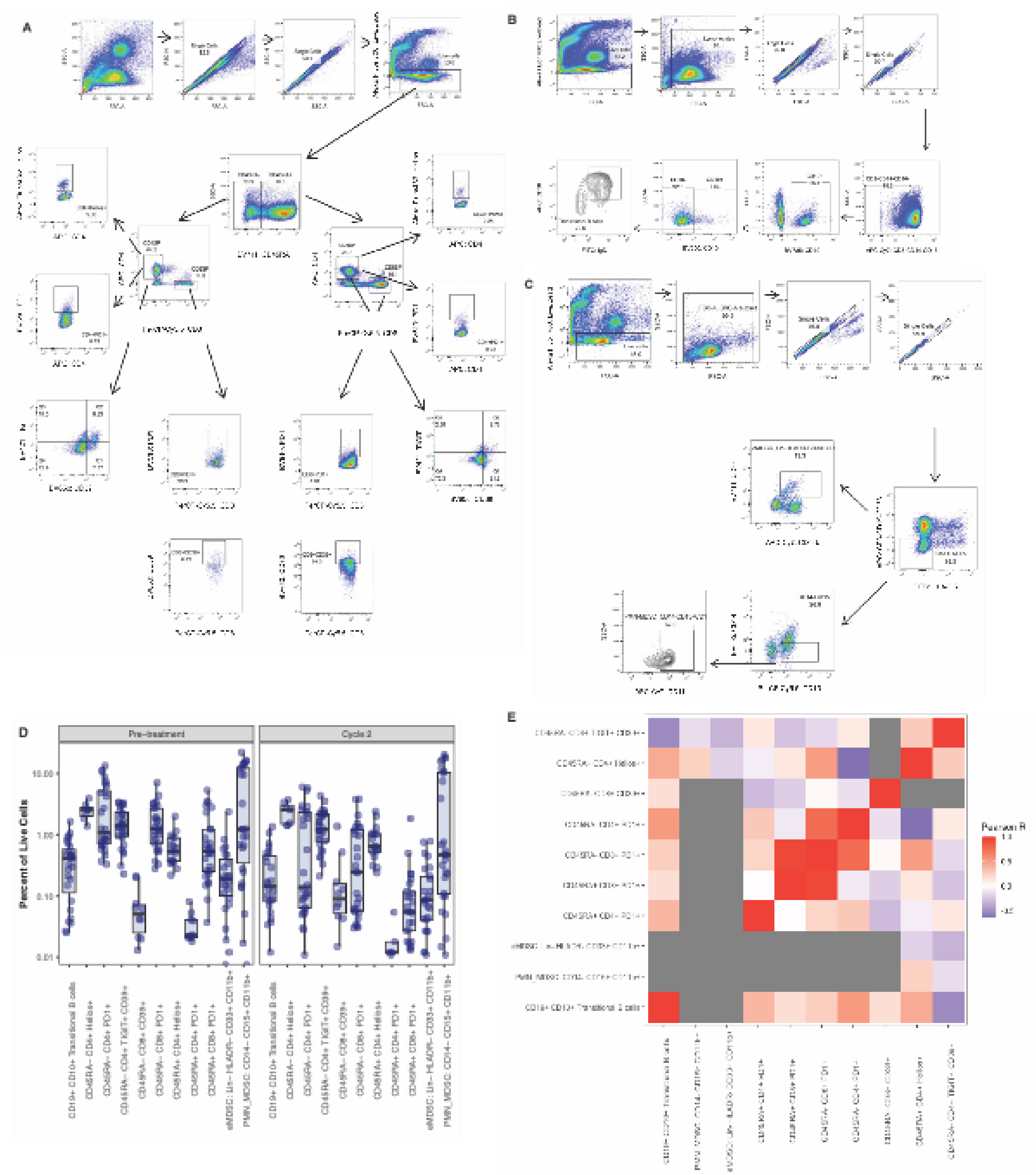

Supplementary Figure 3. Flow cytometry analysis. Example FACS plots for (A) T cells, (B) B cells, and (C) myeloid cells. (D) Quantification of all populations with significant changes from pre-to post-

treatment (n=29 patients). (E) Correlations of the 10 immune populations with significant changes from pre-to post-treatment (n=29 patients).

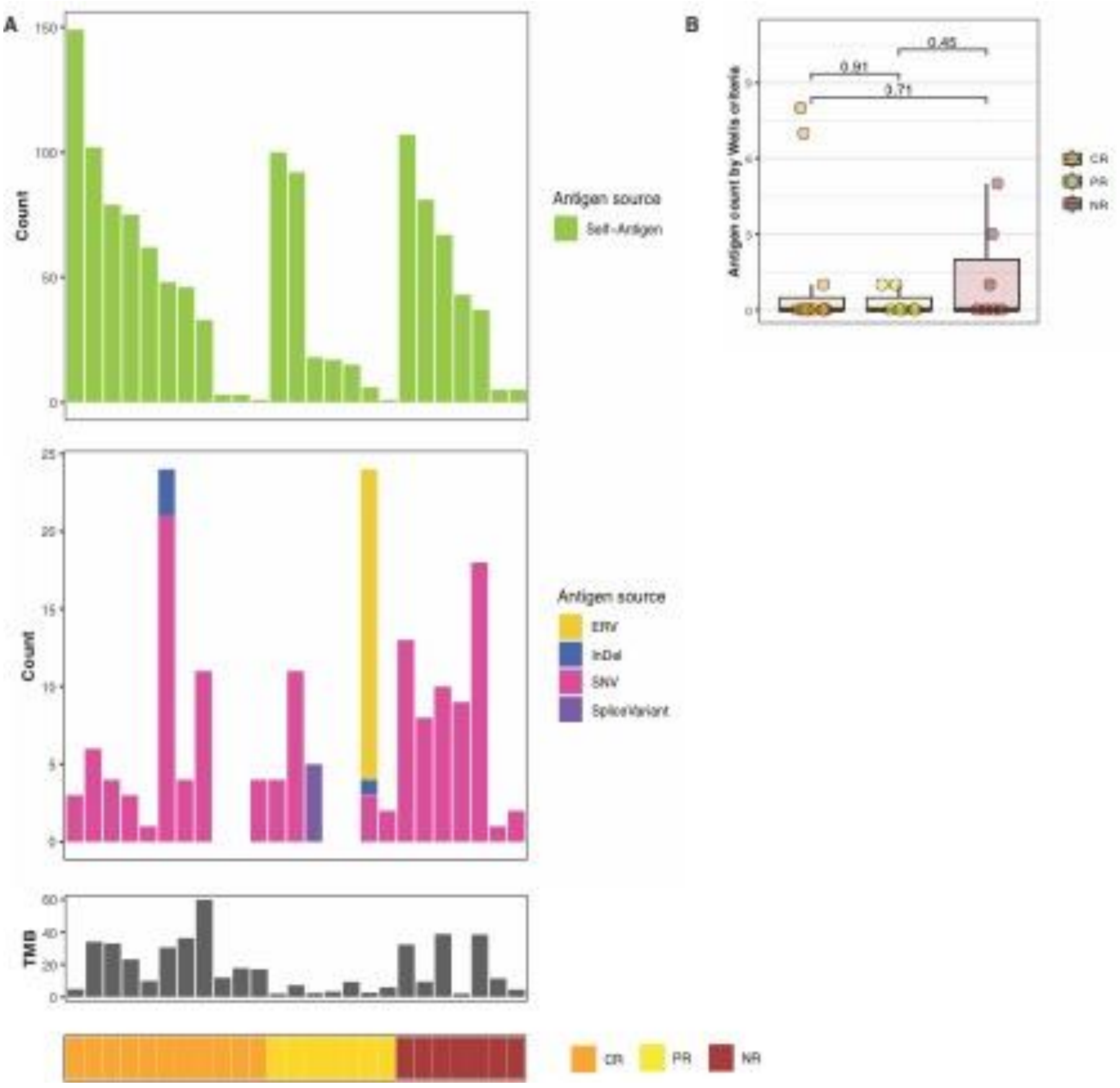

Supplementary Figure 4. Predicted antigens by clinical response group. (A) Predicted self-antigens, predicted neoantigens and TMB by clinical response group (n=25). (B) Predicted antigens by clinical response group with the more stringent TESLA antigen selection criteria (n=25: 11 CR, 7 PR, 7 NR).

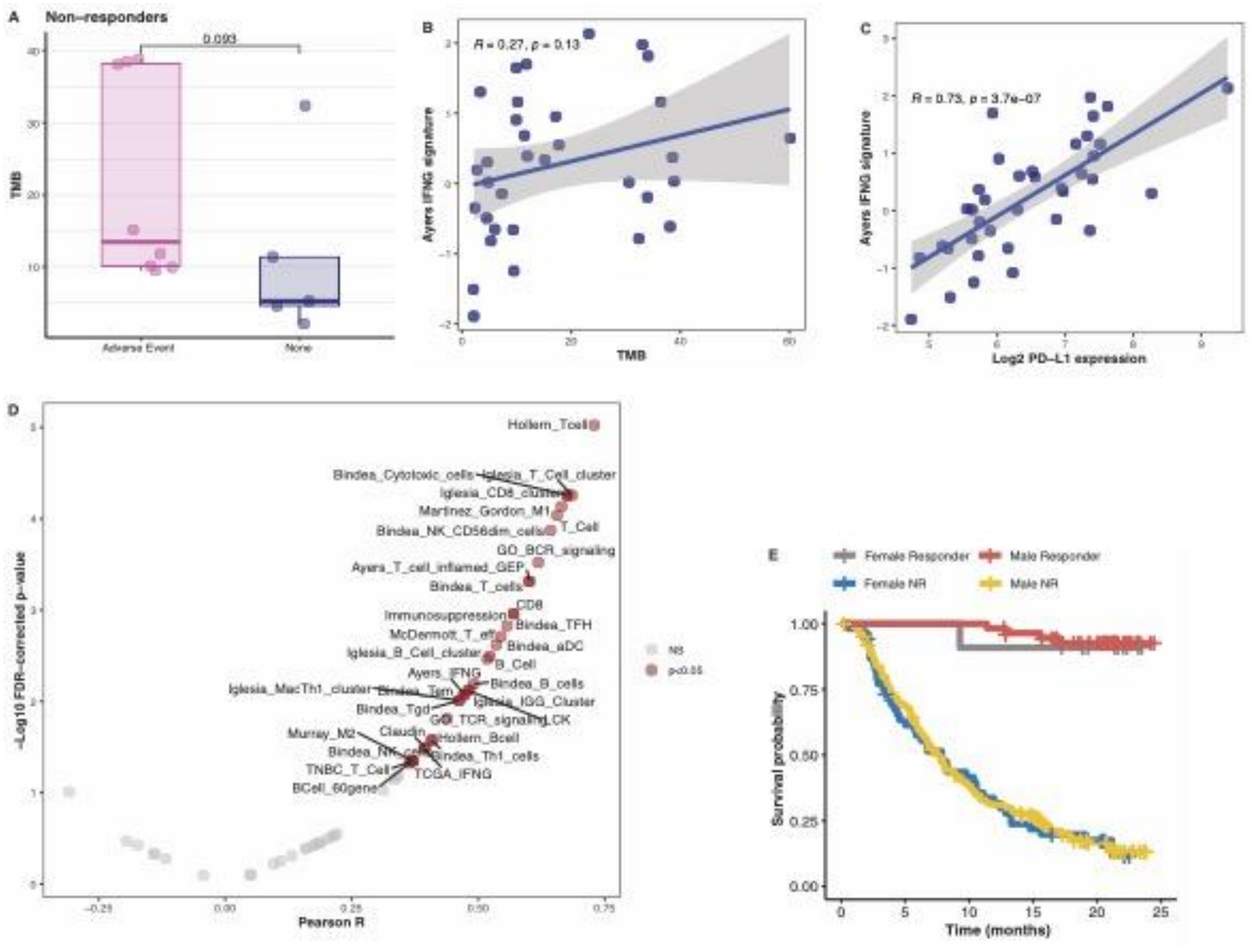

Supplementary Figure 5. Associations between potential predictive biomarkers. (A) Comparison of pre-treatment TMB in patients with (n=8) and without (n=5) adverse events. (B) Correlation of TMB with Ayers_IFNG signature at pre-treatment (n=33). (C) Correlation of PD-L1 expression and Ayers_IFNG signature at pre-treatment (n=37). (D) Correlation of Bindea_CD8_T_cell signature with the other immune gene signatures (n=37). (E) Kaplan-Meier survival curves of female and male patients by response status in IMvigor210 (n=298: Female Responder 11, Female NR 54, Male Responder 57, Male NR 176).

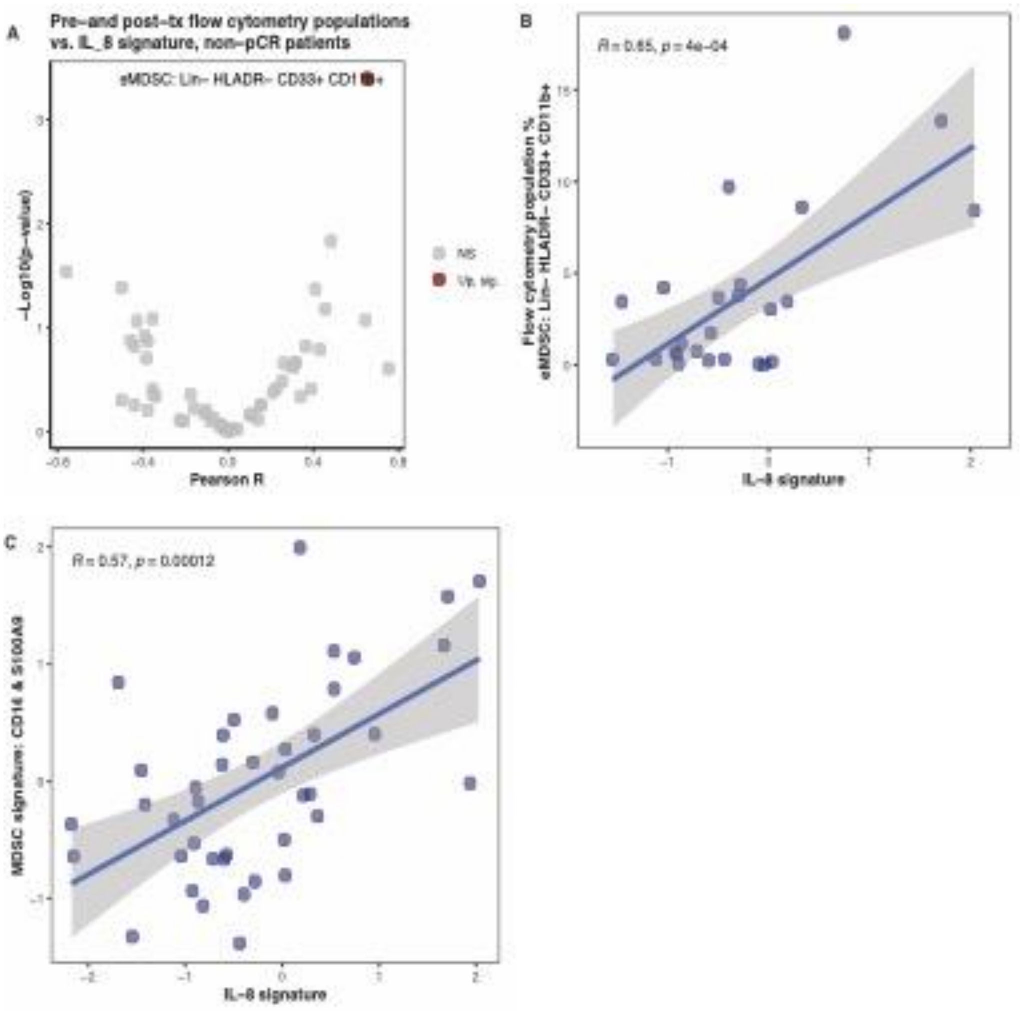

Supplementary Figure 6. Associations with IL-8 signature. (A) Correlations of peripheral blood flow cytometry population percentages with tumor IL-8 signature from pre-and post-treatment samples in non-pCR patients. (B) Correlation of peripheral blood eMDSC flow cytometry population percentage with tumor IL-8 signature from pre-and post-treatment samples in non-pCR patients. (C) Pearson correlation of MDSC signature[1] with the IL-8 signature.

Supplementary Table 1. T cell panel

Antibodies	Fluorophore	Catalog number	Clone	Dilution / volume per 100uL cells	Validation
Live/Dead (dye)	FVS 700	BD 564997	N/A	1:8000	link
CD45RA	BV711	BioLegend 304138	HI100	5uL	link
CD4	APC-H7	BD 560837	L200	5uL	link
CD8	PerCP-Cy5.5	BD 565310	SK1	5uL	link
CD25	PE	BD 557138	M-A251	20uL	link
CD127	PE-Cy7	BD 560822	HIL-7R-M21	5uL	link
TIGIT	BV421	BioLegend 372710	A15153 G	5uL	link
PD1	BV510	BD 563076	EH12.1	5uL	link
CD39	BV650	BD 563681	TU66	5uL	link
CTLA4	BV605	BioLegend 369610	BNI3	5uL	link
Foxp3	AlexaFluor 647	BD 560045	259D/C7	20uL	link

Supplementary Table 2. B cell panel

Antibodies	Fluorophore	Catalog number	Clone	Dilution / volume per 100uL cells	Validation
Live/Dead (dye)	FVS 700	BD 564997	N/A	1:8000	link
CD3	APC-Cy7	BioLegend 344818	SK7	5uL	link
CD14	APC-Cy7	BioLegend 325620	HCD14	5uL	link
CD16	APC-Cy7	BioLegend 302018	3G8	5uL	link
CD10	BV650	BD 563734	HI10a	5uL	link
CD19	BV785	BioLegend 302240	HIB19	5uL	link
CD38	APC	BioLegend 303510	HIT2	5uL	link
CD21	BV711	BD 563163	B-ly4	5uL	link
CD27	PE-CF594	BD 562297	M-T271	5uL	link
IL21R	BV421	BioLegend 347809	2G1-K12	5uL	link
IgD	FITC	BD 555778	IA6-2	20uL	link
Ki67	PerCP-Cy5.5	eBioscience 46-5699-42	20Raj1	5uL	link

Supplementary Table 3. Myeloid panel

Antibodies	Fluorophore	Catalog Number	Clone	Dilution / volume per 100uL cells	Validation
Live/Dead (dye)	FVS 700	BD 564997	N/A	1:8000	link
CD3	APC	BD555342	HIT3a	20uL	link
CD19	APC	BD555415	HIB19	20uL	link
CD56	APC	BD555518	B159	20uL	link
CD33	BV711	BD 563171	WM5 3	5uL	link
CD11b	APC-Cy7	BD 557754	ICRF 44	5uL	link
CD11c	PE-Cy7	BD 561356	B-ly6	5uL	link
CD14	BV605	BD 564054	M5E2	5uL	link
CD15	PerCP-Cy5.5	BD 560828	HI98	5uL	link
CD16	PE	BD 556619	3G8	20uL	link
CD86	BV421	BD 562433	2331	5uL	link
CD123	BV510	BD 563072	9F5	5uL	link
HLA-DR	BB515	BD 564516	G46-6	5uL	link

Supplementary Table 4. Immune gene signatures.

Signature	Description
Ayers_IFNG[2]	
Ayers_T_cell_inflamed_GEP[2]	
B_Cell[3]	
BCell_60gene[4]	
Bindea_aDC[5]	activated dendritic cell
Bindea_B_cells[5]	
Bindea_CD8_T_cells[5]	
Bindea_Cytotoxic_cells[5]	
Bindea_DC[5]	
Bindea_Eosinophils[5]	
Bindea_iDC[5]	inactivated dendritic cell
Bindea_Neutrophils[5]	
Bindea_NK_CD56bright_cells[5]	
Bindea_NK_CD56dim_cells[5]	
Bindea_NK_cells[5]	
Bindea_pDC[5]	plasmacytoid dendritic cell
Bindea_T_cells[5]	
Bindea_T_helper_cells[5]	
Bindea_Tcm[5]	central memory T cell
Bindea_Tem[5]	effector memory T cell
Bindea_TFH[5]	follicular helper T cell
Bindea_Tgd[5]	gamma-delta T cell
Bindea_Th1_cells[5]	
Bindea_Th2_cells[5]	
BIOCARTA_TGFB_Pathway[6]	
Byers_EMT[7]	epithelial-mesenchymal transition
CD8[3]	
Claudin[8]	CDLN3, CLDN4, CLDN7

EMT_DOWN[9]	
EMT_Stroma_core_18[10]	
EMT_Stroma_core_8[10]	
EMT_UP[10]	
FTBRS[11]	pan-fibroblast TGF-β response
GO_TCR_signaling[3]	T cell receptor signaling GO term
GO_BCR_signaling[3]	B cell receptor signaling GO term
Hollern_Bcell[12]	
Hollern_Tcell[12]	
Iglesia_B_Cell_cluster[3]	
Iglesia_CD68_cluster[3]	
Iglesia_CD8_cluster[3]	
Iglesia_IGG_Cluster[3]	
Iglesia_MacTh1_Cluster[3]	
Iglesia_T_Cell_Cluster[3]	
IL_8[3]	
Immunosuppression[9]	
LCK[3]	
Mac_CSF1[3]	
Macrophages[5]	
Martinez_Gordon_M1[13]	
Martinez_Gordon_M2[13]	
Mast_cells[5]	
McDermott_T_eff[14]	
Murray_M1[15]	
Murray_M2[15]	
T_Cell[3]	
TCGA_IFNG[16]	
TIC[17]	
TNBC_B_Cell[3]	
TNBC_T_Cell[3]	
Vincent_IPRES_NonResponder[18]	
Vincent_IPRES_Responder[18]	

Supplementary Table 5. Clinical feature associations with survival.

Feature	Hazard ratio	p-value
Received prior intravesical therapy	0.3	0.2
Former smoker	0.6	0.5
Current smoker	1.2	0.8
Years smoked	1.0	0.5
ECOG-PS = 1	0.3	0.2

Sample Size, Power Calculations, and Accrual

The primary endpoint of this study is rate of pathological downstaging, defined as the proportion of patients who achieve absence of muscle-invasive disease at the time of cystectomy (<pT2). Based on a prior retrospective study [14] and two prospective randomized clinical trials (MRC/EORTC trial and Intergroup 0080 trial) utilizing a conventional schedule of cisplatin-based neoadjuvant chemotherapy, the null pathological downstaging rate is 35%.[5, 16] A Simon's two-stage minimax design will be used for this study.[66] The null hypothesis that the true pathological downstaging rate is 35% will be tested against a one-sided alternative that it is higher. In the first stage, 21 patients will be accrued. If there are 8 or fewer patients with pathologic downstaging (<pT2) in these 21 patients, then the study will be stopped for futility, and the treatment regimen would be considered as uninteresting. Otherwise, 18 additional patients will be accrued for a total of 39. The null hypothesis will be rejected if 19 or more of the 39 patients have been downstaged to < pT2, and the treatment regimen will be considered worthy of future investigation. This design yields a type I error rate of 0.05 and power of 80% when the true response rate is 55%, representing a 20% improvement on the pathological downstaging rate previously reported.

Supplementary References

1. Zhao, F. *et al.* S100A9 a new marker for monocytic human myeloid-derived suppressor cells. *Immunology* **136**, 176–183 (2012).
2. Ayers, M. *et al.* IFN-γ–related mRNA profile predicts clinical response to PD-1 blockade. *J. Clin. Invest.* **127**, 2930–2940 (2017).
3. Iglesia, M. D. *et al.* Prognostic B-cell Signatures Using mRNA-Seq in Patients with Subtype-Specific Breast and Ovarian Cancer. *Clin. Cancer Res.* **20**, 3818–3829 (2014).
4. Schmidt, M. *et al.* A Comprehensive Analysis of Human Gene Expression Profiles Identifies Stromal Immunoglobulin κ C as a Compatible Prognostic Marker in Human Solid Tumors. *Clin. Cancer Res.* **18**, 2695–2703 (2012).
5. Bindea, G. *et al.* Spatiotemporal Dynamics of Intratumoral Immune Cells Reveal the Immune Landscape in Human Cancer. *Immunity* **39**, 782–795 (2013).
6. Shi, W. *et al.* Functional analysis of multiple genomic signatures demonstrates that classification algorithms choose phenotype-related genes. *Pharmacogenomics J.* **10**, 310–323 (2010).
7. Byers, L. A. *et al.* An Epithelial–Mesenchymal Transition Gene Signature Predicts Resistance to EGFR and PI3K Inhibitors and Identifies Axl as a Therapeutic Target for Overcoming EGFR Inhibitor Resistance. *Clin. Cancer Res.* **19**, 279–290 (2013).
8. Prat, A. *et al.* Phenotypic and molecular characterization of the claudin-low intrinsic subtype of breast cancer. *Breast Cancer Res.* **12**, R68 (2010).
9. Kardos, J. *et al.* Claudin-low bladder tumors are immune infiltrated and actively immune suppressed. *JCI Insight* **1**, (2016).
10. Wang, L. *et al.* EMT- and stroma-related gene expression and resistance to PD-1 blockade in urothelial cancer. *Nat. Commun.* **9**, 3503 (2018).

11. Mariathasan, S. *et al.* TGFβ attenuates tumour response to PD-L1 blockade by contributing to exclusion of T cells. *Nature* **554**, 544–548 (2018).
12. Hollern, D. P. *et al.* B Cells and T Follicular Helper Cells Mediate Response to Checkpoint Inhibitors in High Mutation Burden Mouse Models of Breast Cancer. *Cell* **179**, 1191-1206.e21 (2019).
13. Martinez, F. O. & Gordon, S. The M1 and M2 paradigm of macrophage activation: time for reassessment. *F1000Prime Rep.* **6**, (2014).
14. McDermott, D. F. *et al.* Clinical activity and molecular correlates of response to atezolizumab alone or in combination with bevacizumab versus sunitinib in renal cell carcinoma. *Nat. Med.* **24**, 749–757 (2018).
15. Murray, P. J. *et al.* Macrophage Activation and Polarization: Nomenclature and Experimental Guidelines. *Immunity* **41**, 14–20 (2014).
16. Thorsson, V. *et al.* The Immune Landscape of Cancer. *Immunity* **48**, 812-830.e14 (2018).
17. Chan, K. S. *et al.* Identification, molecular characterization, clinical prognosis, and therapeutic targeting of human bladder tumor-initiating cells. *Proc. Natl. Acad. Sci.* **106**, 14016–14021 (2009).
18. Zeidner, J. F. *et al.* Genomics Reveal Potential Biomarkers of Response to Pembrolizumab after High Dose Cytarabine in an Ongoing Phase II Trial in Relapsed/Refractory AML. *Blood* **132**, 4054–4054 (2018).

LCCC 1520: Phase II Single Arm Study of Gemcitabine and Cisplatin plus Pembrolizumab as Neoadjuvant Therapy Prior to Radical Cystectomy in Patients with Muscle-Invasive Bladder Cancer

Principal Investigator
Matthew I. Milowsky, MD
170 Manning Drive,
Campus Box 7305
Chapel Hill, NC 27599
919-843-7942 (phone); 919-966-6735 (fax)
matt_milowsky@med.unc.edu

Co-Principal Investigator
Tracy L. Rose, MD MPH
919-962-8561 (phone); 919-966-6735 (fax)
tracy_rose@med.unc.edu

Biostatistician
Allison Deal, MS
Lineberger Comprehensive Cancer Center
University of North Carolina at Chapel Hill
Chapel Hill, NC 27599-7295
Email: allison_deal@med.unc.edu

Clinical Protocol Office: Lineberger Comprehensive Cancer Center
The University of North Carolina at Chapel Hill
450 West Drive, 3rd Floor, CB# 7295; Chapel Hill, NC 27599-7295

UNC Multicenter Project Manager:
Phone: 919-966-7359
Fax: 919-966-4300

Sponsor: Lineberger Comprehensive Cancer Center
Funding Source: Merck, Inc.
Merck Reference # IND 122,753

Version: September 25, 2018

PROTOCOL AMENDMENT #11

AMENDMENT INCORPORATES:

[X] Editorial, administrative changes

[ ] Scientific changes (IRB approval)

[__] Therapy changes (IRB approval)

[__] Eligibility changes (IRB approval)

AMENDMENT RATIONALE AND SUMMARY:

- Minor editorial and formatting changes were made.
- T3 analysis was clarified to state "total or free T3" throughout the protocol.
- Dose modification for kidney dysfunction has been updated to state that both gemcitabine and cisplatin administration can proceed if creatinine clearance improves to ≥ 50ml/min within 1 week of scheduled day 1.
- The guidance for dose modifications for grade 3 and above, non-hematological toxicities was updated to note that treatment can also be resumed at the same dose level under the discretion of the treating physician.
- The number of ACD tubes have been updated to seven from three.
- Pembrolizumab language was updated per the new IB version 16.

THE ATTACHED VERSION DATED September 25, 2018 INCORPORATES THE ABOVE REVISIONS

ATTACH TO THE FRONT OF EVERY COPY OF THIS PROTOCOL

PROTOCOL AMENDMENT #10

AMENDMENT INCORPORATES:

[X] Editorial, administrative changes

[X] Scientific changes (IRB approval)

[__] Therapy changes (IRB approval)

[__] Eligibility changes (IRB approval)

AMENDMENT RATIONALE AND SUMMARY:

The LCCC1520 protocol is updated to clarify that sequential boundaries to suspend the trial will be used for subjects enrolled after the approval of Amendment 9. The protocol was updated in Amendments #8 and #9 to address a higher number than expected grade 3-4 acute kidney injury and myelosuppression adverse events. In these amendments, the lead-in dose of pembrolizumab was removed, administration of cisplatin was changed to split dosing rather than administration of one dose, a safety lab draw on cycle 1 day 15 was added and clarification in reference to day 8 dose modifications was made. These changes alter the risk profile of the study and thus the sequential boundaries for suspending the trial have been updated to be dependent on the enactment of these 2 amendments.

Additionally, the protocol was amended include updated language from Merck and incorporate minor editorial changes to maintain consistency throughout the protocol.

Editorial, administrative changes

- Section 4.2: added clarification of the dosing order to state that on Day 1 of each cycle pembrolizumab will be administered first, followed by gemcitabine and cisplatin. Each site must follow their institutional guidelines for administration of each drug and hydration.
- Section 4.4.3: updated to include correction that subjects should be placed back on study therapy within 2 weeks of the scheduled interruption, to maintain consistency throughout the protocol.
- Section 5.1: updated to include detailed pharmacy instructions on pembrolizumab.
- Section 7.2: updated to add a requirement that any grade 4 or 5 adverse events must be reported to the UNC Multicenter Project Manager immediately as they occur.

Scientific changes

- Section 4.4.3: updated Dose Modifications for Pembrolizumab for Events other than Infusion Reactions to reflect changes in the current Investigator's Brochure.
- Section 1.4.1: updated pembrolizumab clinical safety information to reflect changes in the current Investigator's Brochure.

- Section 8.3: updated to include the statement that sequential boundaries will be used to suspend the trial if unacceptable toxicity is seen in subjects enrolled after the approval of Amendment 9 of this protocol.
- Several portions of the protocol were updated to reflect that Merck no longer considers immune related adverse events, other than transaminitis, and pregnancy to be events of clinical interest.

THE ATTACHED VERSION DATED June 12, 2018 INCORPORATES THE ABOVE REVISIONS

ATTACH TO THE FRONT OF EVERY COPY OF THIS PROTOCOL

PROTOCOL AMENDMENT #9

AMENDMENT INCORPORATES:
[_x_] Editorial, administrative changes
[__] Scientific changes (IRB approval)
[_x_] Therapy changes (IRB approval)
[__] Eligibility changes (IRB approval)
[_x_] Other

AMENDMENT RATIONALE AND SUMMARY:

The LCCC1520 protocol is updated to add a safety lab draw on cycle 1 day 15 and to clarify exact protocol instructions for calculation of GFR, day 8 dose modifications, and timing of study imaging requirements if patients come off treatment early.

This amendment clarifies regulatory requirements pertaining to data management and monitoring, and FDA expedited reporting requirements per recent guidelines and policies.

The protocol is also updated in section 9.5.2 in accordance with Lineberger Comprehensive Cancer Center's policy on single subject exceptions to indicate that eligibility single subject exceptions are not permitted for this study and other types of single subject exceptions require proper regulatory review.

- Clarifications were made throughout the protocol that all use of a time "window" means +/- the duration of said time window.
- Section 3.1.10: The full CKD Epi equation was added to clarify use of non-BSA-adjusted equation.
- Section 4.2: Table changed to include that the duration of gemcitabine and cisplatin may be altered per institutional protocol.
- Section 4.4.1: Day 8 dose modifications were made adjusted to hold day 8 treatment for ANC <1000 (instead of 500) and platelets <75,000 (instead of 50,000) in order to decrease risk of severe neutropenia and febrile neutropenia.
- Section 4.4.2: Clarifications were made in multiple places to the dose modification table, particularly to include wording in multiple places that day 8 dosing should not be made up if held.
- Section 4.9: Details study withdrawal procedures and proper documentation were revised.
- Section 5.1.6: Updated to describe additional adverse events like Stevens-Johnson Syndrome, toxic epidermal necrolysis, myocarditis, sarcoidosis, and encephalitis reported by Merck associated to pembrolizumab.
- Section 6.1: The time and events table was updated to add Cycle 1 Day 15 lab draw with a +/- 1 day window as an additional safety lab for monitoring subjects and added windows to the long-term follow-up plan. Also the time and events table now notes that the imaging scans for pre-surgery should be 30 days with a +/- 14 day window after last dose of

pembrolizumab and not required to be repeated if come off treatment early and have had scans within 30 days.

- Clarified need to use CKD Epi throughout study in Section 6.3.1 and Section 6.3.2.
- Section 6.3.3: Added to describe the labs done on Day 15 of Cycle 1.
- Section 7.3.3 Updated IRB submission language to be consistent with the UNC IRB's standard operating procedures. Revised to remove language that states that UNC will submit an aggregate list of SAEs.
- Section 7.3.4: Updated to describe the FDA expedited reporting requirements.
- Section 9.2: Included financial disclosures as required documentation before site initiation.
- Section 9.4: Amended to detail data monitoring and management of the study.
- Section 9.5.2: Altered to summarize that the eligibility single subject exceptions are not permitted in this clinical trial.

THE ATTACHED VERSION DATED January 18, 2018 INCORPORATES THE ABOVE REVISIONS

ATTACH TO THE FRONT OF EVERY COPY OF THIS PROTOCOL

PROTOCOL AMENDMENT #8

AMENDMENT INCORPORATES:

_X__Editorial, administrative changes
___ Scientific changes (IRB approval)
_X_ Therapy changes (IRB approval)
___ Eligibility changes (IRB approval)
__ Other

AMENDMENT RATIONALE AND SUMMARY:

The LCCC1520 protocol is updated to remove the lead-in dose of pembrolizumab and to administer the cisplatin in a split dose rather than one dose. These changes were made to address a higher number than expected of grade 3-4 acute kidney injury and myelosuppression adverse events which were noted in the first cohort of enrolled patients.

- Section 1.0 Background and rationale: updated to include new information related to the rationale for the study as it has changed.
- Section 2.1 The primary and secondary objectives has changed with the elimination of reference to the lead in dose of pembrolizumab
- Eligibility Criteria: Item 3.1.15 has been changed to add whole blood collection on cycle 2, day 1
- Section 4.1 The schema was updated to reflect the deletion of the pembrolizumab lead-in dose and split dose cisplatin.
- Section 4.2 This section was updated with the new treatment plan.
- Section 6.3 the description of the lead-in dose related procedures was removed.
- Section 6.7.2 the 3 ACD tubes will now be collected pre-study, at Cycle 2 day 1, and at the time of cystectomy.

THE ATTACHED VERSION DATED May 9, 2017 INCORPORATES THE ABOVE REVISIONS

ATTACH TO THE FRONT OF EVERY COPY OF THIS PROTOCOL

PROTOCOL AMENDMENT #7

AMENDMENT INCORPORATES:
_X__Editorial, administrative changes
_X_ Scientific changes (IRB approval)
__ Therapy changes (IRB approval)
__ Eligibility changes (IRB approval)
__ Other

AMENDMENT RATIONALE AND SUMMARY:

The LCCC1520 protocol is updated to include the latest safety language required for Pembrolizumab IIT Study Protocols and includes revisions to management guidelines for infusion reactions (Section 4.2.3) dose modification requirements for adverse events (Section 4.4.3 and 4.4.4), changes in procedures required for reporting events of clinical interests and revisions (Sections 7.1.3, 7.3.2 and 7.3.3) to eligibility criteria (3.1.11 and 3.1.12).

THE ATTACHED VERSION DATED DECEMBER 7, 2016 INCORPORATES THE ABOVE REVISIONS

ATTACH TO THE FRONT OF EVERY COPY OF THIS PROTOCOL

PROTOCOL AMENDMENT #6

AMENDMENT INCORPORATES:
___Editorial, administrative changes
__ Scientific changes (IRB approval)
_X Therapy changes (IRB approval)
__ Eligibility changes (IRB approval)
__ Other

AMENDMENT RATIONALE AND SUMMARY:

4.4 Toxicities and Dosing Delays/Dose Modifications: Text was added to this section to note that once an individual is put on a split dose of cisplatin, they will remain on a split-dose.

4.4.2 Dose Modifications for GC for Non-Hematologic Toxicity: Text was added to the table to note that once an individual is put on a split-dose of cisplatin, they will remain on a split-dose for all subsequent cycles.

THE ATTACHED VERSION DATED September 13, 2016 INCORPORATES THE ABOVE REVISIONS

ATTACH TO THE FRONT OF EVERY COPY OF THIS PROTOCOL

PROTOCOL AMENDMENT #5

AMENDMENT INCORPORATES:

X Editorial, administrative changes
__ Scientific changes (IRB approval)
__ Therapy changes (IRB approval)
X Eligibility changes (IRB approval)
X Other

AMENDMENT RATIONALE AND SUMMARY:

3.1.10 Inclusion Criteria: in the table of laboratory values, renal function should be measured either by serum creatinine or by estimated GFR per CKD-EPI equation

3.2.9 Exclusion Criteria: the exclusion criterion regarding pneumonitis was changed to include a history of (non-infectious) pneumonitis.

4.2 Language was added noting that the Day 8 visit has a window of 1 day, as opposed to the other visits that have a 3 day window.

4.2.2 “PO” was added as an alternative to IV dexamethasone treatment.

6.1 Time and Events Table: “x”s were removed from the “tumor samples” item that were inadvertently placed in the table.

10.0 References: A new reference was added regarding the CKD-EPI equation

THE ATTACHED VERSION DATED July 7, 2016 INCORPORATES THE ABOVE REVISIONS

ATTACH TO THE FRONT OF EVERY COPY OF THIS PROTOCOL

PROTOCOL AMENDMENT #4

AMENDMENT INCORPORATES:

X Editorial, administrative changes
__ Scientific changes (IRB approval)
__ Therapy changes (IRB approval)
__ Eligibility changes (IRB approval)
X Other

AMENDMENT RATIONALE AND SUMMARY:

3.1.9 Inclusion Criteria: The wording was changed to state that patients “must agree to submission” of tumor tissue rather than stating that the patient must have sufficient tumor tissue.

4.1 Schema: The schema has been updated to reflect that patients must agree to submission of tissue for correlative studies, but adequate tissue is not required to enroll.

6.1 Time and Events Table: “cystectomy” was added as one of the study-related events

6.1 Time and events Table: the 10-day period for laboratory assessments was amendment to include “and other assessments”.

6.1 Time and Events Table: superscript “2” was added to the pembrolizumab lead-in visit to give a 3 day window to that visit.

7.3 and throughout the protocol: Language related to a separate guidance document for Events of Clinical Interest was removed since the Guidance Document is no longer being updated by Merck.

THE ATTACHED VERSION DATED May 25, 2016 INCORPORATES THE ABOVE REVISIONS

ATTACH TO THE FRONT OF EVERY COPY OF THIS PROTOCOL

PROTOCOL AMENDMENT #3

AMENDMENT INCORPORATES:

X Editorial, administrative changes
__ Scientific changes (IRB approval)
__ Therapy changes (IRB approval)
__ Eligibility changes (IRB approval)
X Other

AMENDMENT RATIONALE AND SUMMARY:

Changes in this amendment were largely clarifying ones. Specific changes are:

3.1.13 A description of adequate contraception was added.

Schema: the schema was updated with the removal of "green top" for what ACD tubes would be used. This change was also made throughout the protocol.

4.3 The timing of the cystectomy was changed from within 70 days to within 4-8 weeks if possible. This was also changed in other relevant parts of the protocol.

4.4 Information on treatment in the circumstance of dose delay was clarified.

6.2 Pre-study laboratory assessments were updated to match the Time and Events Table.

6.4 Laboratory assessments for the pre-surgical visit were updated to match the Time and Events Table.

11.2 The PRO-CTCAE Questionnaire was added to the protocol.

THE ATTACHED VERSION DATED March 29, 2016 INCORPORATES THE ABOVE REVISIONS

ATTACH TO THE FRONT OF EVERY COPY OF THIS PROTOCOL

PROTOCOL AMENDMENT #2

AMENDMENT INCORPORATES:

X Editorial, administrative changes
__ Scientific changes (IRB approval)
__ Therapy changes (IRB approval)
__ Eligibility changes (IRB approval)
_X Other

AMENDMENT RATIONALE AND SUMMARY:

Section 1.8: Language was added to explain the biospecimen handling procedures and the storage process, including storage for unspecified future research.

THE ATTACHED VERSION DATED February 9, 2016 INCORPORATES THE ABOVE REVISIONS

ATTACH TO THE FRONT OF EVERY COPY OF THIS PROTOCOL

PROTOCOL AMENDMENT #1

AMENDMENT INCORPORATES:
X Editorial, administrative changes
X Scientific changes (IRB approval)
X Therapy changes (IRB approval)
X Eligibility changes (IRB approval)
__Other

AMENDMENT RATIONALE AND SUMMARY:

1.4.1 Updated clinical information was added to the section on "Pembrolizumab Clinical Safety".

1.5 Pembrolizumab will be administered as a single dose alone at the start of the study

1.6 The use of the PRO-CTCAE and PROMIS was added.

1.7.2 The Claudin-low molecular subtype has been added to the planned biomarker studies.

2.2.4 A new secondary objective has been added that the study will estimate the proportion of patients who are alive at 3 years

3.1.9 The inclusion criterion regarding archived tissue was expanded and clarified.

3.2.15 An exclusion criterion was added that prohibits prior radiation therapy to the bladder for the purpose of treating urothelial carcinoma

4.1 and 4.2 The dosing of the monotherapy with pembrolizumab was clarified

5.1.6 Details from study results of other disease groups were added for the immune related adverse events.

5.1.8 Information on the use of contraception was added.

5.1.9 Information on fetal toxicity was added

5.1.11 toxicity related to presence in breast milk was added.

9.3 Affiliate site registration procedures were corrected.

THE ATTACHED VERSION DATED January 20, 2016 INCORPORATES THE ABOVE REVISIONS

ATTACH TO THE FRONT OF EVERY COPY OF THIS PROTOCOL

LCCC 1520: Phase II Single Arm Study of Gemcitabine and Cisplatin plus Pembrolizumab as Neoadjuvant Therapy Prior to Radical Cystectomy in Patients with Muscle-Invasive Bladder Cancer

Principal Investigator
Matthew I. Milowsky, MD
170 Manning Drive, 3rd Floor Physician's Office Building
Campus Box 7305
Chapel Hill, NC 27599
919-843-7942 (Phone)
919-966-6735 (Fax)
matt_milowsky@med.unc.edu

Signature Page

The signature below constitutes the approval of this protocol and the attachments and provides the necessary assurances that this trial will be conducted according to all stipulations of the protocol, including all statements regarding confidentiality, and according to local legal and regulatory requirements and applicable U.S. federal regulations and ICH guidelines.

Principal Investigator (PI) Name: Matthew Milowsky, MD

PI Signature: ___________________

Date: ____________________

Version date: September 25, 2018

Version: Amendment 11

TABLE OF CONTENTS

1.0 BACKGROUND AND RATIONALE 4

1.1 Study Synopsis..4

1.2 Bladder Cancer and Chemotherapy 4

1.3 The PD-L1/PD-1 Pathway in Cancer..7

1.4 Pembrolizumab 8

1.5 Rationale for Inclusion of Pembrolizumab as NAC for MIBC 11

1.6 Inclusion of Patient-Reported Outcomes version of the Common Terminology Criteria for Adverse Events (PRO-CTCAE) 12

1.7 Correlative Studies..12

2.0 STUDY OBJECTIVES..14

2.1 Primary Objective 14

2.2 Secondary Objectives..14

2.3 Exploratory Objectives 15

2.4 Endpoints 15

3.0 PATIENT ELIGIBILITY 16

3.1 Inclusion Criteria 16

3.2 Exclusion Criteria 18

4.0 TREATMENT PLAN..20

4.1 Schema..20

4.2 Treatment Dosage and Administration 21

4.3 Timing of Cystectomy after Chemotherapy 23

4.4 Toxicities and Dosing Delays/Dose Modifications 24

4.5 Concomitant Medications/Vaccinations (allowed & prohibited) 33

4.6 Duration of Therapy....35

4.7 Duration of Follow Up....35

4.8 Removal of Patients from Protocol Therapy....36

4.9 Study Withdrawal....36

4.10 Clinical Criteria for Early Trial Termination....36

5.0 DRUG INFORMATION....38

5.1 Pembrolizumab....38

5.2 Gemcitabine Hydrochloride....47

5.3 Cisplatin (CDDP)....49

6.0 EVALUATIONS AND ASSESSMENTS....52

6.1 Time and Events Table....52

6.2 Pre-Study Assessments....57

6.3 On-Study Treatment Assessments....57

6.4 Pre-Surgical Visit (post-pembrolizumab)....58

6.5 Cystectomy....59

6.6 Long Term Follow-Up Assessments....60

6.7 Correlative Studies Procedures....60

6.8 Assessment of Safety....62

6.9 Assessment of Efficacy....62

7.0 ADVERSE EVENTS....64

7.1 Definitions....64

7.2 Documentation of non-serious AEs or SARs....66

7.3 SAEs or Serious SARs and Events of Clinical Interest....66

7.4 Data and Safety Monitoring Plan....72

8.0 STATISTICAL CONSIDERATIONS 73

8.1 Study Design/Study Endpoints 73

8.2 Sample Size and Accrual 73

8.3 Continuous Monitoring for Toxicity 73

8.4 Data Analysis Plans 74

9.0 STUDY MANAGEMENT 75

9.1 Institutional Review Board (IRB) Approval and Consent 75

9.2 Required Documentation 75

9.3 Registration Procedures 75

9.4 Data Management and Monitoring/Auditing 76

9.5 Adherence to the Protocol 76

9.6 Amendments to the Protocol 78

9.7 Record Retention 79

9.8 Compliance with Trial Registration and Results Posting Requirements 79

9.9 Obligations of Investigators 79

10.0 REFERENCES 81

11.0 APPENDICES 85

11.1 Appendix A: ECOG Performance Status 85

11.2 Appendix B: Patient-Reported Symptom Assessment 86

11.3 Appendix C: The CKD-EPI Creatinine Equation 91

1.0 BACKGROUND AND RATIONALE

1.1 Study Synopsis

This multicenter single-arm phase II trial will evaluate the humanized monoclonal antibody targeted against cell surface receptor programmed cell death-1 (PD-1), pembrolizumab in combination with gemcitabine and cisplatin in patients with muscle-invasive bladder cancer (MIBC) as neoadjuvant therapy prior to radical cystectomy. The study will enroll 39 patients, using a Simon's two-stage design, with a primary endpoint of pathological downstaging to <pT2. Secondary objectives include toxicity assessment, rate of complete pathologic response (pT0), event free survival (EFS), and overall survival (OS).

Exploratory objectives include an evaluation of the predictive capacity of PD-L (ligand) 1 expression, immune gene signatures, and RNA subtypes of bladder cancer for pathological downstaging, and a characterization of the change in phenotype in tumor-infiltrating lymphocytes (TILs) before and after neoadjuvant therapy. We hypothesize that neoadjuvant treatment with pembrolizumab in combination with gemcitabine and cisplatin in MIBC will result in an improvement in the rate of pathological downstaging to <pT2 compared to historical controls. We hypothesize that each of the following (a) tumors of the basal and claudin-low subtypes, (b) increased PD-L1 mRNA expression, (c) effector immune infiltrate, (d) clonality in TILs and (e) decreased regulatory cell populations will be associated with a higher rate of pathological downstaging.

1.2 Bladder Cancer and Chemotherapy

Bladder cancer is the 6th most common cancer in the United States, with an estimated 74,690 new cases and 15,580 deaths for 2014.[1] Although most cases are non-invasive at initial diagnosis, nearly 25% of these cases will progress to MIBC. Further, 20-30% of patients have MIBC at the time of diagnosis.[2] Current guidelines for MIBC recommend neoadjuvant cisplatin-based chemotherapy followed by cystectomy based on randomized clinical trials and meta-analyses.[3-5] Despite this aggressive management, more than 50% of patients develop recurrent/metastatic disease within two years and ultimately die of complications related to bladder cancer.[6] The development of more effective treatment for MIBC is clearly warranted.

1.2.1 Neoadjuvant Chemotherapy as Standard of Care in MIBC

The mainstay of neoadjuvant chemotherapy (NAC) in MIBC has been cytotoxic cisplatin-based combination chemotherapy. The MVAC regimen (methotrexate, vinblastine, doxorubicin, and cisplatin) was introduced in the 1980's and was found to have more activity than single drug regimens for advanced bladder cancer.[7, 8] A randomized clinical trial later demonstrated an improvement in rate of pT0 and an improvement in OS after MVAC plus cystectomy compared to cystectomy alone (OS 77 vs 46 months, respectively, p=0.06) in patients with

MIBC.[3] Another randomized trial showed similar results with neoadjuvant CMV (cisplatin, methotrexate, vinblastine) plus cystectomy conferring a 5.5% improvement in OS at 3 years compared to cystectomy alone (p=0.075).[4] A meta-analysis published in 2005 demonstrated a significant survival benefit of 5% at 5 years with platinum-based combination NAC compared to cystectomy alone and NAC was thus adopted as standard of care.[5]

MVAC has been associated with substantial toxicity, most notably hematologic and gastrointestinal complications.[3] The combination of gemcitabine and cisplatin (GC) was compared with MVAC in a phase III randomized trial of subjects with metastatic urothelial carcinoma of the bladder. GC was found to have similar efficacy to MVAC (OS 14 vs 15.2 months, respectively, p=0.66) but with substantially less toxicity (febrile neutropenia 1% in GC group and 12% in MVAC group).[9] This study used a 28-day cycle of GC with cisplatin dosed on day 1 and gemcitabine dosed on days 1, 8, and 15, but subsequent studies have shown preserved efficacy and improved tolerability with a 21-day cycle with gemcitabine dosed on days 1 and 8 only.[10, 11] In addition, several studies have evaluated split-dose cisplatin (where cisplatin is dosed on days 1 and 8) of a 21-day cycle and found response rates similar to conventional dosing.[12, 13]

GC has also been used in the neoadjuvant setting. Memorial Sloan-Kettering Cancer Center has reported their experience of treating 42 patients with neoadjuvant GC demonstrating similar rates of pathological downstaging compared to historical controls (<pT2 rates of 36% in GC patients and 35% in MVAC patients).[14] In addition, rates of pT0 were also similar (26% in GC patients and 28% of MVAC patients). Given the improved tolerability and similar efficacy compared with MVAC, GC has become the most widely used neoadjuvant chemotherapy regimen for MIBC.

1.2.2 The Neoadjuvant Paradigm for Drug Development: An Ideal Model in Bladder Cancer

MIBC has been promoted as a model for drug development given the ease of pre- and post-treatment tissue collection without the need for research-directed biopsies.[15] Similar to the neoadjuvant trials in breast cancer, pT0 at the time of cystectomy in patients with MIBC treated with neoadjuvant cisplatin-based chemotherapy is associated with improved outcomes and predicts OS.[3, 16] A separate analysis has confirmed the prognostic significance of both pT0 and <pT2 at the time of cystectomy.[17] Assessment of pathological downstaging and complete pathologic response allows early feedback of drug efficacy with results in months rather than years.

1.2.3 Gemcitabine Cisplatin (GC) Regimen

<u>Gemcitabine</u>

Gemcitabine is a nucleoside analog of deoxycytidine that is transported into a cell, phosphorylated by deoxycytidine kinase to an active form, and incorporated into DNA causing an inhibition of replication and cell death. Gemcitabine has

been shown to attenuate immune tolerance achieved by many tumors by depleting myeloid-derived suppressor cells (MDSC) and regulatory T cells while preserving the number of CD4+ and CD8+ T cells in mice.[18-21] It has been suggested as useful in chemo-immunotherapy protocols due to its selective effects on the humoral immune response while not impairing antitumor cellular immunity.[2] The most common (≥20%) adverse reactions for gemcitabine when used as a single agent are nausea/vomiting, anemia, transaminitis, neutropenia, increased alkaline phosphatase, proteinuria, fever, hematuria, rash, thrombocytopenia, dyspnea, and peripheral edema.

Cisplatin

Cisplatin is a bifunctional alkylating agent that inhibits DNA synthesis and is cell cycle nonspecific. There is some preclinical data suggesting that cisplatin can also induce anti-tumor immunomodulation via recruitment and upregulation of effector T cells and downregulation of the immunosuppressive tumor microenvironment.[22]

Adverse effects reported in 10% or more of patients receiving cisplatin include peripheral neuropathy, nausea, vomiting, diarrhea, myelosuppression, liver enzymes elevation, nephrotoxicity (acute renal failure and chronic renal insufficiency), alopecia, tissue irritation, and ototoxicity. The nausea and vomiting associated with cisplatin are now much better tolerated due to the widespread availability and use of effective prophylactic anti-emetic regimens.

Rationale for the GC Regimen

As summarized in the individual drug sections, the GC regimen in combination with an immunotherapy agent, such as an immune checkpoint inhibitor, may enhance the modulatory effect of immunotherapy and assist in immune-mediated tumor destruction. The MVAC regimen incorporates methotrexate, which has known immunosuppressive effects and may theoretically deplete the effector T cell population causing an interference with the positive immunostimulatory effects of an immune checkpoint inhibitor. GC was chosen in this study due to its widespread use in the neoadjuvant setting, its safety profile, and potential synergistic effect with immunotherapy.

There are several effective dosing regimens of GC chemotherapy (see section 1.2.1). This protocol was initially implemented with cisplatin dosed as $70mg/m^2$ on day 1 of a 21-day cycle. However, a higher number than expected of grade 3-4 acute kidney injury and myelosuppression adverse events were noted in the first cohort of enrolled patients, and the protocol is therefore amended to dose cisplatin at $35mg/m^2$ on days 1 and 8 to minimize toxicity. In addition, the earlier cohort was treated with a single dose of pembrolizumab two weeks prior to concurrent GC and pembrolizumab treatment. This lead-in dose has also been omitted.

1.3 The PD-L1/PD-1 Pathway in Cancer

The PD-1 pathway is a major immune control switch which may be engaged by tumor cells to overcome active T-cell immune surveillance. The importance of intact immune surveillance in controlling outgrowth of neoplastic transformation has been known for decades.[23] Accumulating evidence shows a correlation between TILs in cancer tissue and favorable prognosis in various malignancies.[24-28] In particular, the presence of CD8+ T-cells and the ratio of CD8+ effector T-cells / FoxP3+ regulatory T-cells seems to correlate with improved prognosis and long-term survival in many solid tumors.

The PD-1 receptor-ligand interaction is a major pathway hijacked by tumors to suppress immune control. The normal function of PD-1, expressed on the cell surface of activated T-cells under healthy conditions, is to down-modulate unwanted or excessive immune responses, including autoimmune reactions. PD-1 (encoded by the gene Pdcd1) is an Ig superfamily member related to CD28 and CTLA-4 which has been shown to negatively regulate antigen receptor signaling upon engagement of its ligands (PD-L1 and/or PD-L2).[29, 30]

The structure of murine PD-1 has been resolved.[31] PD-1 and family members are type I transmembrane glycoproteins containing an Ig Variable-type (V-type) domain responsible for ligand binding and a cytoplasmic tail which is responsible for the binding of signaling molecules. The cytoplasmic tail of PD-1 contains 2 tyrosine-based signaling motifs, an immunoreceptor tyrosine-based inhibition motif (ITIM) and an immunoreceptor tyrosine-based switch motif (ITSM). Following T-cell stimulation, PD-1 recruits the tyrosine phosphatases SHP-1 and SHP-2 to the ITSM motif within its cytoplasmic tail, leading to the dephosphorylation of effector molecules such as CD3ζ, PKCθ and ZAP70 which are involved in the CD3 T-cell signaling cascade.[29, 32-34] The mechanism by which PD-1 down modulates T-cell responses is similar to, but distinct from that of CTLA-4 as both molecules regulate an overlapping set of signaling proteins.[35, 36]

PD-1 was shown to be expressed on activated lymphocytes including peripheral CD4+ and CD8+ T-cells, B-cells, Tregs and Natural Killer cells.[37, 38] Expression has also been shown during thymic development on CD4-CD8- (double negative) T-cells as well as subsets of macrophages and dendritic cells.[39] The ligands for PD-1 (PD-L1 and PD-L2) are constitutively expressed or can be induced in a variety of cell types, including non-hematopoietic tissues as well as in various tumors.[35, 40-42] Both ligands are type I transmembrane receptors containing both IgV- and IgC-like domains in the extracellular region and contain short cytoplasmic regions with no known signaling motifs. Binding of either PD-1 ligand to PD-1 inhibits T-cell activation triggered through the T-cell receptor. PD-L1 is expressed at low levels on various non-hematopoietic tissues, most notably on vascular endothelium, whereas PD-L2 protein is only detectably expressed on antigen-presenting cells found in lymphoid tissue or chronic inflammatory environments. PD-L2 is thought to control immune T-cell activation in lymphoid organs, whereas PD-L1 serves to dampen unwarranted T-

cell function in peripheral tissues.[35] Although healthy organs express little (if any) PD-L1, a variety of cancers were demonstrated to express abundant levels of this T-cell inhibitor. PD-1 has been suggested to regulate tumor-specific T-cell expansion in subjects with melanoma.[43] This suggests that the PD-1/PD-L1 pathway plays a critical role in tumor immune evasion and should be considered as an attractive target for therapeutic intervention.

1.3.1 Bladder Cancer and Immunotherapy

Bladder cancer was the first malignancy with an FDA-approved immunotherapy when Bacillus Calmette-Guérin (BCG), a live but attenuated strain of mycobacterium, was approved for localized disease in 1990. Advanced bladder cancer is associated with a high mutational complexity and the potential for foreign antigen detection by the immune system represents a unique opportunity for development of immunotherapeutics.[44, 45]

1.3.2 Immune Checkpoint Inhibitors in Bladder Cancer

Anti-PD-1 and PD-L1 agents have demonstrated exciting activity in several human cancers (most notably lung, renal cell, and melanoma) with an overall response rate of 21% in human cancers.[46, 47] Initial data from studies in the metastatic setting of PD-1 and PD-L1 inhibition show activity of these agents in bladder cancer as well. [48, 49] Recently, several immune checkpoint inhibitors have been approved for the treatment of metastatic urothelial cancer that has progressed after first-line platinum-based chemotherapy. Atezolizumab (anti-PD-L1) was first approved in May 2016 after a large phase II trial showed an overall response rate (ORR) of 15%.[50] It has since been approved in the first-line metastatic setting for cisplatin-ineligible patients.[51] Nivolumab, durvalumab, and avelumab were also approved in early 2017 based on data from several phase II trials demonstrating ORR of 19.6%. [52, 53]

Initial data from a phase Ib study of pembrolizumab in patients with metastatic bladder cancer showed an ORR of 24% with a median time to response of only 8 weeks with several durable responses noted (median duration of response not yet reached).(NCT01848834)[49] Subsequently, the Keynote-045 trial randomized patients with metastatic UC that had progressed after platinum-based chemotherapy to pembrolizumab or investigator's choice chemotherapy (paclitaxel, docetaxel, or vinflunine).[54] Treatment with pembrolizumab was associated with an improvement in overall survival compared with chemotherapy. Therefore, immune checkpoint inhibition is a new standard of care for second-line treatment of metastatic UC and is approved for use in the first-line setting in cisplatin-ineligible patients.

1.4 Pembrolizumab

Pembrolizumab is a potent and highly selective humanized monoclonal antibody (mAb) of the IgG4/kappa isotype designed to directly block the interaction between PD-1 and its ligands, PD-L1 and PD-L2. Keytruda™ (pembrolizumab)

has recently been approved in the United Stated for the treatment of patients with unresectable or metastatic melanoma, non-small cell lung cancer with high PD-L1 expression, head and neck squamous cell carcinoma, and classical Hodgkin lymphoma.

A detailed summary of preclinical and clinical data for pembrolizumab is provided in the Investigator's Brochure. Relevant clinical safety and pharmacokinetic data supporting this proposed study are briefly summarized in subsequent sections.

1.4.1 Pembrolizumab Clinical Findings

Rationale for Pembrolizumab Clinical Dose (200 mg IV Q3W):

An open-label Phase I trial (Protocol 001) is being conducted to evaluate the safety and clinical activity of single agent MK-3475. The dose escalation portion of this trial evaluated three dose levels, 1 mg/kg, 3 mg/kg, and 10 mg/kg, administered every 2 weeks (Q2W) in subjects with advanced solid tumors. All three dose levels were well tolerated and no dose-limiting toxicities were observed. This first in human study of MK-3475 showed evidence of target engagement and objective evidence of tumor size reduction at all dose levels (1 mg/kg, 3 mg/kg and 10 mg/kg Q2W). No MTD has been identified to date. 10.0 mg/kg Q2W, the highest dose tested in PN001, will be the dose and schedule utilized in Cohorts A, B, C and D of this protocol to test for initial tumor activity. Recent data from other clinical studies within the MK-3475 program has shown that a lower dose of MK-3475 and a less frequent schedule may be sufficient for target engagement and clinical activity.

PK data analysis of MK-3475 administered Q2W and Q3W showed slow systemic clearance, limited volume of distribution, and a long half-life (refer to IB). Pharmacodynamic data (IL-2 release assay) suggested that peripheral target engagement is durable (>21 days). This early PK and pharmacodynamic data provides scientific rationale for testing a Q2W and Q3W dosing schedule.

A population pharmacokinetic analysis has been performed using serum concentration time data from 476 patients. Within the resulting population PK model, clearance and volume parameters of MK-3475 were found to be dependent on body weight. The relationship between clearance and body weight, with an allometric exponent of 0.59, is within the range observed for other antibodies and would support both body weight normalized dosing or a fixed dose across all body weights. MK-3475 has been found to have a wide therapeutic range based on the melanoma indication. The differences in exposure for a 200 mg fixed dose regimen relative to a 2 mg/kg Q3W body weight based regimen are anticipated to remain well within the established exposure margins of 0.5 – 5.0 for MK-3475 in the melanoma indication. The exposure margins are based on the notion of similar

efficacy and safety in melanoma at 10 mg/kg Q3W vs. the proposed dose regimen of 2 mg/kg Q3W (i.e. 5-fold higher dose and exposure). The population PK evaluation revealed that there was no significant impact of tumor burden on exposure. In addition, exposure was similar between the NSCLC and melanoma indications. Therefore, there are no anticipated changes in exposure between different indication settings.

The rationale for further exploration of 2 mg/kg and comparable doses of pembrolizumab in solid tumors is based on: 1) similar efficacy and safety of pembrolizumab when dosed at either 2 mg/kg or 10 mg/kg Q3W in melanoma patients, 2) the flat exposure-response relationships of pembrolizumab for both efficacy and safety in the dose ranges of 2 mg/kg Q3W to 10 mg/kg Q3W, 3) the lack of effect of tumor burden or indication on distribution behavior of pembrolizumab (as assessed by the population PK model) and 4) the assumption that the dynamics of pembrolizumab target engagement will not vary meaningfully with tumor type.

The choice of the 200 mg Q3W as an appropriate dose for the switch to fixed dosing is based on simulations performed using the population PK model of pembrolizumab showing that the fixed dose of 200 mg every 3 weeks will provide exposures that 1) are optimally consistent with those obtained with the 2 mg/kg dose every 3 weeks, 2) will maintain individual patient exposures in the exposure range established in melanoma as associated with maximal efficacy response and 3) will maintain individual patients exposure in the exposure range established in melanoma that are well tolerated and safe.

A fixed dose regimen will simplify the dosing regimen to be more convenient for physicians and to reduce potential for dosing errors. A fixed dosing scheme will also reduce complexity in the logistical chain at treatment facilities and reduce wastage.

Pembrolizumab Clinical Safety:

As of Mar 2018 pembrolizumab monotherapy and combination therapy have been administered to 25,519 subjects, with hematologic malignancies and solid tumors. Safety data are available for a total of 2799 subjects in 4 Merck-sponsored clinical trials. The majority of participants, 2727 or 97.4%, experienced 1 or more AEs, and 2062(73.7%) experienced 1 or more AEs reported as drug-related by the investigator. The most commonly reported AEs reported in monotherapy trials were fatigue (37.3%), nausea (24.5%), decreased appetite (22.5%), diarrhea (22.3%), and cough (22.0%).[55]

Safety of Combinations of Pembrolizumab and Chemotherapy:

There are nine Merck-sponsored pembrolizumab + chemotherapy trials that are ongoing based on recent information provided in the IB [48]. For example, 5 Phase I/II studies are ongoing evaluating the combination of pembrolizumab with

chemotherapy, including a study combining pembrolizumab with either cisplatin and pemetrexed or carboplatin and paclitaxel in non-small cell lung cancer (NCT01840579). A phase III trial of pembrolizumab vs pembrolizumab plus 5-FU plus cisplatin or carboplatin vs cetuximab plus 5-FU plus cisplatin or carboplatin in recurrent or metastatic head and neck cancer is actively recruiting subjects (NCT02358031). Another phase Ib/II study of neoadjuvant pembrolizumab in combination with either cisplatin and gemcitabine or gemcitabine alone is also currently recruiting patients with muscle-invasive bladder cancer (NCT02365766). Another study is ongoing evaluating nivolumab, a PD-L1 antibody with similar theoretical toxicity to pembrolizumab, in combination with gemcitabine and cisplatin in patients with non-small cell lung cancer (NCT 01454102).

Safety data encompassing all subjects from these trials is pending. However, the major toxicities of pembrolizumab including fatigue, constipation, nausea, decreased appetite, and diarrhea, should not overlap with the major toxicities of gemcitabine and cisplatin as outlined above, and the mechanisms of action are distinct. Therefore, no serious safety issues are anticipated as a result of this combination given the low likelihood of overlapping toxicities. Any unexpected toxicities from the chemotherapy combination trials just summarized and sponsored by Merck will be shared with the PI of LCCC1520 and can be used to supplement toxicity monitoring if needed. The optimal timing of the administration of pembrolizumab, gemcitabine, and cisplatin in combination is not entirely clear.

1.5 Rationale for Inclusion of Pembrolizumab as NAC for MIBC

Despite a proven survival benefit with NAC in MIBC, utilization remains low and more than half of patients develop recurrent or metastatic disease and die within 2 years.[56] More effective and tolerable perioperative regimens are desperately needed.

The inhibition of antitumor cytotoxic T cell activity via PD-1/PD-L1 interaction is a major immunosuppressant mechanism of many cancers and inhibition of this blockade leads to an anticancer immune response. Pembrolizumab has shown promising results in metastatic bladder cancer as well as several other solid tumor cancers. This study expands the application of pembrolizumab immunotherapy to the neoadjuvant setting, potentially adding efficacy without substantial toxicity to the treatment of MIBC. Inhibition of immune evasion by tumor cells, and therefore activation of cytotoxic T-cells, may have an additive effect to the gemcitabine and cisplatin combination chemotherapy in the neoadjuvant setting. Further, the anti-PD-1 activated T cells may progress toward a memory phenotype, which is more refractory to tolerance, longer living, and has greater anti-cancer activity. These memory cells may continue to expand, recognize, and kill micrometastases, which account for relapses despite the neoadjuvant chemotherapy and surgical extirpation.

Study Endpoints: Pathologic downstaging after NAC can be defined as absence of any residual muscle-invasive disease at the time of cystectomy (<pT2) and includes pT1, pTcis, pTa, and T0 disease. Due to the multifocal nature of this disease, it is very common at both pre-chemotherapy and post-chemotherapy evaluations to see pre-existing and/or residual superficial transitional cell carcinoma such as Tcis, Ta, or T1 disease (frequently collectively defined as <pT2). The likelihood of residual superficial disease is also directly related to the extent of the TURBT prior to NAC. Superficial disease, and in particular Tcis, is typically unresponsive to chemotherapy, non-life threatening, and frequently included within the definition of major chemotherapy response.

The prognostic value of defining response to NAC as <pT2 as opposed to complete pathologic response (pT0) was initially shown in an analysis by Splinter et al of 147 patients with MIBC treated at eight different centers.[57] This study demonstrated that patients who achieved <pT2 after NAC achieved 75% 5-year survival in contrast to 20% 5-year survival in those whose tumors were still muscle-invasive (≥pT2 residual disease).

pT0 at the time of radical cystectomy is also associated with long-term survival outcomes in MIBC as demonstrated in the SWOG 8710 trial.[3] An independent study at MSKCC has validated comparable survival rates of patients achieving <pT2 and pT0.[58] A subsequent analysis confirmed both pT0 and <pT2 were associated with survival. Those with pT0 had a median OS of 13.6 years, those with <pT2 (but not pT0) had a median OS of 10.6 years, and those with pT2 or greater disease had a median OS of 3.7 years.[17] The use of pathological downstaging and pathologic complete response rate as study endpoints allows for initial efficacy data to be collected shortly after completion of neoadjuvant chemotherapy, allowing for earlier data interpretation than traditional time to event endpoints such as recurrence-free survival and overall survival.

1.6 Inclusion of Patient-Reported Outcomes version of the Common Terminology Criteria for Adverse Events (PRO-CTCAE)

Adverse event reporting has historically been collected during clinical trials using the provider-reported common terminology criteria for adverse events (CTCAE). The National Cancer Institute (NCI) has been developing a system for patient self-reporting of symptom adverse events (AE) listed in the CTCAE in an effort to improve the accuracy and precision of grading this class of AEs. The PRO-CTCAE is incorporated in this trial along with the CTCAE to improve the accuracy of symptom toxicity monitoring. In addition, several questions from the Patient Reported Outcomes Measurement Information System (PROMIS) are utilized as a general measure of the patients' state of wellbeing.

1.7 Correlative Studies

1.7.1 Predictive and Prognostic Biomarkers in Bladder Cancer

There are many recent examples of predictive and prognostic biomarkers in other types of cancer (HER2/neu overexpression and trastuzumab response in breast cancer, for example). However, no current molecular markers are widely used for targeted therapy in bladder cancer.

MIBC is, with rare exceptions, classified as high-grade (versus low-grade) via pathological review, using the consensus system developed by The World Health Organization and the International Society of Urologic Pathologists.[59] RNA subtypes of high grade bladder cancer have been recently described including a unique gene expression pattern using a 47-gene signature (BASE47) recently described by investigators at the University of North Carolina (UNC) (Kim – co-PI). These subtypes have been named "basal" and "luminal" subtypes due to similarities with the intrinsic subtypes of breast cancer.[60] The basal subtype has been associated with more aggressive disease (i.e., may be prognostic), although has also recently been reported to have a prolonged OS after neoadjuvant chemotherapy in MIBC (i.e., may also be predictive).[61] A subset of basal and claudin-low subtypes of MIBCs express biomarkers reflective of high immune cellular content, and T cell modulators may be more active in this subset (claudin-low subtype data unpublished).[62] It is therefore theorized that subtypes of high-grade bladder cancer may predict response to anti-PD-1 and anti-PD-L1 therapies.

1.7.2 Planned Biomarker Studies for LCCC1520

All patients will have pre- and post-treatment tumor tissue submitted for correlative studies. Tissue from primary tumor samples (pre-treatment and tumor tissue at cystectomy) and lymph nodes removed at cystectomy will be processed for RNA extraction and generation of formalin-fixed paraffin embedded (FFPE) blocks/slides. See section 6.7 and the accompanying laboratory manual for details.

Through our planned correlative studies, we hope to gather preliminary data on 1) *potential determinants of response to pembrolizuma*b and 2) *changes in the immune microenvironment driven by pembrolizumab therapy.*

The basal and claudin-low molecular subtypes of bladder cancer are enriched in tumor-infiltrating immune cells with a gene-expression signature consistent with active immunosuppression.[63] This study will explore the hypothesis that the basal subtype classification will be associated with response to pembrolizumab via correlation with the presence of substantial tumor immune infiltrate.

In addition to the basal and claudin-low molecular subtype, other pre-treatment tumor features we hypothesize will be associated with response include:
1) Increased mRNA expression of PD-L1,
2) Increased expression of general immune gene signatures and signatures associated with immune regulation,
3) Clonal restriction in T-cell receptor (TCR) and B-cell receptor (BCR) repertoires,

4) Increased tumor infiltration with effector cells of multiple lineages localized intratumorally or peritumorally in regions staining heavily for PD-L1, and
5) Decreased tumor infiltration by regulatory cells of multiple lineages. Patients who respond to therapy that have lymph nodes assayable at the time of cystectomy will show similar findings. We also hypothesize that certain TCR and BCR repertoire characteristics will be associated with response and will be enriched in the basal vs. luminal subtypes.

2.0 STUDY OBJECTIVES

2.1 Primary Objective

To estimate the proportion of patients with pathological downstaging to <pT2 after neoadjuvant therapy with pembrolizumab in combination with gemcitabine and cisplatin in patients with MIBC.

Hypothesis: The rate of pathologic downstaging to <pT2 will improve compared to historical controls

2.2 Secondary Objectives

2.2.1 To estimate the proportion of patients with pT0 after neoadjuvant therapy with pembrolizumab when administered in combination with gemcitabine and cisplatin in patients with MIBC

Hypothesis: The rate of pathologic downstaging to pT0 will improve compared to historical controls

2.2.2 To estimate event free survival (EFS)

Hypothesis: EFS will be improved compared to historical controls

2.2.3 To estimate overall survival (OS)

Hypothesis: OS will be improved compared to historical controls

2.2.4 To estimate the proportion of patients who are alive at 3 years (OS at 3 years)

Hypothesis: OS at 3 years will be improved compared to historical controls

2.2.5 To characterize the toxicity profile for the combination of pembrolizumab, gemcitabine, and cisplatin

Hypothesis: Pembrolizumab, gemcitabine, and cisplatin will be a safe combination with acceptable toxicity rates

[redacted]

2.4 Endpoints

2.4.1 Primary Endpoint

Pathological downstaging to <pT2 is defined as pT0-T1N0M0 at the time of cystectomy

2.4.2 Secondary Endpoints

- Complete pathologic response rate (pT0) is defined as pT0N0M0 at the time of cystectomy
- EFS is defined from D1 of neoadjuvant treatment until progression (in those who progress prior to surgery) or until recurrence (post-surgery) or until death as a result of any cause. Recurrence of disease includes second primary urothelial carcinoma malignancies.
- OS is defined as the time from D1 of treatment until death as a result of any cause
- OS at 3 years is defined from D1 of treatment until 3 years or death as a result of any cause
- Safety and toxicity will be characterized according to the reported adverse event (AE) profile using NCI Common Terminology Criteria for Adverse Events (CTCAE) v4.03, as well as a patient questionnaire derived from the Patient Reported Outcomes (PRO)-CTCAE and Patient Reported Outcomes Measurement Information System (PROMIS).

3.0 PATIENT ELIGIBILITY

3.1 Inclusion Criteria

The subject must meet all of the inclusion criteria to participate in this study:

3.1.1 Be able to give written IRB approved informed consent and be able to follow protocol requirements.

3.1.2 Be ≥ 18 years of age on day of signing informed consent.

3.1.3 Has a performance status of 0 or 1 on the ECOG Performance Scale (see section 11.1, Appendix A).

3.1.4 Has histologically confirmed urothelial carcinoma of the bladder; those with mixed histology, including a component of urothelial carcinoma, are eligible. Pure small cell carcinoma, pure adenocarcinoma, and pure squamous cell carcinoma are excluded.

3.1.5 Has clinical stage T2-T4a N0/X M0 urothelial carcinoma. Clinical T stage is based on the pre-study standard of care transurethral resection of the bladder tumor (TURBT) sample and imaging studies.

System	Laboratory Value
**Hematological**	
Absolute neutrophil count (ANC)	≥1,500 /mcL
Platelets	≥100,000 / mcL
Hemoglobin	≥9 g/dL or ≥5.6 mmol/L without transfusion or EPO dependency (within 7 days of assessment)
**Renal**	
Serum creatinine<br>**OR**<br>Estimated GFR per CKD-EPI equation[a]	≤1.5 X upper limit of normal (ULN)<br>**OR**<br>≥60 mL/min for subject with creatinine levels > 1.5 X institutional ULN
**Hepatic**	
Serum total bilirubin	≤ 1.5 X ULN (≤ 3 X ULN if Gilbert's Syndrome) **OR** Direct bilirubin ≤ ULN for subjects with total bilirubin levels > 1.5 ULN
AST (SGOT) and ALT (SGPT)	≤ 2.5 X ULN
**Coagulation**	
International Normalized Ratio (INR) or Prothrombin Time (PT)<br>Activated Partial Thromboplastin Time (aPTT)	≤1.5 X ULN unless subject is receiving anticoagulant therapy. If subject receiving anticoagulants, PT or PTT should be within therapeutic range of intended use of anticoagulants

[a]Creatinine clearance should be calculated per CKD-EPI equation[64]

3.1.6 Has staging scans with abdominal/pelvic CT or MRI scan and CT scan or X-ray of the chest within 4 weeks prior to treatment initiation

3.1.7 Be a medically appropriate candidate for radical cystectomy as determined by an attending urologist and be planning to receive cystectomy.

3.1.8 Has had no prior systemic cytotoxic chemotherapy for urothelial carcinoma (prior intravesicular chemotherapies are permitted)

3.1.9 Patients must agree to submission of tumor tissue from transurethral resection of the bladder tumor (TURBT) including a paraffin block or 20 formalin-fixed paraffin embedded (FFPE) slides of 5-10 microns in thickness. Patients must also agree to submission of tissue from cystectomy.

3.1.10 Demonstrate adequate organ function as defined in the table below; all screening labs should be performed within 10 days of treatment initiation.

3.1.11 Female subjects of childbearing potential should have a negative urine or serum pregnancy within 72 hours prior to receiving the first dose of pembrolizumab. If the urine test is positive or cannot be confirmed as negative, a serum pregnancy test will be required.

3.1.12 Female subjects of childbearing potential must be willing to adequate method of contraception as outlined in Section 5.1.11 – Contraception, for the course of the study through 120 days after the last dose of study medication.

Note: Abstinence is acceptable if this is the usual lifestyle and preferred contraception for the subject.

3.1.13 Male subjects of childbearing potential must agree to use an adequate method of contraception as outlined in Section 5.1.11 - Contraception, starting with the first dose of study therapy through 120 days after the last dose of study therapy.

Note: Abstinence is acceptable if this is the usual lifestyle and preferred contraception for the subject.

3.1.14 Life expectancy greater than 3 months

3.1.15 Consents to whole blood collection prior to initiating therapy, on cycle 2 day 1, and at cystectomy for support of correlative research studies

3.2 Exclusion Criteria

The subject must be excluded from participating in the trial if the subject meets any of the following:

3.2.1 Is currently participating in or has participated in a study of an investigational agent or using an investigational device within 4 weeks of the first dose of pembrolizumab.

3.2.2 Has a diagnosis of immunodeficiency or is receiving systemic steroid therapy or any other form of immunosuppressive therapy within 7 days prior to the first dose of trial treatment**. NOTE**: see exception to use of systemic steroid as prophylactic anti-emetic prior to cisplatin in section 4.2.2. Inhaled and topical steroids are allowed.

3.2.3 Has a known history of active TB (Bacillus Tuberculosis)

3.2.4 Hypersensitivity to pembrolizumab or any of its excipients.

3.2.5 Has had a prior monoclonal antibody within 4 weeks prior to study Day 1 or who has not recovered (i.e., ≤ Grade 1 or at baseline) from adverse events due to agents administered more than 4 weeks earlier.

3.2.6 Has had prior chemotherapy, targeted small molecule therapy, or radiation therapy within 2 weeks prior to study Day 1 or who has not recovered (i.e., ≤ Grade 1 or at baseline) from adverse events due to a previously administered agent.

- **Note:** If subject received major surgery, they must have recovered adequately from the toxicity and/or complications from the intervention prior to starting therapy.

3.2.7 Has a known additional malignancy that is progressing or requires active treatment. Exceptions include basal cell carcinoma of the skin, squamous cell carcinoma of the skin, or in situ cervical cancer that has undergone potentially curative therapy.

3.2.8 Has active autoimmune disease that has required systemic treatment in the past 2 years (i.e. with use of disease modifying agents, corticosteroids or immunosuppressive drugs). Replacement therapy (eg., thyroxine, insulin, or physiologic corticosteroid replacement therapy for adrenal or pituitary insufficiency, etc.) is not considered a form of systemic treatment.

3.2.9 Has a history of (non-infectious) pneumonitis that required steroids or current pneumonitis.

3.2.10 Has an active infection requiring systemic therapy

3.2.11 Has a history or current evidence of any condition, therapy, or laboratory abnormality that might confound the results of the trial, interfere with the subject's participation for the full duration of the trial, or is not in the best interest of the subject to participate, in the opinion of the treating investigator.

3.2.12 Has known psychiatric or substance abuse disorders that would interfere with cooperation with the requirements of the trial

3.2.13 Is pregnant or breastfeeding or expecting to conceive or father children within the projected duration of the trial, starting with the pre-screening or screening visit through 120 days after the last dose of trial treatment.

3.2.14 Has received prior therapy with an anti-PD-1, anti-PD-L1, anti-PD-L2, anti-CD137, or anti-Cytotoxic T-lymphocyte-associated antigen-4 (CTLA-4) antibody (including ipilimumab or any other antibody or drug specifically targeting T-cell co-stimulation or checkpoint pathways).

3.2.15 Has received prior radiation therapy to the bladder for the purpose of treating urothelial carcinoma.

3.2.16 Has a known history of Human Immunodeficiency Virus (HIV) (HIV 1/2 antibodies).

3.2.17 Has known active Hepatitis B (e.g., HBsAg reactive) or Hepatitis C (e.g., HCV RNA [qualitative] is detected).

3.2.18 Has clinically relevant hearing impairment > Grade 2

3.2.19 Has received a live vaccine within 30 days prior to the first dose of trial treatment

4.0 TREATMENT PLAN

4.1 Schema

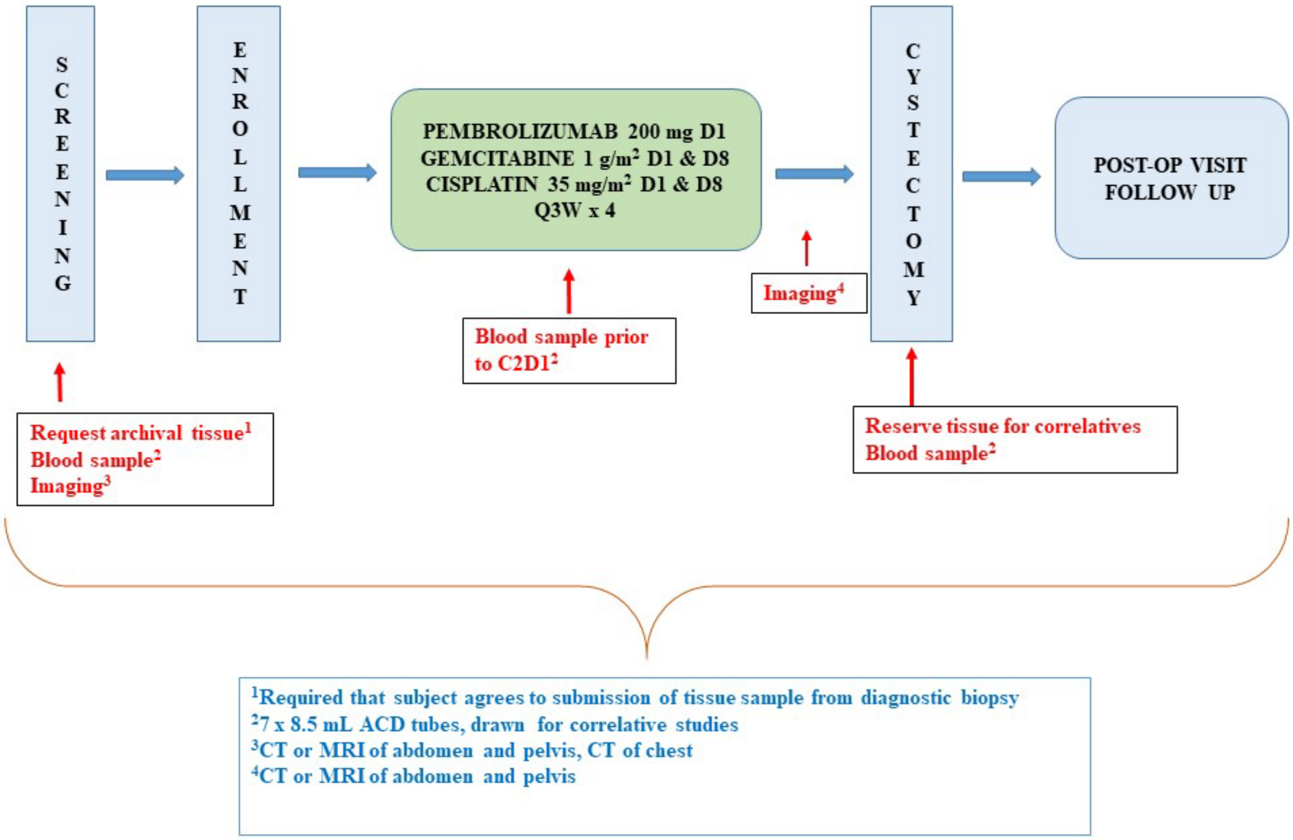

This is a phase II, open-label, single arm study evaluating pembrolizumab administered in combination with gemcitabine and cisplatin in the neoadjuvant setting prior to radical cystectomy in patients with MIBC. Toxicity will be assessed during treatment using NCI CTCAE v4.03, PRO-CTCAE and PROMIS. A tumor specimen (fresh frozen specimen or FFPE or both) from a pre-study transurethral resection of bladder tumor (TURBT; performed as per routine clinical care) and post-treatment surgical specimen (fresh frozen tissue if possible, although FFPE acceptable, or both) will be collected for correlative studies.

Pembrolizumab 200mg IV is administered IV on day 1 in combination with cisplatin 35mg/m^2 and gemcitabine 1000mg/m^2 on day 1 and day 8 every 3 weeks for 4 cycles over 12 weeks.

4.2 Treatment Dosage and Administration

A window of +/- 3 days will apply to each study visit (see Time and Events table), except Day 8 visits each cycle will have a window of +/- 1 day.

Trial treatment should be administered on Day 1 of each cycle after all procedures/assessments have been completed as detailed on the Time and Events table (Section 6.1). Trial treatment may be administered up to 3 days before or after the scheduled Day 1 of each cycle due to administrative reasons. Day 8 visits will have a window of +/- 1 day.

All trial treatments will be administered on an outpatient basis.

After screening and enrollment, patients will receive: (see section 4.2.1, Pembrolizumab Administration below, for details on administration of pembrolizumab):
Pembrolizumab 200mg IV on Day 1 every 3 weeks for 4 cycles
Gemcitabine 1000mg/m^2 IV on Day 1 and Day 8 every 3 weeks for 4 cycles
Cisplatin 35mg/m^2 IV on Day 1 and Day 8 every 3 weeks for 4 cycles.

This will total 12 weeks of neoadjuvant therapy.
On Day 1 of each cycle pembrolizumab will be administered first, followed by gemcitabine and cisplatin. Each site must follow their institutional guidelines for administration of each drug and hydration.

Concomitant GC + Pembrolizumab Therapy

**Drug**	**Dose**	**Route**	**Schedule**	**Cycle Length & Number**	**Use**
Pembrolizumab	200 mg	IV over 30 minutes	Day 1	Every 21 days x 4 cycles	Experimental
Gemcitabine	1000 mg/m^{2*}	IV over 1 hour***	Days 1,8		Standard
Cisplatin	35 mg/m^{2*}	IV over 1 hour***	Day 1, 8		Standard
*In select patients**:* Pegfilgrastim<br><br>*Alternative to pegfilgrastim:* Filgrastim	6 mg<br><br>5mcg/day	SC<br><br>SC	Day 9<br><br>Days 9-13		

*Dose on total body weight
** Use of growth factor support (pegfilgrastim or filgrastim) should be based on age (notable age 65 and older), preexisting conditions (neutropenia at baseline, etc), and other patient risk factors for the development of neutropenic fever. Pegfilgrastim may be included with the first cycle of this regimen at the treating physician's discretion and institutional practice. Additionally, according to institutional

practice, filgrastim may be used as an alternative to pegfilgrastim in accordance with local practice. Duration of filgrastim administration may be altered based on patient response or institutional practice.
*** Duration of gemcitabine and cisplatin may be altered per institutional practice

4.2.1 Dose Selection

The rationale for selection of doses to be used in this trial is provided in Section 1.4.1.

4.2.2 Anti-Emetics and Hydration Prior to and Post Cisplatin

Appropriate pre- and post-hydration for cisplatin chemotherapy will be included according to institutional guidelines. Furosemide, mannitol, and electrolyte supplementation may be included according to local practices as well. Anti-emetic prophylaxis should be administered with cisplatin according to the following table and are recommended to include a corticosteroid and a 5-HT3 antagonist. Corticosteroids should only be used as pre- and post-medication for antiemetic prophylaxis for cisplatin therapy and should be dosed as delineated below.

Chemotherapy	Premed Day	Premeds	Suggested Home Regimen
Cisplatin and Gemcitabine	Day 1 and 8	Dexamethasone 8mg IV or po<br>and<br>5-HT3 antagonist<br>and/or<br>Lorazepam	Dexamethasone 8mg po days 2,3 9, and 10<br>AND<br>5-HT3 antagonist prn<br>and/or<br>Lorazepam prn

4.2.3 Pembrolizumab Administration

Pembrolizumab 200 mg will be administered as a 30-minute IV infusion. Sites should make every effort to target infusion timing to be as close to 30 minutes as possible. However, given the variability of infusion pumps from site to site, a window of -5 minutes and +10 minutes is permitted (i.e., infusion time is 30 minutes: -5 min/+10 min).

Management of Infusion Reactions: Signs and symptoms usually develop during or shortly after drug infusion and generally resolve completely within 24 hours of completion of infusion. Refer to the table below for infusion reaction treatment guidelines:

NCI CTCAE Grade	Treatment	Premedication at subsequent dosing
Grade 1<br>Mild reaction; infusion interruption not indicated; intervention not indicated	Increase monitoring of vital signs as medically indicated until the subject is deemed medically stable in the opinion of the investigator.	None
Grade 2<br>Requires infusion interruption but responds promptly to	**Stop Infusion and monitor symptoms.**<br>Additional appropriate medical therapy may include but is not limited to:	Subject may be premedicated 1.5h (± 30 minutes) prior to

NCI CTCAE Grade	Treatment	Premedication at subsequent dosing
symptomatic treatment (e.g., antihistamines, NSAIDS, narcotics, IV fluids); prophylactic medications indicated for < =24 hrs	IV fluids<br>Antihistamines<br>NSAIDS<br>Acetaminophen<br>Narcotics<br>Increase monitoring of vital signs as medically indicated until the subject is deemed medically stable in the opinion of the investigator.<br>If symptoms resolve within one hour of stopping drug infusion, the infusion may be restarted at 50% of the original infusion rate (e.g., from 100 mL/hr to 50 mL/hr). Otherwise dosing will be held until symptoms resolve and the subject should be premedicated for the next scheduled dose.<br>**Subjects who develop Grade 2 toxicity despite adequate premedication should be permanently discontinued from further trial treatment administration.**	infusion of pembrolizumab (MK-3475) with:<br><br>Diphenhydramine 50 mg po (or equivalent dose of antihistamine).<br><br>Acetaminophen 500-1000 mg po (or equivalent dose of antipyretic).
Grades 3 or 4<br><br>Grade 3:<br>Prolonged (i.e., not rapidly responsive to symptomatic medication and/or brief interruption of infusion); recurrence of symptoms following initial improvement; hospitalization indicated for other clinical sequelae (e.g., renal impairment, pulmonary infiltrates)<br><br>Grade 4:<br>Life-threatening; pressor or ventilatory support indicated	**Stop Infusion.**<br>Additional appropriate medical therapy may include but is not limited to:<br>IV fluids<br>Antihistamines<br>NSAIDS<br>Acetaminophen<br>Narcotics<br>Oxygen<br>Pressors<br>Corticosteroids<br>Epinephrine<br><br>Increase monitoring of vital signs as medically indicated until the subject is deemed medically stable in the opinion of the investigator.<br>Hospitalization may be indicated.<br>**Subject is permanently discontinued from further trial treatment administration.**	No subsequent dosing
Appropriate resuscitation equipment should be available in the room and a physician readily available during the period of drug administration.		

4.3 Timing of Cystectomy after Chemotherapy

Enrollment into LCCC1520 should not impact this standard of care procedure. This procedure should be performed 4-8 weeks, if possible, from the last dose of

neoadjuvant chemotherapy with recovery from all associated toxicities to grade 1 or less at the time of surgery. At minimum, a standard lymph node dissection must be performed at the time of radical cystectomy, as per standard of care.

4.4 Toxicities and Dosing Delays/Dose Modifications

Any patient who receives treatment on this protocol will be evaluable for toxicity. Each patient will be assessed periodically for the development of any toxicity according to the Time and Events table (Section 6.1). Toxicity will be assessed according to the NCI CTCAE, version 4.0, the PRO-CTCAE, and PROMIS for 30 days after dosing is completed. Serious adverse events (SAEs) and Events of Clinical Interest (ECI) will be followed for 90 days after dosing is completed. Dose adjustments should be made according to the system showing the greatest degree of toxicity according to the NCI CTCAE, version 4.0. The patient-reported adverse events (collected via the PRO-CTCAE and PROMIS) will be assessed concurrently with the CTCAE, and dose modification or delay can be considered by the treating physician according to the highest patient-reported grade of toxicity.

Per the dose modification tables below, if administration of either cisplatin or gemcitabine on Day 1 is delayed, the other chemotherapy agent will also be held, and a new Day 1 will be scheduled. When that occurs, pembrolizumab should also be held until the new Day 1 based on gemcitabine/cisplatin is scheduled. Unless otherwise noted in the tables, treatment may be delayed for ≤ 2 weeks for any reason. If Day 1 treatment is delayed ≤ 2 weeks, subjects will proceed with the current cycle of treatment at the dose level recommended according to the tables below. The reason for interruption should be documented in the medical record and treatment form. If >2 week delay is required, or if cumulative delays of >4 weeks are required, discontinue the subject from protocol-mandated treatment but maintain follow-up per protocol.

On any given cycle, day 8 treatment is given only if day 1 treatment has been administered. Per the tables below, day 8 treatment should be held due to toxicity as outlined and held day 8 doses will not be made up.

On day 1 of each cycle, if pembrolizumab is held due to toxicity but the patient is eligible to receive cisplatin and gemcitabine based on the tables below, ONLY pembrolizumab should be held and patients should receive gemcitabine and cisplatin as scheduled. Pembrolizumab should be held for the duration of that cycle and restarted (if applicable) on day 1 of the subsequent cycle. If pembrolizumab is permanently discontinued, the subject should be discontinued from protocol-mandated treatment but maintain follow-up per protocol.

For the purposes of required dose reductions with gemcitabine, refer to the dose levels below. If more than 2 dose reductions of gemcitabine and/or more than 1 dose reduction of cisplatin are required, discontinue patient from protocol directed

treatment and follow-up per protocol. Note that once a drug dose is reduced, there is no re-escalation to a previous dosing level.

**Cisplatin Dose Levels**	
**Dose Level**	**Cisplatin Dose**
Level 0	35mg/m^2 on Day 1 and Day 8
Level -1	25mg/m^2 on Day 1 and Day 8

**Gemcitabine Dose Levels**	
**Dose Level**	**Gemcitabine Dose**
Level 0	1000 mg/m^2 on Day 1 and Day 8
Level -1	750 mg/m^2 on Day 1 and Day 8
Level -2	500 mg/m^2 on Day 1 and Day 8

No doses lower than cisplatin 25mg/m^2 or gemcitabine 500mg/m^2 will be permitted.

4.4.1 Dose Modifications for GC for Hematologic Toxicity

**Hematological Toxicity Dose Reductions for Cisplatin**	
**Labs**	**Action**
ANC ≥ 1,500/µL on Day 1	None
ANC <1500/µL on Day 1	For ANC <1500/µL on day 1, delay cisplatin until the ANC ≥ 1500/µL. If the delay is one week or less, continue cisplatin at the same dose and consider pegfilgrastim with subsequent cycles, if not receiving. If the delay is <u>for more than a week</u>* but ≤14 days and pegfilgrastim has not been used, include pegfilgrastim in all subsequent cycles. If there is a dose delay for <u>more than a week* due to neutropenia despite pegfilgrastim use</u> but ≤14 days, reduce cisplatin by one dose level for this and all subsequent cycles and continue pegfilgrastim
Platelets ≥ 100,000/µL on Day 1	None
Platelets < 100,000/µL on Day 1	For platelets <100,000 on day 1, delay cisplatin* until platelets ≥ 100,000, then continue cisplatin at the same dose
Platelets ≥ 75,000/µL on Day 8	None
Platelets < 75,000/µL but ≥ 50,000/µL on Day 8	For platelets <75,000 on day 8, hold cisplatin and do not make up dose.
Platelets < 50,000/µL on Day 8	For platelets <50,000 on day 8, hold cisplatin and do not make up dose and reduce cisplatin by one dose level for all subsequent cycles.

ANC ≥ 1000/μL on Day 8	None
ANC < 1000/μL but ≥ 500/μL on Day 8	For ANC <1000 but >=500/μL on day 8, hold cisplatin and do not make up dose.
ANC < 500/μL on Day 8	For ANC <500 on day 8, hold cisplatin and do not make up dose and reduce cisplatin by one dose level for all subsequent cycles.
* If >2 week delay is required at any time, discontinue the subject from protocol-mandated treatment and follow-up per protocol.	

**Hematological Toxicity Dose Reductions for Gemcitabine**	
**Labs**	**Action**
ANC ≥ 1,500/μL on Day 1	None
ANC <1500/μL on Day 1	For ANC <1500/μL on day 1, delay gemcitabine until the ANC ≥ 1500/μL. If the delay is one week or less, continue gemcitabine at the same dose and consider pegfilgrastim with subsequent cycles, if not receiving. If the delay is for more than a week* but ≤14 days and pegfilgrastim has not been used, include pegfilgrastim in all subsequent cycles. If there is a dose delay for more than a week due to neutropenia despite pegfilgrastim use, reduce gemcitabine by one dose level for this and all subsequent cycles and continue pegfilgrastim
ANC ≥ 1000/μL on Day 8	None
ANC < 1000/μL but ≥500/μL on Day 8	For ANC <1000 but >=500 on day 8, hold gemcitabine and do not make up dose.
ANC < 500/μL on Day 8	For ANC <500/μL on day 8, hold gemcitabine (do not make up dose) and reduce gemcitabine by one dose level for all subsequent cycles.
Platelets ≥100,000/μL on Day 1	None
Platelets < 100,000/μL on Day 1	For platelets <100,000/μL on day 1, delay gemcitabine until platelets ≥ 100,000/μL. If the delay is one week or less, continue gemcitabine at the same dose. If there is a dose delay for more than a week* but ≤14 days, reduce gemcitabine by one dose level for this and all subsequent cycles.
Platelets ≥ 75,000/μL on Day 8	None
Platelets < 75,000/μL but ≥ 50,000/μL on Day 8	For platelets < 75,000/μL but ≥ 50,000/μL on day 8, hold gemcitabine (do not make up dose).
Platelets < 50,000/μL on Day 8	For platelets <50,000 on day 8, hold gemcitabine (do not make up dose) and reduce gemcitabine by one dose level for all subsequent cycles.

*If >2 week delay is required, discontinue the subject from protocol-mandated treatment and follow-up per protocol.

4.4.2 Dose Modifications for GC for Non-Hematologic Toxicity

**Non-Hematological Toxicity Dose Reductions for Gemcitabine and Cisplatin**	
**Event**	**Action**
**Grade 4 Febrile Neutropenia** (defined as ANC <500/μL and temperature ≥ 38.3° C orally or a temperature of ≥ 38.0° C for more than 1 hour)	Add pegfilgrastim if not previously included, continuing GC at the same dose level. If pegfilgrastim had been used, then dose reduce **cisplatin** by one dose level for all subsequent cycles and continue pegfilgrastim.
**Kidney Dysfunction** (calculate CrCl on Day 1 and 8 of each cycle using CKD-Epi equation)	
CrCl ≥ 50ml/min	None.
CrCl < 50ml/min	Hold gemcitabine and cisplatin for up to 1 week if occurs on day 1. If the CrCl does not improve to ≥ 50ml/min in 1 week despite hydration and other supportive measures, discontinue the chemotherapy (all agents) and proceed to surgery. If it improves to ≥ 50ml/min within 1 week of scheduled day 1, proceed with gemcitabine and cisplatin administration. Hold gemcitabine and cisplatin and do not make up dose if occurs on day 8.
**Liver Dysfunction**	
Total bilirubin ≥ Grade 2 normal (or Grade 4 in patients with Gilbert's syndrome)	Hold gemcitabine and cisplatin until bilirubin is < Grade 2 (≤1.5 x ULN) (or <Grade 4 in patients with Gilbert's syndrome), then restart with a one dose level reduction in **gemcitabine** for this and all remaining cycles.
**Neurologic Toxicity: Neuropathy**	
Grade 1-2	None
Grade 3	Hold gemcitabine and cisplatin until resolves to Grade 1 or 2, then resume with a one dose level reduction in cisplatin. If occurs on day 8, hold gemcitabine and cisplatin and do not make up dose.
Grade 4	Remove from protocol directed treatment, and follow-up per protocol
**Gastrointestinal Toxicity: Vomiting**	
Grade 1-2	None
Grade 3 or 4 (despite maximal antiemetic medical intervention with aprepitant, corticosteroids, and 5HT-3 antagonists (e.g. ondansestron))	Proceed with one dose level reduction for next cycle in both cisplatin and gemcitabine. If grade 3 or 4 vomiting occurs despite dose reduction and maximal antiemetic therapy, remove from protocol directed treatment, and follow-up per protocol
**Other Non-specified Treatment-related Toxicity**	
Grade 2	None
Grade 3 or 4 (excluding alopecia and skin pigment changes)	Hold gemcitabine and cisplatin and monitor patient at least weekly. If toxicity resolves to grade 1 or completely resolves in ≤ 2 weeks, treatment can be resumed in the next cycle at the same dose levels for cisplatin and gemcitabine at the discretion of the treating provider and in consultation with the medical monitor. Any single delay in chemotherapy of more than 2 weeks or cumulative delays

	of more than 4 weeks will lead to removal from the protocol directed treatment and follow-up per protocol. If occurs on day 8, hold gemcitabine and cisplatin and do not make up dose.

4.4.3 Dose Modifications for Pembrolizumab for Events other than Infusion Reactions

Adverse events (both non-serious and serious) associated with pembrolizumab exposure may represent an immunologic etiology. These adverse events may occur shortly after the first dose or several months after the last dose of treatment. Pembrolizumab must be withheld for drug-related toxicities and severe or life-threatening AEs as per Table below. See Sections 7.1.4 and 7.3.

Dosing interruptions are permitted in the case of medical / surgical events or logistical reasons not related to study therapy (e.g., elective surgery, unrelated medical events, patient vacation, and/or holidays). Subjects should be placed back on study therapy within 2 weeks of the scheduled interruption, unless otherwise discussed with the Sponsor. The reason for interruption should be documented in the patient's study record.

Of note: Per the dose modification tables above, if administration of either cisplatin or gemcitabine on Day 1 is delayed, the other chemotherapy agent will also be held, and a new Day 1 will be scheduled. When that occurs, pembrolizumab should also be held until the new Day 1 based on gemcitabine/cisplatin is scheduled.

General instructions:

1. Corticosteroid taper should be initiated upon AE improving to Grade 1 or less and continue to taper over at least 4 weeks.
2. For situations where pembrolizumab has been withheld, pembrolizumab can be resumed after AE has been reduced to Grade 1 or 0 and corticosteroid has been tapered. Pembrolizumab should be permanently discontinued if AE does not resolve within 12 weeks of last dose or corticosteroids cannot be reduced to ≤10 mg prednisone or equivalent per day within 12 weeks.
3. For severe and life-threatening irAEs, IV corticosteroid should be initiated first followed by oral steroid. Other immunosuppressive treatment should be initiated if irAEs cannot be controlled by corticosteroids.

Immune-related AEs	Toxicity grade or conditions (CTCAEv4.0)	Action taken to pembrolizumab	irAE management with corticosteroid and/or other therapies	Monitor and follow-up
Pneumonitis	Grade 2	Withhold	• Administer corticosteroids (initial dose of 1-2 mg/kg prednisone or equivalent)	• Monitor participants for signs and symptoms of pneumonitis<br>• Evaluate participants with
	Grade 3 or 4, or recurrent Grade 2	Permanently discontinue		

			followed by taper	suspected pneumonitis with radiographic imaging and initiate corticosteroid treatment<br>• Add prophylactic antibiotics for opportunistic infections
Diarrhea / Colitis	Grade 2 or 3	Withhold	• Administer corticosteroids (initial dose of 1-2 mg/kg prednisone or equivalent) followed by taper	• Monitor participants for signs and symptoms of enterocolitis (ie, diarrhea, abdominal pain, blood or mucus in stool with or without fever) and of bowel perforation (ie, peritoneal signs and ileus).<br>• Participants with ≥ Grade 2 diarrhea suspecting colitis should consider GI consultation and performing endoscopy to rule out colitis.<br>• Participants with diarrhea/colitis should be advised to drink liberal quantities of clear fluids. If sufficient oral fluid intake is not feasible, fluid and electrolytes should be substituted via IV infusion.
	Grade 4	Permanently discontinue		
AST / ALT elevation or Increased bilirubin	Grade 2	Withhold	• Administer corticosteroids (initial dose of 0.5- 1 mg/kg prednisone or equivalent)	• Monitor with liver function tests (consider weekly or more frequently until liver enzyme

			followed by taper	value returned to baseline or is stable
	Grade 3 or 4	Permanently discontinue	• Administer corticosteroids (initial dose of 1-2 mg/kg prednisone or equivalent) followed by taper	
Type 1 diabetes mellitus (T1DM) or Hyperglycemia	Newly onset T1DM or Grade 3 or 4 hyperglycemia associated with evidence of β-cell failure	Withhold	• Initiate insulin replacement therapy for participants with T1DM<br>• Administer anti-hyperglycemic in participants with hyperglycemia	• Monitor participants for hyperglycemia or other signs and symptoms of diabetes.
Hypophysitis	Grade 2	Withhold	• Administer corticosteroids and initiate hormonal replacements as clinically indicated.	• Monitor for signs and symptoms of hypophysitis (including hypopituitarism and adrenal insufficiency)
	Grade 3 or 4	Withhold or permanently discontinue[1]		
Hyperthyroidism	Grade 2	Continue	• Treat with non-selective beta-blockers (eg, propranolol) or thionamides as appropriate	• Monitor for signs and symptoms of thyroid disorders.
	Grade 3 or 4	Withhold or permanently discontinue[1]		
Hypothyroidism	Grade 2-4	Continue	• Initiate thyroid replacement hormones (eg, levothyroxine or liothyroinine) per standard of care	• Monitor for signs and symptoms of thyroid disorders.
Nephritis and Renal dysfunction	Grade 2	Withhold	• Administer corticosteroids (prednisone 1-2 mg/kg or equivalent) followed by taper.	• Monitor changes of renal function
	Grade 3 or 4	Permanently discontinue		
Myocarditis	Grade 1 or 2	Withhold	• Based on severity of AE	• Ensure adequate evaluation to

	Grade 3 or 4	Permanently discontinue	administer corticosteroids	confirm etiology and/or exclude other causes
All other immune-related AEs	Intolerable/ persistent Grade 2	Withhold	• Based on type and severity of AE administer corticosteroids	• Ensure adequate evaluation to confirm etiology and/or exclude other causes
	Grade 3	Withhold or discontinue based on the type of event. Events that require discontinuation include and not limited to: Gullain-Barre Syndrome, encephalitis		
	Grade 4 or recurrent Grade 3	Permanently discontinue		

1. Withhold or permanently discontinue pembrolizumab is at the discretion of the investigator or treating physician.

NOTE:
For participants with Grade 3 or 4 immune-related endocrinopathy where withhold of pembrolizumab is required, pembrolizumab may be resumed when AE resolves to ≤ Grade 2 and is controlled with hormonal replacement therapy or achieved metabolic control (in case of T1DM).

Dosing interruptions are permitted in the case of medical / surgical events or logistical reasons not related to study therapy (e.g., elective surgery, unrelated medical events, patient vacation, and/or holidays). Subjects should be placed back on study therapy within 2 weeks of the scheduled interruption, unless otherwise discussed with the Sponsor. The reason for interruption should be documented in the patient's study record.

4.4.4 Rescue Medications and Supportive Care Guidelines

Subjects should receive appropriate supportive care measures as deemed necessary by the treating investigator. Suggested supportive care measures for the management of adverse events with potential immunologic etiology are outlined below. Where appropriate, these guidelines include the use of oral or intravenous treatment with corticosteroids as well as additional anti-inflammatory agents if symptoms do not improve with administration of corticosteroids. Note that several courses of steroid tapering may be necessary as symptoms may worsen when the steroid dose is decreased. For each disorder, attempts should be made to rule out other causes such as metastatic disease or bacterial or viral infection, which might require additional supportive care. The treatment guidelines are intended to be applied when the investigator determines the events to be related to pembrolizumab.

Note: if after the evaluation the event is determined not to be related, the investigator does not need to follow the treatment guidance (as outlined below).

It may be necessary to perform conditional procedures such as bronchoscopy, endoscopy, or skin photography as part of evaluation of the event.

<u>Pneumonitis:</u>

- For Grade 2 events, treat with systemic corticosteroids. When symptoms improve to Grade 1 or less, steroid taper should be started and continued over no less than 4 weeks.
- For Grade 3-4 events, immediately treat with intravenous steroids. Administer additional anti-inflammatory measures, as needed.
- Add prophylactic antibiotics for opportunistic infections in the case of prolonged steroid administration.

<u>Diarrhea/Colitis:</u>

Subjects should be carefully monitored for signs and symptoms of enterocolitis (such as diarrhea, abdominal pain, blood or mucus in stool, with or without fever) and of bowel perforation (such as peritoneal signs and ileus).

- All subjects who experience diarrhea/colitis should be advised to drink liberal quantities of clear fluids. If sufficient oral fluid intake is not feasible, fluid and electrolytes should be substituted via IV infusion. For Grade 2 or higher diarrhea, consider GI consultation and endoscopy to confirm or rule out colitis.
- For Grade 2 diarrhea/colitis, administer oral corticosteroids.
- For Grade 3 or 4 diarrhea/colitis, treat with intravenous steroids followed by high dose oral steroids.
- When symptoms improve to Grade 1 or less, steroid taper should be started and continued over no less than 4 weeks.

<u>Type 1 diabetes mellitus:</u> (if new onset, including diabetic ketoacidosis [DKA]) or ≥ Grade 3 Hyperglycemia, if associated with ketosis (ketonuria) or metabolic acidosis (DKA)

- For T1DM or Grade 3-4 Hyperglycemia
  - Insulin replacement therapy is recommended for Type I diabetes mellitus and for Grade 3-4 hyperglycemia associated with metabolic acidosis or ketonuria.
  - Evaluate patients with serum glucose and a metabolic panel, urine ketones, glycosylated hemoglobin, and C-peptide.

<u>Hypophysitis:</u>

- For Grade 2 events, treat with corticosteroids. When symptoms improve to Grade 1 or less, steroid taper should be started and continued over no less than 4 weeks. Replacement of appropriate hormones may be required as the steroid dose is tapered.
- For Grade 3-4 events, treat with an initial dose of IV corticosteroids followed by oral corticosteroids. When symptoms improve to Grade 1 or less, steroid taper should be started and continued over no less than 4 weeks. Replacement of appropriate hormones may be required as the steroid dose is tapered.

Hyperthyroidism or Hypothyroidism:
Thyroid disorders can occur at any time during treatment. Monitor patients for changes in thyroid function (at the start of treatment, periodically during treatment, and as indicated based on clinical evaluation) and for clinical signs and symptoms of thyroid disorders

- Grade 2 hyperthyroidism events (and Grade 3-4 hypothyroidism):
  - In hyperthyroidism, non-selective beta-blockers (e.g. propranolol) are suggested as initial therapy.
  - In hypothyroidism, thyroid hormone replacement therapy, with levothyroxine or liothyroinine, is indicated per standard of care.
- Grade 3-4 hyperthyroidism
  - Treat with an initial dose of IV corticosteroid followed by oral corticosteroids. When symptoms improve to Grade 1 or less, steroid taper should be started and continued over no less than 4 weeks. Replacement of appropriate hormones may be required as the steroid dose is tapered.

Hepatic:
- For Grade 2 events, monitor liver function tests more frequently until returned to baseline values (consider weekly).
  - Treat with IV or oral corticosteroids
- For Grade 3-4 events, treat with intravenous corticosteroids for 24 to 48 hours.
- When symptoms improve to Grade 1 or less, a steroid taper should be started and continued over no less than 4 weeks.

Renal Failure or Nephritis:
- For **Grade 2** events, treat with corticosteroids.
- For **Grade 3-4** events, treat with systemic corticosteroids.

When symptoms improve to Grade 1 or less, steroid taper should be started and continued over no less than 4 weeks.

4.5 Concomitant Medications/Vaccinations (allowed & prohibited)

Medications or vaccinations specifically prohibited in the exclusion criteria are not allowed during the ongoing trial. If there is a clinical indication for one of

these or other medications or vaccinations specifically prohibited during the trial, discontinuation from trial therapy or vaccination may be required. The investigator should discuss any questions regarding this with the Merck Clinical team. The final decision on any supportive therapy or vaccination rests with the investigator and/or the subject's primary physician. An exception to this is corticosteroids that are allowed to be administered as antiemetic prophylaxis. In addition, topical and inhaled corticosteroids are allowed. Vaccinations for flu that are not live vaccines are allowed.

4.5.1 Acceptable Concomitant Medications

All treatments that the investigator considers necessary for a subject's welfare may be administered at the discretion of the investigator in keeping with the community standards of medical care. All concomitant medication will be recorded on the case report form (CRF) including all prescription, over-the-counter (OTC), herbal supplements, and IV medications and fluids. If changes occur during the trial period, documentation of drug dosage, frequency, route, and date may also be included on the CRF.

All concomitant medications received within 28 days before the first dose of trial treatment and 30 days after the last dose of trial treatment should be recorded. Concomitant medications administered after 30 days after the last dose of trial treatment should be recorded for SAEs and ECIs as defined in Section 7.3.3.

4.5.2 Prohibited Concomitant Medications

Subjects are prohibited from receiving the following therapies during the Screening and Treatment Phase of this trial:

- Anti-neoplastic systemic chemotherapy or biological therapy
- Immunotherapy not specified in this protocol
- Chemotherapy not specified in this protocol
- Investigational agents other than pembrolizumab
- Live vaccines within 30 days prior to the first dose of trial treatment and while participating in the trial. Examples of live vaccines include, but are not limited to, the following: measles, mumps, rubella, chicken pox, yellow fever, rabies, BCG, typhoid (oral) vaccine and FluMist®.
- Glucocorticoids for any purpose other than to modulate symptoms from an event of suspected immunologic etiology or for antiemetic prophylaxis. The use of physiologic doses of corticosteroids may be approved after consultation with the Sponsor.

Subjects who, in the assessment by the investigator, require the use of any of the aforementioned treatments for clinical management should be removed from the trial and followed up per protocol. Subjects may receive other medications that the investigator deems to be medically necessary.

The Exclusion Criteria describes other medications which are prohibited in this trial.

There are no prohibited therapies during the Post-Treatment Follow-up Phase.

4.5.3 Diet

Subjects should maintain a normal diet unless modifications are required to manage an AE such as diarrhea, nausea or vomiting.

4.6 Duration of Therapy

In the absence of treatment delays due to AEs, treatment may continue until the end of 4 cycles or until:

- Inter-current illness that prevents further administration of treatment
- Unacceptable toxicity
- Pregnancy
- Patient decides to withdraw from study treatment, OR
- General or specific changes in the patient's condition render the patient unacceptable for further treatment in the judgment of the investigator.
- Inability to complete protocol chemotherapy due to the following reasons:
  - A continuous treatment delay for any reason >14 days at any time after starting chemotherapy, a cumulative delay in chemotherapy of 28 days total, or if CrCl<50mL/min persists for one week despite best supportive care and holding the chemotherapy
  - The need for more than one dose level reduction for cisplatin or more than two dose level reductions for gemcitabine
  - Patient is no longer a candidate for cystectomy in the opinion of the physician

A subject must be discontinued from the study treatment for any of the following reasons:

- Evidence of radiographic disease progression
- Unacceptable adverse experiences as described in Section 7.0
- Investigator's decision to withdraw the subject
- Noncompliance with trial treatment or procedure requirements
- The subject is lost to follow-up
- Administrative reasons

.

4.7 Duration of Follow Up

After the end of treatment, each subject will be followed for 30 days for adverse event monitoring (serious adverse events and Events of Clinical Interest will be collected for 90 days after the end of treatment as described in section 7.3).

Patients will otherwise be followed from enrollment for up to 5 years or until death, whichever comes first.

4.8 Removal of Patients from Protocol Therapy

Patients will be removed from protocol therapy and the site PI notified when any of the criteria listed in section 4.6 apply. The reason for discontinuation of protocol therapy will be documented on the eCRF.

In case a patient decides to prematurely discontinue protocol therapy ("refuses treatment"), the patient should be asked if she or he may still be contacted for further scheduled study assessments. The outcome of that discussion should be documented in both the medical records and in the eCRF.

Excessive patient withdrawals from protocol therapy or from the study can render the study un-interpretable; therefore, unnecessary withdrawal of patients should be avoided.

4.9 Study Withdrawal

If a patient decides to withdraw from the study (and not just from protocol therapy) an effort should be made to complete and report study assessments as thoroughly as possible. At the time of withdrawal, the investigator should attempt to establish as completely as possible the reason for the study withdrawal.

- The patient should be asked if they are willing to allow for the abstraction of relevant information from their medical record in order to meet the long term follow up (e.g., survival) objectives outlined in the protocol.
- A complete final evaluation at the time of the patient's study withdrawal should be obtained with an explanation of why the patient is withdrawing from the study.
- If the patient is noncompliant and does not return for an end of study follow up assessment, this should be documented in the eCRF.
- If the reason for removal of a patient from the study is an adverse event, the principal specific event will be recorded on the eCRF.

Excessive patient withdrawals from protocol therapy or from the study can render the study un-interpretable; therefore, unnecessary withdrawal of patients should be avoided.

4.10 Clinical Criteria for Early Trial Termination

Early trial termination will be the result of the criteria specified below:

1. Quality or quantity of data recording is inaccurate or incomplete.
2. Poor adherence to protocol and regulatory requirements.
3. Incidence or severity of adverse drug reaction in this or other studies indicates a potential health hazard to subjects.

4. Plans to modify or discontinue the development of the study drug.

In the event of Merck decision to no longer supply study drug, ample notification will be provided so that appropriate adjustments to subject treatment can be made.

5.0 DRUG INFORMATION

The investigator shall take responsibility for and shall take all steps to maintain appropriate records and ensure appropriate supply, storage, handling, distribution and usage of investigational product in accordance with the protocol and any applicable laws and regulations.

5.1 Pembrolizumab

5.1.1 Description

Pembrolizumab (MK-3475) solution for infusion is a sterile, non-pyrogenic aqueous solution supplied in single-use type I glass vial containing 100 mg/4 mL of pembrolizumab (manufactured using the fully formulated drug substance with L-histidine as a buffering agent, polysorbate 80 as a surfactant, and sucrose as a stabilizer/tonicity modifier). The product is preservative-free solution which is essentially free of extraneous particulates.

5.1.2 Supplier/How Supplied/Packaging and Labeling Information

Pembrolizumab will be provided at no cost to the study patient by Merck, the manufacturer of the drug. Clinical supplies will be affixed with a clinical label in accordance with regulatory requirements.

This trial is open-label; therefore, the subject, the trial site personnel, the Sponsor and/or designee are not blinded to treatment. Drug identity (name, strength) is included in the label text; random code/disclosure envelopes or lists are not provided.

5.1.3 Storage and Handling Requirements and Dispensing

Clinical supplies must be stored in a secure, limited-access location under refrigerated conditions (2°C to 8°C).
Receipt and dispensing of trial medication must be recorded by an authorized person at the trial site.
Clinical supplies may not be used for any purpose other than that stated in the protocol.

5.1.4 Preparation of Infusion Solution

Aseptic technique must be strictly observed throughout the preparation procedure preferably in a biologic safety cabinet or hood since no anti-microbial preservative is present in the solutions.
Equilibrate required number of pembrolizumab (MK-3475) vials to room temperature.
The preferred method of dose preparation is the volumetric method, gravimetric method is not permitted.
Choose a suitable infusion bag size so that the following conditions are met:

- Concentration of pembrolizumab MK-3475 is between 1 mg/mL and 10 mg/mL
- The infusion volume to bag capacity ratio should not be less than 0.3. In other words, the bag must be filled to at least 30% of its capacity.

Choose a suitable infusion bag material. The bag may be empty, or it may contain normal saline. The following infusion bag materials are compatible with pembrolizumab:

- PVC plasticized with DEHP
- Non-PVC (polyolefin)
- EVA
- PE lined polyolefin

*Contact Sponsor for materials not listed above

Calculate the volume of pembrolizumab and normal saline required to prepare the infusion (admixture) bag

Volume of pembrolizumab (mL) = required dose amount (mg) / 25 (mg/mL)

Volume of normal saline = total infusion volume – volume of pembrolizumab from above

If a bag pre-filled with normal saline is being used, remove the excess volume of normal saline using a sterile syringe (Polypropylene, latex-free) attached to a suitable needle. Keep in consideration the excess bag fill volume as well as the volume of reconstituted pembrolizumab to be added to the bag to prepare the infusion solution.
If an empty bag is being used, withdraw the necessary volume of normal saline from another appropriate bag and inject into the empty bag. Keep in consideration the volume of reconstituted pembrolizumab to be added to the bag to prepare the infusion solution.

Withdraw the required volume of pembrolizumab from the vial(s) (up to 4 mL from each vial) using a sterile syringe attached to a suitable needle. The vial(s) may need to be inverted to remove solution.

Volume of pembrolizumab (mL) = required dose amount (mg) / 25 (mg/mL)

Note: If it is necessary to use several vials, it is advisable to withdraw from several vials into a suitable size single use syringe using a new needle for each vial.

Add the required pembrolizumab into the infusion IV bag containing normal saline and gently invert the bag 10-15 times to mix the solution.

Pembrolizumab solutions may be stored at room temperature for a cumulative time of up to 4 hours. This includes room temperature storage of admixture solutions in the IV bags and the duration of infusion.

In addition, IV bags may be stored under refrigeration at 2 °C to 8 °C (36 °F to 46 °F) for up to 20 hours. If refrigerated, allow the IV bags to come to room temperature prior to use.
Do not freeze the pembrolizumab infusion solution.
Discard any unused portion left in the vial as the product contains no preservative.

5.1.5 Method of Administration

Pembrolizumab (MK-3475) infusions should be administered in 30 minutes, with a window of -5 and +10 minutes, using an infusion pump. A central catheter is not required for infusion; however, if a subject has a central venous catheter in place, it is recommended that it be used for the infusion. The following infusion set materials are compatible with (pembrolizumab) MK-3475:

- PVC and tri-(2-ethylhexyl) trimellitate (TOTM) infusion set
- Polyethylene lined PVC infusion set
- PVC Infusion set that is plasticized using Di-2-ethylhexyl Terephthalate (DEHT)
- Polyurethane set

A sterile, non-pyrogenic, low-protein binding 0.2 to 5 µm in-line filter made of polyethersulfone (PES) must be used during administration to remove any adventitious particles. If the infusion set does not contain 0.2 to 5 µm in-line filter, it is recommended to use 0.2 to 5 µm add-on filter which may contain an extension line (Note: the materials of the extension line and filter should be as mentioned above).
Attach the infusion line to the pump and prime the line, either with normal saline (at least 25 mL) or with infusion solution as per local SOP, before starting the infusion.
Infuse pembrolizumab (MK-3475) over approximately 30 minutes, with a window of -5 and +10 minutes, through a peripheral line or indwelling catheter.
Ensure the entire contents of the bag are dosed and all remaining drug solution in the line is administered according to institutional guidelines for saline flushing.
Document volume administered according to data entry guidelines.

In case of infusion reactions, infusion rate may differ; refer to protocol for specific instructions.

Whenever possible, the lowest infusion rate should be used that will allow completion of the infusion within the 30 minutes. Maximum rate of infusion should not exceed 6.7 mL/min. through a peripheral line or indwelling catheter. However, when it is necessary to infuse a larger volume (i.e. 250 mL), the flow rate may go as high as 10 mL/min (maximum) in order to keep the infusion within the window as defined above.

Do not co-administer other drugs through the same infusion line.

Unused infusion solution for injection should not be used for another infusion of the same subject or different subject.

5.1.6 Storage and Stability

Pembrolizumab (MK-3475) Solution for Infusion, 100 mg/ 4 mL vial: pembrolizumab (MK-3475) Solution for Infusion vials should be stored at refrigerated conditions (2 – 8 °C) and protected from light.

Note: Vials should be stored in the original box to ensure the drug product is protected from light.

Pembrolizumab (MK-3475) infusion solutions should be prepared in 0.9% Sodium Chloride Injection, USP (normal saline) or regional equivalent or 5% Dextrose Injection, USP (5% dextrose) or regional equivalent and the final concentration of pembrolizumab (MK-3475) in the infusion solutions should be between 1 mg/mL and 10 mg/mL.
Please note, the preferred diluent is 0.9% sodium chloride and 5% dextrose is only permissible if normal saline is not available.
Pembrolizumab (MK-3475) should not be mixed with other diluents.
Pembrolizumab (MK-3475) solutions may be stored at room temperature for a cumulative time of up to 4 hours. This includes room temperature storage of admixture solutions in the IV bags and the duration of infusion
In addition, IV bags may be stored under refrigeration at 2°C to 8°C (36°F to 46°F) for up to a cumulative time of 20 hours. If refrigerated, allow the IV bags to come to room temperature prior to use.
Parenteral drug products should be inspected visually for particulate matter and discoloration prior to administration. Discard the drug product vial if extraneous particulate matter other than translucent to white proteinaceous particles is observed.
Sites should follow their SOPs for drug transport and delivery, with all possible effort to minimize agitation of the drug product between the pharmacy and the clinic.
Do not use pembrolizumab (MK-3475) if discoloration is observed.
Do not shake or freeze the vial(s).
Do not administer the product as an intravenous (IV) push or bolus.
Do not combine, dilute or administer it as an infusion with other medicinal products.
Any deviation from this guidance must be discussed with sponsor.

5.1.7 Return and Retention

The investigator is responsible for keeping accurate records of the clinical supplies received from Merck or designee, the amount dispensed to and returned by the subjects and the amount remaining at the conclusion of the trial.

Upon completion or termination of the study, all unused and/or partially used investigational product will be destroyed at the site per UNC IDS drug destruction policy. It is the investigator's responsibility to arrange for disposal of all empty containers, provided that procedures for proper disposal have been established according to applicable federal, state, local and institutional guidelines and procedures, and provided that appropriate records of disposal are kept.

5.1.8 Drug Ordering and Accountability

Pembrolizumab should be ordered from Merck by completing and emailing Drug Request Form provided to each site.

5.1.9 Adverse Events Associated with Pembrolizumab

The most common adverse reactions (reported in ≥20% of subjects in clinical trials of pembrolizumab) included fatigue, cough, nausea, pruritus, rash, decreased appetite, constipation, arthralgia, and diarrhea. The following warnings are associated with the use of pembrolizumab:

Immune-Mediated Pneumonitis
Pneumonitis occurred in ~3% of melanoma patients treated in clinical trials of pembrolizumab. The median time to development of pneumonitis was 5 months with a median duration of 4.9 months. The one patient with Grade 3 pneumonitis required initial treatment with high-dose systemic corticosteroids (greater than or equal to 40 mg prednisone or equivalent per day) followed by a corticosteroid taper. Pneumonitis completely resolved in seven of the nine patients with Grade 2-3 pneumonitis.

Immune-Mediated Colitis
Colitis (including microscopic colitis) occurred 1% of melanoma patients treated in clinical trials of pembrolizumab. The median time to onset of was 6.5 months with a median duration of 2.6 months. All three patients with Grade 2 or 3 colitis were treated with high-dose corticosteroids (greater than or equal to 40 mg prednisone or equivalent per day).

Immune-Mediated Hepatitis
Hepatitis (including autoimmune hepatitis) occurred in 0.5% of melanoma patients treated in clinical trials of pembrolizumab. The time to onset was 22 days for the case of Grade 4 hepatitis, which lasted 1.1 months. The patient with Grade 4 hepatitis permanently discontinued pembrolizumab and was treated with high-dose (greater than or equal to 40 mg prednisone or equivalent per day) systemic corticosteroids followed by a corticosteroid taper. Both patients with hepatitis experienced complete resolution of the event.

Immune-Mediated Hypophysitis
Hypophysitis occurred in 0.5% of melanoma patients treated in clinical trials of pembrolizumab. The time to onset was 1.7 months for the patient with Grade 4

hypophysitis and 1.3 months for the patient with Grade 2 hypophysitis. Both patients were treated with high-dose (greater than or equal to 40 mg prednisone or equivalent per day) corticosteroids followed by a corticosteroid taper and remained on a physiologic replacement dose.

Renal Failure and Immune-Mediated Nephritis

Nephritis occurred in 3 (0.7%) patients of melanoma patients treated in clinical trials of pembrolizumab, consisting of one case of Grade 2 autoimmune nephritis (0.2%) and two cases of interstitial nephritis with renal failure (0.5%), one Grade 3 and one Grade 4. The time to onset of autoimmune nephritis was 11.6 months after the first dose of pembrolizumab (5 months after the last dose) and lasted 3.2 months; this patient did not have a biopsy. Acute interstitial nephritis was confirmed by renal biopsy in two patients with Grades 3-4 renal failure. All three patients fully recovered their renal function with treatment with high-dose corticosteroids (greater than or equal to 40 mg prednisone or equivalent per day) followed by a corticosteroid taper.

Immune-Mediated Hyperthyroidism

Hyperthyroidism occurred in 5 (1.2%) of 411 melanoma patients treated in clinical trials of pembrolizumab. The median time to onset was 1.5 months and the median duration was 2.8 months (range 0.9 to 6.1). One of two patients with Grade 2 and the one patient with Grade 3 hyperthyroidism required initial treatment with high-dose corticosteroids (greater than or equal to 40 mg prednisone or equivalent per day) followed by a corticosteroid taper. One patient (0.2%) required permanent discontinuation of pembrolizumab due to hyperthyroidism. All five patients with hyperthyroidism experienced complete resolution of the event.

Immune-Mediated Hypothyroidism

Hypothyroidism occurred in 34 (8.3%) of 411 melanoma patients treated in clinical trials of pembrolizumab. The median time to onset of hypothyroidism was 3.5 months. All but two of the patients with hypothyroidism were treated with long-term thyroid hormone replacement therapy. The other two patients only required short-term thyroid hormone replacement therapy. No patient received corticosteroids or discontinued pembrolizumab for management of hypothyroidism. Thyroid disorders can occur at any time during treatment.

Other Immune-Mediated Adverse Reactions

Other clinically important immune-mediated adverse reactions can occur. The following clinically significant, immune-mediated adverse reactions occurred in less than 1% of patients treated with pembrolizumab, including exfoliative dermatitis, uveitis, arthritis, myositis, pancreatitis, hemolytic anemia, partial seizures arising in a patient with inflammatory foci in brain parenchyma, and adrenal insufficiency.

Across clinical studies with pembrolizumab in approximately 2,000 patients, the following additional clinically significant, immune-mediated adverse reactions were reported in less than 1% of patients: myasthenic syndrome, optic neuritis, and rhabdomyolysis.

Embryofetal Toxicity

Based on its mechanism of action, pembrolizumab may cause fetal harm when administered to a pregnant woman. Animal models link the PD-1/PD-L1 signaling pathway with maintenance of pregnancy through induction of maternal immune tolerance to fetal tissue.

Stevens-Johnson Syndrome (SJS) and Toxic Epidermal Necrolysis (TEN)

The risk of SJS and TEN is reported at approximately 0.4 – 7 cases per million patient years in the general adult population. Independent risk factors include certain medications such as anticonvulsants, sulfonamides, aminopenicillins, allopurinol, and NSAIDs. Non-medication triggers include infection, contrast media, and vaccinations. Malignancy is associated with an increased mortality rate in patients with SJS and TEN.

Myocarditis

A total of 6 cases of myocarditis have been reported in patients treated with pembrolizumab in clinical trials in an expanded access program. There was one fatal case reported in a clinical trial. Immune-mediated myocarditis should be suspected if other causes of myocarditis such as infection or prior radiation therapy have been excluded. Risk factors include certain medications and treatment modalities such as radiation, anthracycline, alkylating agents and most recently checkpoint inhibitors.

5.1.10 Contraindications

There are no reported contraindications associated with the use of pembrolizumab.

Pembrolizumab may have adverse effects on a fetus in utero. Furthermore, it is not known if pembrolizumab has transient adverse effects on the composition of sperm.

5.1.11 Contraception

Pembrolizumab may have adverse effects on a fetus in utero. Furthermore, it is not known if pembrolizumab has transient adverse effects on the composition of sperm.

For this trial, male subjects will be considered to be of non-reproductive potential if they have azoospermia (whether due to having had a vasectomy or due to an underlying medical condition).

Female subjects will be considered of non-reproductive potential if they are either:

(1) postmenopausal (defined as at least 12 months with no menses without an alternative medical cause; in women < 45 years of age a high follicle stimulating hormone (FSH) level in the postmenopausal range may be used to confirm a post-menopausal state in women not using hormonal contraception or hormonal replacement therapy. In the absence of 12 months of amenorrhea, a single FSH measurement is insufficient.);

OR

(2) have had a hysterectomy and/or bilateral oophorectomy, bilateral salpingectomy or bilateral tubal ligation/occlusion, at least 6 weeks prior to screening;

OR

(3) has a congenital or acquired condition that prevents childbearing.

Female and male subjects of reproductive potential must agree to avoid becoming pregnant or impregnating a partner, respectively, while receiving study drug and for 120 days after the last dose of study drug by complying with one of the following:

(1) practice abstinence† from heterosexual activity;

OR

(2) use (or have their partner use) acceptable contraception during heterosexual activity.

Acceptable methods of contraception are‡:

Single method (one of the following is acceptable):

- intrauterine device (IUD)
- vasectomy of a female subject's male partner
- contraceptive rod implanted into the skin

Combination method (requires use of two of the following):

- diaphragm with spermicide (cannot be used in conjunction with cervical cap/spermicide)
- cervical cap with spermicide (nulliparous women only)

- contraceptive sponge (nulliparous women only)
- male condom or female condom (cannot be used together)
- hormonal contraceptive: oral contraceptive pill (estrogen/progestin pill or progestin-only pill), contraceptive skin patch, vaginal contraceptive ring, or subcutaneous contraceptive injection

†Abstinence (relative to heterosexual activity) can be used as the sole method of contraception if it is consistently employed as the subject's preferred and usual lifestyle and if considered acceptable by local regulatory agencies and ERCs/IRBs. Periodic abstinence (e.g., calendar, ovulation, sympto-thermal, post-ovulation methods, etc.) and withdrawal are not acceptable methods of contraception.

‡If a contraceptive method listed above is restricted by local regulations/guidelines, then it does not qualify as an acceptable method of contraception for subjects participating at sites in this country/region.

Subjects should be informed that taking the study medication may involve unknown risks to the fetus (unborn baby) if pregnancy were to occur during the study. In order to participate in the study subjects of childbearing potential must adhere to the contraception requirement (described above) from the day of study medication initiation (or 14 days prior to the initiation of study medication for oral contraception) throughout the study period up to 120 days after the last dose of trial therapy. If there is any question that a subject of childbearing potential will not reliably comply with the requirements for contraception, that subject should not be entered into the study.

5.1.12 Use in Pregnancy

Risk Summary

Based on its mechanism of action, pembrolizumab can cause fetal harm when administered to a pregnant woman. In animal models, the PD-1/PD-L1 signaling pathway is important in the maintenance of pregnancy through induction of maternal immune tolerance to fetal tissue. Human IgG4 (immunoglobulins) are known to cross the placenta; therefore, pembrolizumab has the potential to be transmitted from the mother to the developing fetus. There are no available human data informing the risk of embryo-fetal toxicity. Apprise pregnant women of the potential risk to a fetus.

In the U.S. general population, the estimated background risk of major birth defects and miscarriage in clinically recognized pregnancies is 2-4% and 15-20%, respectively.

If a subject inadvertently becomes pregnant while on treatment with pembrolizumab, the subject will immediately be removed from the study. The site will contact the subject at least monthly and document the subject's status until the pregnancy has been completed or terminated. The outcome of the pregnancy will be reported to the Sponsor and to Merck without delay and within 24 hours if the outcome is a serious adverse experience (e.g., death, abortion, congenital anomaly, or other disabling or life-threatening complication to the mother or newborn).

The study investigator will make every effort to obtain permission to follow the outcome of the pregnancy and report the condition of the fetus or newborn to the Sponsor. If a male subject impregnates his female partner the study personnel at the site must be informed immediately and the pregnancy reported to the Sponsor and to Merck and followed as described above and in Section 7.3.3.

5.1.13 Use in Nursing Women

It is not known whether pembrolizumab is excreted in human milk. No studies have been conducted to assess the impact of pembrolizumab on milk production or its presence in breast milk. Because many drugs are excreted in human milk, instruct women to discontinue nursing during treatment with pembrolizumab and for 4 months after the final dose.

5.1.14 Overdose

For purposes of this trial, an overdose of pembrolizumab will be defined as any dose of 1,000 mg or greater (≥5 times the indicated dose). No specific information is available on the treatment of overdose of pembrolizumab. Appropriate supportive treatment should be provided if clinically indicated. In the event of overdose, the subject should be observed closely for signs of toxicity. Appropriate supportive treatment should be provided if clinically indicated.

If an adverse event(s) is associated with ("results from") the overdose of a Merck product, the adverse event(s) is reported as a serious adverse event, even if no other seriousness criteria are met.

If a dose of Merck's product meeting the protocol definition of overdose is taken without any associated clinical symptoms or abnormal laboratory results, the overdose is reported as a non-serious Event of Clinical Interest (ECI), using the terminology "accidental or intentional overdose without adverse effect."
All reports of overdose with and without an adverse event must be reported within 24 hours to the Sponsor and within 2 working days hours to Merck Global Safety. (Attn: Worldwide Product Safety; Fax: 215 993-1220).

5.2 Gemcitabine Hydrochloride

Mechanism of Action: Gemcitabine (2'-Deoxy-2', 2'-difluorocytidine monohydrochloride) is a nucleoside analog of deoxycytidine. This antimetabolite,

a pyrimidine analog inhibiting both DNA and RNA viruses, is cell-cycle-specific in blocking the cells at the G1/S and is retained in human tumor cells for long periods. Studies suggest that gemcitabine is activated by deoxycytidine kinase. Deoxycytidine has been shown to reverse the growth inhibitory activity of gemcitabine.

Metabolism and Elimination: Gemcitabine is metabolized by the liver to form the inactive uracil derivative, 2'-deoxy-2',2'-difluorouridine (dFdU). The inactive metabolite does not appear to accumulate with weekly dosing; however, it is excreted by the kidneys and may accumulate in patients with decreased renal function.

Dosing, Administration, Preparation, Storage, and Stability: See Section 4.2 for dosing information. Refer to the current FDA-approved package insert for drug administration, preparation, storage, stability and special handling information.

How Supplied: Gemcitabine is commercially available and will not be supplied free of charge to the patient. Refer to the current FDA-approved package insert.

Adverse Events: Adverse effects reported in 10% or more of patients receiving gemcitabine monotherapy include myelosuppression (i.e., anemia, leukopenia, neutropenia, thrombocytopenia), proteinuria, hematuria, increased BUN, nausea, vomiting, pain, fever, rash, pruritus, dyspnea, constipation, diarrhea, hemorrhage, peripheral edema, edema, flu-like symptoms, infection, alopecia, stomatitis, somnolence, paresthesias, and increased serum AST, ALT, alkaline phosphatase, and bilirubin concentrations.

Dose limiting toxicity is bone marrow suppression with mild to moderate granulocytopenia, anemia and thrombocytopenia. There has been no evidence of cumulative WBC or platelet toxicity. Gastrointestinal toxicities include nausea, vomiting, and diarrhea. Gemcitabine should be used with caution in patients with impaired liver function since abnormalities of liver transaminase enzymes have been reported. Mild proteinuria and hematuria have been reported but were not clinically significant and usually not associated with any change in serum creatinine or BUN. A few cases of renal failure of uncertain etiology have been reported. Gemcitabine should be used with caution in patients with impaired renal function. Toxicities associated with allergic reaction include rash, pruritus, desquamation, vesiculation, ulceration, and dyspnea. Bronchospasm has been reported in less than 1% of patients. Twenty percent of patients have also experienced flu-like symptoms such as fever, headache, back pain, chills, myalgia, asthenia, anorexia, cough, rhinitis, malaise, sweating, and insomnia. Other toxicities include edema or peripheral edema in 30% of patients, alopecia, somnolence, diarrhea, constipation, and oral toxicity (soreness and erythema). Pulmonary edema has been a rare occurrence (less than 1%). A few cases of hypotension have been reported, as well as myocardial infarction, congestive

heart failure and arrhythmia. However, there is no clear evidence that gemcitabine causes cardiac toxicity.

Refer to the current FDA-approved package insert for the most comprehensive and up to date information on adverse reactions.

Pregnancy and Lactation: Category D. Gemcitabine may cause fetal harm when administered to a pregnant woman. This agent has produced teratogenic effects in mice and rabbits when administered at a dose of < 2 mg/m2. Adverse effects included decreased fetal viability, weight and morphologic defects. There is no data on gemcitabine administration during human pregnancy, and it is not currently known if metabolites are excreted in human milk. However, many drugs are excreted in human milk, and there is a potential for adverse effects in nursing infants. Therefore, the use of gemcitabine should be avoided in pregnant or nursing women because of the potential hazard to the fetus or infant.

Drug Interactions: Per gemcitabine package insert, no formal drug interaction studies have been performed to date. When gemcitabine was administered with carboplatin or paclitaxel there was minimal or no effect on the pharmacokinetics of the studied drugs.

5.3 Cisplatin (CDDP)

Mechanism of Action: Cisplatin (cis-diamminedichloroplatinum) is a heavy metal complex containing a central platinum atom surrounded by two chloride atoms and two ammonia molecules in the cis position. It is water soluble and acts as a bifunctional alkylating agent with cell cycle nonspecific characteristics. The intra-strand cross-links, in particular with guanine and cytosine, change DNA conformation and inhibit DNA synthesis leading to the cytotoxic and anti-tumor effects of cisplatin. Although cisplatin seems to act as an alkylating agent, there are data to indicate that its mode and sites of action are different from those of nitrogen mustard and the standard alkylating agents and that cisplatin does not exhibit cross-resistance with other alkylating agents or nitrosoureas.

Metabolism and Elimination: Cisplatin is non-enzymatically transformed to one or more metabolites that are extensively protein bound and have minimal cytotoxic activity. The non-protein bound (unchanged) fraction is cytotoxic. Following bolus injection or infusion over a dose range of 40-140 mg/m^2 varying in length from 1-24 hours, from 10 to about 40% of the administered platinum is excreted in the urine in 24 hours. Renal clearance of free platinum exceeds the glomerular filtration rate, indicating that cisplatin or other platinum-containing molecules are actively secreted by the kidneys. Renal clearance of free platinum is nonlinear and variable, and is dependent on dose, urine flow rate, and individual variability in the extent of active secretion and possible tubular reabsorption.

Dosing, Administration, Preparation, Storage, and Stability: See Section 4.2 for dosing information. Refer to the current FDA-approved package insert for drug administration, preparation, storage, stability and special handling information.

How Supplied: Cisplatin is commercially available and will not be supplied free of charge to the patient. Refer to the current FDA-approved package insert.

Adverse Events: Adverse effects reported in 10% or more of patients receiving cisplatin include peripheral neuropathy, nausea, vomiting, diarrhea, myelosuppression, liver enzymes elevation, nephrotoxicity (acute renal failure and chronic renal insufficiency), alopecia, tissue irritation, and ototoxicity.

Human toxicity includes anorexia, nausea, vomiting, renal toxicity (with an elevation of BUN, creatinine, serum uric acid and impairment of endogenous creatinine clearance, as well as renal tubular damage), ototoxicity (with hearing loss which initially is in the high-frequency range, as well as tinnitus), peripheral neuropathy and hyperuricemia. Much more severe and prolonged toxicity has been observed in patients with abnormal or obstructed urinary excretory tracts. Raynaud's phenomena and digital ischemia has been described. Anaphylactic-like reactions including facial edema, bronchoconstriction, tachycardia and hypotension may occur within minutes of administration.

Myelosuppression, often with delayed erythrosuppression, is expected. In the high-dose treatment regimen with osmotic diuresis, the nadir of white cells and platelets occurred regularly at about two weeks with recovery generally at about three weeks after the initiation of therapy. Alopecia, malaise and asthenia have been reported. Rare complications are alopecia, seizures, loss of taste and allergic reactions. Tetany may occur due to hypomagnesemia and/or hypocalcemia. Other electrolyte disturbances may occur. At high doses patients have experienced optic neuritis, papilledema, cerebral blindness, blurred vision, and altered color perception. Patients have also experienced cardiac abnormalities, elevated aspartate aminotransferase and rash. Subsequent courses should not be given until serum creatinine returns to normal if elevated. Audiometric analyses should be monitored, and courses withheld until auditory acuity is within normal limits. The occurrence of acute leukemia has been reported rarely in patients treated with anthracycline/alkylator combination chemotherapy.

Refer to the current FDA-approved package insert for the most comprehensive and up to date information on adverse reactions.

Pregnancy and Lactation: Category D. Cisplatin can cause fetal harm when administered to a pregnant woman. In mice, cisplatin is teratogenic and embryotoxic. This drug has been found to be excreted in human milk and because of the potential for serious adverse reactions in nursing infants, patients receiving cisplatin should not breast feed.

Drug Interactions: During cisplatin therapy, plasma levels of anticonvulsant agents may become sub-therapeutic and should be monitored. For complete information refer to the current FDA-approved package insert.

6.0 EVALUATIONS AND ASSESSMENTS

6.1 Time and Events Table

The Time and Events Table below summarizes the trial procedures to be performed at each visit. Individual trial procedures are described in detail below. It may be necessary to perform these procedures at unscheduled time points if deemed clinically necessary by the investigator.

Furthermore, additional evaluations/testing may be deemed necessary by the Sponsor and/or Merck for reasons related to subject safety. In some cases, such evaluation/testing may be potentially sensitive in nature (e.g., HIV, Hepatitis C, etc.), and thus local regulations may require that additional informed consent be obtained from the subject. In these cases, such evaluations/testing will be performed in accordance with those regulations.

**Assessment**			**Neoadjuvant Chemotherapy plus Pembrolizumab**								**Pre-Surgery**[3]	**Surgery**[4]	**Post-Surgery Visit**[5]	**Long-term Follow-up**[6]
	**Pre-study**[1]	**D1[2] Cycle 1**	**D8[2] Cycle 1**	**D15[2] Cycle 1**	**D1[2] Cycle 2**	**D8[2] Cycle 2**	**D1[2] Cycle 3**	**D8[2] Cycle 3**	**D1[2] Cycle 4**	**D8[2] Cycle 4**				
Informed Consent	X													
History[7]	X	X			X		X		X		X			
Physical exam[7]	X	X			X		X		X		X			
Vital signs[7]	X	X	X		X	X	X	X	X	X	X			
Performance Status	X	X			X		X		X		X			
CT chest	X													
CT/MRI of Ab/Pelvis	X										X[8]			
Bone scan	X[9]													
Pregnancy test	X[10]													
CBC with differential	X	X	X	X	X	X	X	X	X	X	X			
Serum chemistries[11]	X	X	X[11]	X	X	X[11]	X	X[11]	X	X[11]	X			
Liver function tests[12]	X	X			X		X		X		X			
Thyroid panel[13]	X						X				X			
Uric acid, PTT	X										X			

PT/INR	X	X[14]			X[14]		X[14]		X[14]		X			
Urinalysis[15]	X													
Concomitant Meds	X	X			X		X		X		X			
Toxicity Assessment	X	X	X		X	X	X	X	X	X	X		X[5]	X
Symptom Assessment[16]	X	X	X		X	X	X	X	X	X	X			
Pembrolizumab		X			X		X		X					
Gemcitabine		X	X		X	X	X	X	X	X				
Cisplatin		X	X		X	X	X	X	X	X				
Tumor Samples	X[17]											X[17]		
Blood sample (required)	X[18]				X[18]							X[18]		
Survival analysis														X

Key to Time and Events Table Footnotes

[1]Unless otherwise noted, laboratory evaluations may be performed up to 10 days prior to first dose of protocol treatment. Laboratory assessments on cycle 1 day 1 do not need to be repeated if screening labs performed less than 72 hours prior to the scheduled visit. Imaging studies may be performed up to 30 days prior to first dose of protocol treatment.

[2]A window of +/-3 days will be applied to all study visits, except Day 8 visits each cycle will have a window of +/-1 day and Cycle 1 D15 labs will have a window of +/-1 day

[3] Pre-Surgery visit to take place approximately 30 days after last dose of pembrolizumab given. Also serves as standard pre-op assessment, and mandatory ~30-day post pembrolizumab safety visit. Patients who continue to experience ≥ Grade 2 treatment-related adverse event will be contacted every 2 weeks until the event is resolved to Grade 0 or 1, determined to be irreversible by the investigator, or until the patient begins an alternate form of drug treatment. This visit should occur in patients when treatment stops for any reason (toxicity, progression, or at discretion of the investigator) even if surgery no longer planned.

[4]Cystectomy should occur between 4-8 weeks, if possible, from the last dose of neoadjuvant chemotherapy.

[5]The post-surgery visit should be scheduled 4-8 weeks after surgery**.** Serious adverse events (SAEs) or any grade of Events of Clinical Interest (see section 7.3 that occur within 90 days of the end of pembrolizumab (or prior to start of new anti-cancer therapy) must be recorded. **NOTE**: Patients who do not complete neoadjuvant chemotherapy + pembrolizumab for whatever reason other than withdrawal from the study (see section 4.9), but still undergo surgery, will be requested to participate in correlative studies of excess surgical specimen (optional)

[6]Long-term follow-up will take place every 3 months (with window of +/-2 weeks) after surgery for the first 12 months, every 6 months (with window of +/-2 weeks) for the next 24 months, then annually (with window of +/- one month) until 5 years from enrollment. For study purposes, data gathered will be limited to history of any subsequent cancer treatments, any disease assessments performed as per standard of care, an assessment of any SAE's considered to be possibly or probably related to study treatment until resolution, and survival status.

[7]Complete history at baseline only, thereafter focused history on symptoms/toxicity. Physical exam (PE) to include height (baseline only), and weight; PE to be comprehensive at screening and pre-surgery; other scheduled PE to be directed based on symptoms; vital signs to include systolic and diastolic blood pressure, pulse rate and temperature.

[8]Repeat imaging must be done prior to surgery (30 days after last dose of pembrolizumab with a +/-14 day window). Imaging does not need to be repeated if patient is removed from protocol-directed therapy and imaging has been done within 30 days.

[9]Obtain at baseline in patients with any clinical or laboratory suspicion of metastatic bone involvement

[10]Serum or urine B-HCG within 72 hours prior to first dose of pembrolizumab and only if clinically appropriate (e.g. women of childbearing potential); if the urine test is positive or cannot be confirmed as negative, a serum pregnancy test will be required.

[11]Serum chemistries include Na, K, Cl, creatinine, BUN, calcium, magnesium, bicarbonate, phosphorus, and glucose. All serum chemistries should be drawn on day 1 and day 8 of each cycle.

[12] Liver function tests (LFTs) include bilirubin, alkaline phosphatase, ALT, AST, albumin, LDH, and total protein.

[13] Thyroid panel includes TSH, total or free T3 and free T4

[14] PT/INR for all subjects pre-study and pre-surgery. Patients on anticoagulation with warfarin should also have PT/INR checked each cycle on day 1.

[15]Urinalysis includes blood, glucose, protein, specific gravity, and microscopic exam if abnormal results are noted

[16]Patient-reported symptom assessment is a patient questionnaire derived from the Patient Reported Outcomes (PRO)-CTCAE and Patient Reported Outcomes Measurement Information System (PROMIS) (Appendix B)

[17]Pre-study archival tumor tissue will be requested from all patients; post-chemotherapy tissue to be obtained at cystectomy. Sample collection, storage, and shipment instructions for tumor tissue samples will be provided in the Laboratory Manual. See section 6.7 for additional information.

[18]Blood sample (7 x 8.5 mL ACD tubes) to be obtained for correlative studies prior to first dose of pembrolizumab, on cycle 2 day 1, and at time of cystectomy; sample collection, storage, and shipment instructions for serum samples will be provided in the Laboratory Manual.

6.2 Pre-Study Assessments

The assessments required as part of screening can be completed in one or more visits, as long as the assessments are completed within the time frames listed in the Key to the Time and Events table.

Clinical evaluation: complete history (including disease details with all prior treatments, all active medical conditions and any condition diagnosed within the prior 10 years that are considered to be clinically significant by the Investigator), comprehensive physical examination (to also include vital signs, height, and weight), ECOG performance status, QOL questionnaire
Laboratory studies:

- Pregnancy test: serum or urine pregnancy test is required for all women of childbearing potential within 72 hours prior to start of study treatment
- Hematology: CBC with differential
- Serum chemistries: Na, K, Cl, creatinine, BUN, calcium, magnesium, bicarbonate, phosphorus, glucose
- LFTs: AST, ALT, total bilirubin, alkaline phosphatase, albumin, LDH, total protein
- Coagulation and thyroid panel, and uric acid: PT/INR, PTT, TSH, total or free T3, free T4, uric acid
- Urinalysis
- Blood sample (required) for correlative studies: 7 x 8.5 mL ACD tubes

Staging: CT chest and CT abdomen/pelvis or MRI abdomen/pelvis. Imaging studies may be performed up to 4 weeks prior to first dose of study treatment.
Bone scan: Bone scan should be obtained at baseline in patients with any clinical or laboratory suspicion of metastatic bone involvement
Prior and concomitant medication review: Documentation of all medications taken by the subject within 28 days before starting the trial; documentation of all concomitant medications, and in particular any immunosuppressive medications.
Tissue acquisition: Primary tumor formalin-fixed, paraffin-embedded blocks will be collected. See section 6.5 and the laboratory manual for additional details.
Toxicity/QOL: Toxicity will be assessed according to the NCI CTCAE v 4.03, PRO-CTCAE, and PROMIS for notation of any baseline toxicity.

6.3 On-Study Treatment Assessments

6.3.1 Day 1 of Each Cycle of Pembrolizumab, Gemcitabine, and Cisplatin

Clinical evaluation: focused history, directed physical examination (to also include vital signs and weight), ECOG performance status
Laboratory studies:

- Hematology: CBC with differential

- Serum chemistries: Na, K, Cl, creatinine (calculate CrCl on day 1 of each cycle per CKD-Epi equation), BUN, calcium, magnesium, bicarbonate, phosphorus, glucose
- LFTs: AST, ALT, total bilirubin, alkaline phosphatase, albumin, LDH, total protein
- Thyroid function tests: TSH, total or free T3 and free T4 should also be drawn on cycle 3 day 1
- PT/INR: Should be drawn only if anticoagulated with warfarin
- Blood sample (required) for correlative studies: 7 x 8.5 mL ACD tubes on cycle 2 day 1 only

Concomitant medications: Documentation of any changes in concomitant medications
Toxicity/QOL: Toxicity will be assessed according to the NCI CTCAE v 4.03, PRO-CTCAE, and PROMIS.

6.3.2 Day 8 of Each Cycle

Clinical evaluation: Vital signs
Laboratory studies:

- Hematology: CBC with differential
- Serum chemistries: Na, K, Cl, creatinine (calculate CrCl per CKD-Epi equation), BUN, calcium, magnesium, bicarbonate, phosphorus, glucose

Toxicity/QOL: Toxicity will be assessed according to the NCI CTCAE v 4.03, PRO-CTCAE, and PROMIS.

6.3.3 Day 15 of Cycle 1

Laboratory studies:

- Hematology: CBC with differential
- Serum chemistries: Na, K, Cl, creatinine, BUN, calcium, magnesium, bicarbonate, phosphorus, glucose

6.4 Pre-Surgical Visit (post-pembrolizumab)

This visit should occur approximately 30 days after completion of last dose of pembrolizumab. This visit should serve as standard pre-operative visit prior to cystectomy.

Clinical evaluation: focused history on symptoms/toxicity, comprehensive physical examination (to include vital signs and weight), ECOG performance status
Laboratory studies:

- Hematology: CBC with differential

- Serum chemistries: Na, K, Cl, creatinine, BUN, calcium, magnesium, bicarbonate, phosphorus, glucose
- LFTs: AST, ALT, total bilirubin, alkaline phosphatase, albumin, LDH, total protein
- Coagulation and thyroid panel, and uric acid: PT/INR, PTT, TSH, total or free T3, free T4, uric acid

Tumor assessment: CT abdomen/pelvis or MRI abdomen/pelvis
Concomitant medications: Documentation of any changes in concomitant medications
Toxicity/QOL: Toxicity will be assessed according to the NCI CTCAE v 4.03. Patients who experience ≥ Grade 2 treatment related adverse event will be contacted every 2 weeks until the event is resolved to Grade 0 or 1, determined to be irreversible by the investigator, or until the patient begins an alternate form of drug treatment. Toxicity will also be assessed according to PRO-CTCAE and PROMIS with need for continued assessment until resolution as determined by the investigator.

6.5 Cystectomy

This should occur within 4-8 weeks, if possible, of the last dose of neoadjuvant chemotherapy. The surgery with curative intent will occur as per routine clinical standard of care and will be performed at the discretion of the surgeon. Enrollment into LCCC1520 should not impact this standard of care procedure.
Tissue acquisition: A sample of tumor tissue from the cystectomy will be preserved for correlative studies in those who do not achieve pT0. A sample of lymph node tissue from the lymph node biopsy and normal tissue from the cystectomy specimen will be preserved for correlative studies from all patients, including those who achieve pT0. These tissues will be preserved in consultation with surgical pathology, and tissue released for correlative studies post pathological review. **NOTE:** Patients who do not complete neoadjuvant chemotherapy + pembrolizumab for whatever reason other than withdrawal from the study (see section 4.9), but still undergo surgery, will be requested to participate in correlative studies of excess surgical specimen (optional).
Blood sample for correlative studies: 7 x 8.5 mL ACD tubes (see laboratory manual for details)

6.5.1 Post-Surgery Visit

This visit should be scheduled 4-8 weeks after cystectomy. Serious adverse events (SAEs) or any grade of Events of Clinical Interest (see section 7.3) that occur within 90 days of the end of pembrolizumab (or prior to start of new anti-cancer therapy) must be recorded. **NOTE**: Patients who do not complete neoadjuvant chemotherapy + pembrolizumab for whatever reason other than withdrawal from the study (see section 4.9), but still undergo surgery, will be requested to participate in correlative studies of excess surgical specimen (optional).

6.6 Long Term Follow-Up Assessments

After cystectomy, follow-up will take place every 3 months for the first 12 months (with +/-2 week window), every 6 months for the next 24 months (with +/- 2 week window), and then annually (with a +/- one month window) until 5 years from enrollment. For study purposes, data gathered will be limited to history of any subsequent cancer treatments, any disease assessments performed as per standard of care, an assessment of any SAE's considered to be possibly or probably related to study treatment until resolution, and survival status.

6.7 Correlative Studies Procedures

6.7.1 Archival Tumor Tissue

All subjects' archived primary tumor in paraffin-embedded blocks/slides will be requested for correlative studies.

mRNA sequencing will be performed for definition of tumor molecular subtypes, quantification of PD-1 ligand mRNA expression, and evaluation of gene expression signatures associated with immune activation and suppression. Whole exome sequencing will be done on tumors and matched blood samples for identification of somatic mutations and predicted MHC class I and class II neoantigens. T cell receptor (TCR) and B cell receptor (BCR) repertoires will be amplified, sequenced, and analyzed to discover receptor profiles that correlate with response to therapy. Tissue blocks/slides may be stained for coexpression using the following panel of fluorescent antibodies (can include but not limited to): CD4/FoxP3, CD8/PD1, CD19/CD27, CD11b/CD33, CD47/CD68, along with immunohistochemistry staining using the following antibodies: PD-L1, IFN-γ, TNF-α, CTLA4, KIR3DL1, LAG3, CD200, Tim3 [HAVCR2], BTLA, CD137 [TNFRSF9], CD40, OX40, CD27, ICOS, and GITR [TNFRSF18], which combined will enable quantification and localization of effector T cells, regulatory T cells, myeloid derived suppressor cells, antigen-experienced vs. non-experienced B cells, and suppressive macrophages, and enable definition of specific mechanisms of intratumoral cellular immune suppression.

Some of the specific correlative details regarding specific antibodies, repertoires etc. may vary from this protocol, but will be maintained and updated in the corresponding laboratory manual.

Slides of the pre-treatment TURBT will also be shipped to an external lab of the sponsor for assessment of PD-L1 expression.

6.7.2 Blood samples

Patients will have a seven ACD tubes (whole blood, 7 x 8.5 mL) collected at pre-study, on cycle 2 day 1, and at the time of cystectomy. Sample collection, storage,

and shipment instructions for serum samples will be provided in the Laboratory Manual.

6.7.3 Tumor and Lymph Node Tissue from Cystectomy

Tissue from primary tumor samples and lymph nodes removed at cystectomy will be collected as standard of care. Tissue reserved for correlative studies will undergo the same studies as outlined in section 6.7.1.

Tissue remaining after protocol-directed studies are complete will be destroyed, or, if requested, any unused slides will be returned to the site of origin. Additional details of correlative studies are provided in the accompanying Laboratory Manual.

6.7.4 Handling of Biospecimens Collected for Correlative Research

Biospecimens collected for this study will be stored in the Lineberger Comprehensive Cancer Center (LCCC) Tissue Procurement Facility (TPF), or if needed, in a secure off-site storage facility. All biospecimen samples will be obtained in accordance with procedures outlined in the LCCC1520 Study Laboratory Manual and stored in containers with controlled access. Each sample will be assigned a unique code number and no identifiable personal health information (PHI) will be on the specimen label. Information about the patient's disease will be linked to the specimens stored in the repository database. TPF-associated research staff, LCCC Bioinformatics staff who support the TPF database and the LCCC Data Warehouse, and researchers with IRB-approval for access to PHI for each subject in this study will be able to link specimens to relevant medical information. Some results from laboratory analyses that occurred during the patient's participation in the clinical study may also be included. This information may be important for understanding how the patient's cancer developed and responded to treatment.

Storage Time:

- The biospecimen will be used first and foremost for research purposes outlined within the confines of this protocol. Samples will be discarded/destroyed after relevant data are collected for this study; unless consent was obtained from the patient to use tissue for other research purposes (Specimen Consent checkbox was checked, and consent was signed).
- Leftover biospecimen material collected during this study may be stored and used for other research purposes including genetic research only if patient assent was obtained. In this circumstance, there is no time limit on how long biospecimens may be stored.

Compliance Statement

Biospecimen collection for this study will be conducted in full accordance to all applicable University of North Carolina (UNC) Research Policies and Procedures and all applicable Federal and state laws and regulations including 45 CFR 46, and the Health Insurance Portability and Accountability Act (HIPAA) Privacy Rule. Any episode of noncompliance will be documented.

The investigators will perform the study in accordance with this protocol, will obtain consent and assent (unless a waiver is granted), and will report unexpected problems in accordance with The UNC IRB Policies and Procedures and all federal requirements. Collection, recording, and reporting of data will be accurate and will ensure the privacy, health, and welfare of research subjects during and after the study.

6.8 Assessment of Safety

All patients who enroll and receive at least 1 dose of pembrolizumab will be evaluable for toxicity. Each patient will be assessed for the development of any toxicity according to the Time and Events table (section 6.0). Toxicity will be assessed according to the NCI CTCAE v. 4.03.

6.9 Assessment of Efficacy

All patients who enroll and receive at least 1 dose of pembrolizumab will be evaluable for the primary endpoint of pathologic downstaging. Those who undergo surgery will be evaluated for pathological downstaging at the time of surgery, and those who do not undergo surgery (due to progression of disease) will be considered to not have achieved pathologic downstaging.

6.9.1 Pathological Downstaging and Complete Response

The primary endpoint of this study is pathological downstaging. The rate of downstaging is defined as the proportion of patients who achieve absence of muscle-invasive disease at the time of cystectomy (<pT2). The proportion of patients with a pathologic complete response (no evidence of primary tumor (pT0)) is a secondary endpoint.

Pathologic staging is based on the operative and pathology report from cystectomy at the local site. Patients not undergoing surgery due to disease progression will be considered a nonresponder.

TX	Primary tumor cannot be assessed
T0	No evidence of primary tumor
Ta	Noninvasive papillary carcinoma
Tis	Carcinoma in situ: “flat tumor”
T1	Tumor invades subepithelial connective tissue
T2	Tumor invades muscularis propia
pT2a	Tumor invades superficial muscularis propia (inner half)
pT2b	Tumor invades deep muscularis propia (outer half)
T3	Tumor invades perivesical tissue

pT3a Microscopically
pT3b Macroscopically (extravesical mass)
T4 Tumor invades any of the following: prostatic stroma, seminal vesicles, uterus, vagina, pelvic wall, abdominal wall
T4a Tumor invades prostatic stroma, uterus, vagina
T4b Tumor invades pelvic wall, abdominal wall

NX Lymph nodes cannot be assessed
N0 No lymph node metastasis
N1 Single regional lymph node metastasis in the true pelvis (hypogastric, obturator, external iliac, or presacral lymph node)
N2 Multiple regional lymph metastases in the true pelvis (hypogastric, obturator, external iliac, or presacral lymph nodes)
N3 Lymph node metastases to the common iliac lymph nodes

M0 No distant metastasis
M1 Distant metastasis

6.9.2 Recurrence Criteria

Criteria for recurrence will include new measurable disease on cross-sectional imaging or plain radiography targeting lung, liver, and bone as most common sites for recurrence as per RECIST v1.1.

6.9.3 Event-Free Survival

The event-free time interval is from D1 of neoadjuvant treatment to date of first documentation of disease progression (in those who progress prior to surgery) or until recurrence (post-surgery) or death due to any cause. Recurrence includes the development of second primary urothelial malignancies. Patients last known to be alive without report of relapse/recurrence are censored at date of last contact. Criteria for recurrence will include measurable disease on cross-sectional imaging or plain radiography targeting lung, liver, and bone as most common sites for recurrence.

6.9.4 Overall Survival

Overall survival is measured from day 1 of treatment to date of death from any cause. Patients known to be alive are censored at date of last contact.

6.9.5 Overall Survival at 3 years

Overall survival at 3 years is defined as the proportion of patients alive at 3 years, measured from day 1 of treatment.

7.0 ADVERSE EVENTS

7.1 Definitions

7.1.1 Adverse Event (AE)

An adverse event (AE) is any untoward medical occurrence (e.g., an abnormal laboratory finding, symptom, or disease temporally associated with the use of a drug) in a patient or clinical investigation subject administered a pharmaceutical product and which does not necessarily have a causal relationship with this treatment. An AE can therefore be any unfavorable and unintended sign (including an abnormal laboratory finding), symptom, or disease temporally associated with the use of a medicinal product, whether or not related to the medicinal product.

Hospitalization for elective surgery or routine clinical procedures that are not the result of an AE (e.g., surgical insertion of central line) need not be considered AEs and should not be recorded as an AE. Disease progression should not be recorded as an AE, unless it is attributable by the investigator to the study therapy.

7.1.2 Suspected Adverse Reaction (SAR)

A suspected adverse reaction (SAR) is any AE for which there is a *reasonable possibility* that the drug is the cause. *Reasonable possibility* means that there is evidence to suggest a causal relationship between the drug and the AE. A suspected adverse reaction implies a lesser degree of certainty about causality than adverse reaction, which means any adverse event caused by a drug.

Causality assessment to a study drug is a medical judgment made in consideration of the following factors: temporal relationship of the AE to study drug exposure, known mechanism of action or side effect profile of study treatment, other recent or concomitant drug exposures, normal clinical course of the disease under investigation, and any other underlying or concurrent medical conditions. Other factors to consider in considering drug as the cause of the AE:

- Single occurrence of an uncommon event known to be strongly associated with drug exposure (e.g., angioedema, hepatic injury, Stevens-Johnson Syndrome)
- One or more occurrences of an event not commonly associated with drug exposure, but otherwise uncommon in the population (e.g., tendon rupture); often more than once occurrence from one or multiple studies would be needed before the sponsor could determine that there is *reasonable possibility* that the drug caused the event
- An aggregate analysis of specific events observed in a clinical trial that indicates the events occur more frequently in the drug treatment group than in a concurrent or historical control group

7.1.3 Unexpected AE or SAR

An AE or SAR is considered unexpected if the specificity or severity of it is not consistent with the applicable product information (e.g., Investigator's Brochure (IB) for an unapproved investigational product or package insert/summary of product characteristics for an approved product). Unexpected also refers to AEs or SARs that are mentioned in the IB as occurring with a class of drugs or as anticipated from the pharmacological properties of the drug but are not specifically mentioned as occurring with the particular drug under investigation.

7.1.4 Serious AE or SAR

An AE or SAR is considered serious if, in the view of either the investigator or sponsor, it results in any of the following outcomes:

- Death;
- Is life-threatening (places the subject at immediate risk of death from the event as it occurred);
- Requires inpatient hospitalization (>24 hours) or prolongation of existing hospitalization;*
- Results in congenital anomaly/birth defect;
- Results in a persistent or significant incapacity or substantial disruption of the ability to conduct normal life functions;
- Important medical events that may not result in death, be life-threatening, or require hospitalization may be considered a serious adverse drug experience when, based upon appropriate medical judgment, they may jeopardize the patient or subject and may require medical or surgical intervention to prevent one of the outcomes listed in the definition. For reporting purposes, also consider the occurrences of pregnancy as an event which must be reported as an important medical event.

*Hospitalization for anticipated or protocol specified procedures such as administration of chemotherapy, central line insertion, metastasis interventional therapy, resection of primary tumor, or elective surgery, will not be considered serious adverse events.

- **Note:** In addition to the above criteria, adverse events meeting either of the below criteria, although not serious per ICH definition, are reportable to the Merck in the same timeframe as SAEs to meet certain local requirements. Therefore, these events are considered serious by Merck for collection purposes.
    - Is a new cancer (that is not a condition of the study);
    - Is associated with an overdose.

Pregnancy that occurs during the study must be reported as an SAE within 120 days of completing the trial or within 30 days following cessation of treatment if the subject initiates new anticancer therapy. See section 7.3.3 for details on reporting requirements for pregnancy.

7.2 Documentation of non-serious AEs or SARs

For non-serious AEs or SARs, documentation must begin from day 1 of study treatment and continue through the 30-day follow-up period after treatment is discontinued.

Collected information should be recorded in the Case Report Forms (CRF) for that patient. Please include a description of the event, its severity or toxicity grade, onset and resolved dates (if applicable), and the relationship to the study drug. Documentation should occur at least monthly.

NOTE: As a part of continuous monitoring for toxicity, any treatment related grade 4 or 5 adverse events occurring during the first cycle of concomitant therapy must be reported to the UNC Multicenter Project Manager at CPOMulticenter@med.unc.edu immediately when they occur.

7.3 SAEs or Serious SARs and Events of Clinical Interest

7.3.1 Timing

After informed consent but prior to initiation of study medications, only SAEs caused by a protocol-mandated intervention will be collected (e.g. SAEs related to invasive procedures such as biopsies, medication washout).

For any other experience or condition that meets the definition of an SAE or a serious SAR, recording of the event must begin from day 1 of study treatment and continue through the 90-day follow-up period after treatment is discontinued (or to the initiation of new anti-cancer treatment, whichever is earliest).

7.3.2 Documentation and Notification

These events (SAEs or Serious SARs) must be recorded in the SAE console within OnCore® for that patient within 24 hours of learning of its occurrence. For Affiliate sites, an email must also be sent to the University of North Carolina Cancer Network UNC Multicenter Project Manager indicating that an SAE or Serious SAR has been entered into Oncore (email contact will be provided at study start-up).

In addition, SAE and SARS reports and any other relevant safety information are to be forwarded to the Merck Global Safety facsimile number: +1-215-993-1220 within 2 working days. This includes death due to any cause other than progression of the cancer under study that occurs to any subject from the time the consent is signed through 90 days following cessation of treatment, or the initiation of new anti-cancer therapy, whichever is earlier, whether or not related to Merck product. All subjects with serious adverse events must be followed up for outcome.

Additionally, any serious adverse event, considered by an investigator who is a qualified physician to be related to Merck product that is brought to the attention of the investigator at any time following consent through the end of the specified safety follow-up period specified in the paragraph above, or at any time outside of the time period specified in the previous paragraph also must be reported immediately to the Sponsor and to Merck Global Safety.

7.3.3 Reporting

IRB Reporting Requirements:

UNC:

- The UNC-IRB will be notified of all SAEs that qualify as an Unanticipated Problem as per the UNC IRB Policies using the IRB's web-based reporting system (see section 9.5.3) within 7 days of the Investigator becoming aware of the problem.

Affiliate sites:

- For affiliate sites using a local IRB of record, please submit adverse events per local IRB policy.

  In addition, any SAEs that qualify as an Unanticipated Problem will be entered into Oncore and reported to the UNC IRB by the UNC Multicenter Project Manager using the IRB's web-based reporting system (see section 9.5.3) within 7 days of the Investigator becoming aware of the problem.

Pregnancy

Although pregnancy and lactation are not considered adverse events, it is the responsibility of investigators or their designees to report any pregnancy or lactation in a subject (spontaneously reported to them) that occurs during the trial.

Pregnancies and lactations that occur after the consent form is signed but before treatment must be reported by the investigator if they cause the subject to be excluded from the trial or are the result of a protocol-specified intervention, including but not limited to washout or discontinuation of usual therapy, diet, placebo treatment or a procedure.

It is the responsibility of investigators or their designees to report any pregnancy or lactation in a subject (spontaneously reported to them), including the pregnancy of a male subject's female partner that occurs during the trial or within 120 days of completing the trial completing the trial, or 30 days following cessation of treatment if the subject initiates new anticancer therapy, whichever is earlier, as a serious adverse event. The patient is to be discontinued immediately from any protocol directed therapy. All subjects and female partners of male subjects who become pregnant must be followed to the completion/termination of the pregnancy. Pregnancy outcomes of spontaneous

abortion, missed abortion, benign hydatidiform mole, blighted ovum, fetal death, intrauterine death, miscarriage and stillbirth must be reported as serious adverse events. If the pregnancy continues to term, the outcome (health of infant) must also be reported. Such events must be reported within 24 hours to the Sponsor and within 2 days to Merck Global Safety. (Attn: Worldwide Product Safety; Fax: 215 993-1220).

For Affiliate sites, the pregnancy, suspected pregnancy, or positive pregnancy test must be reported to the UNC Multicenter Project Manager within 24 hours via facsimile to 919-966-4300. The female subject should be referred to an obstetrician-gynecologist, preferably one experienced in reproductive toxicity for further evaluation and counseling.

7.3.4 FDA Expedited Reporting requirements for studies conducted under an IND:

A sponsor must report any suspected adverse reaction that is both serious and unexpected to the FDA. The sponsor must report an adverse event as a suspected adverse reaction only if there is evidence to suggest a causal relationship between the drug and the adverse event, such as:

- A single occurrence of an event that is uncommon and known to be strongly associated with drug exposure (e.g., angioedema, hepatic injury, Stevens-Johnson Syndrome);
- One or more occurrences of an event that is not commonly associated with drug exposure, but is otherwise uncommon in the population exposed to the drug (e.g. tendon rupture);
- An aggregate analysis of specific events observed in a clinical trial (such as known consequences of underlying disease or condition under investigation or other events that commonly occur in the study population independent of drug therapy) that indicates those events occur more frequently in the drug treatment group than in a concurrent or historical control group.

The sponsor must submit each IND safety report on FDA Form 3500A. Each notification to FDA must bear prominent identification of its contents, i.e., "IND Safety Report," and must be transmitted to the review division that has the responsibility for review of the IND. For this study, the review division is the Center for Drug Evaluation and Research. In each IND safety report, the sponsor must identify all IND safety reports previously submitted to FDA concerning a similar suspected adverse reaction and must analyze the significance of the suspected adverse reaction in light of previous, similar reports or any other relevant information.

Timing

FDA must be notified of potential serious risks within 15 calendar days after the sponsor determines the event requires reporting. FDA must be notified of unexpected fatal or life-threatening suspected adverse reactions as soon as

possible but in no case later than 7 calendar days after the sponsor's initial receipt of the information. The sponsor must be notified of the SAE by the investigator within 24 hours of the event. If the results of a sponsor's investigation show that an adverse event not initially determined to be reportable is reportable, the sponsor must report such suspected adverse reaction in an IND safety report as soon as possible, but in no case later than 15 calendar days after the determination is made.

Follow-up
The sponsor must promptly investigate all safety information it receives. Relevant follow-up information to an IND safety report must be submitted as soon as the information is available and must be identified as such, i.e., "Follow-up IND Safety Report." Additionally, upon request from FDA, the sponsor must submit to FDA any additional data or information that the agency deems necessary, as soon as possible, but in no case later than 15 calendar days after receiving the request.

Notification of Investigators
The sponsor must notify all participating investigators (i.e., all investigators to whom the sponsor is providing drug under its INDs or under any investigator's IND) in an IND safety report of potential serious risks, from clinical trials or any other source, as soon as possible, but in no case later than 15 calendar days after the sponsor determines that the information qualifies for reporting.

Process
If the sponsor deems that an event is both a serious adverse reaction (SAR) AND unexpected, it must also (in addition to OnCore®) be recorded on the MedWatch Form 3500A as per 21 CFR 312.32. Unexpected adverse events or adverse reaction refers to an event or reaction that is not listed in the investigator's brochure or is not listed at the specificity or severity that has been observed; or if an investigator's brochure is not required or available, is not consistent with the risk information described in the general investigation plan or elsewhere in the current IND application.

The MedWatch form should be faxed to the UNC Multicenter Project Manager at 919-966-4300 (or emailed, with address provided at the Start up Meeting (SIM)) along with supporting documentation defining the event and causality. The UNC Multicenter Project Manager will then send the report to the Funding Source. The MedWatch 3500A form can be accessed at: http://www.fda.gov/Safety/MedWatch/HowToReport/DownloadForms/default.htm.

(Please be sure and access form 3500A, and not form 3500).

Once the UNC Principal Investigator works with the sponsor to determine an event is a serious SAR AND unexpected, the MedWatch 3500A form will be submitted to the FDA. If the event is serious, unexpected and considered to be possibly-, probably- or definitely-related to the study treatment, the UCCN Project Manager

will inform the Regulatory Associate at UNC, who with the aid of the IND Specialist, will submit the IND Safety Report via IND serial submission to the FDA review division.

All IND safety reports must be submitted on Form 3500A and be accompanied by Form 1571. The FDA must be notified or any unexpected or life-threatening suspected adverse reactions as soon as possible, but no later than 7 calendar days of learning of the event.

The UNC Multicenter Project Manager will also be responsible for informing each Affiliate site of all serious and unexpected SARs reported to the FDA via fax as soon as possible.

Additional Reporting Requirements
The following additional items must be reported via IND safety report:

- *Findings from other studies.* The sponsor must report any findings from epidemiological studies, pooled analysis of multiple studies, or clinical studies, whether or not conducted under an IND, and whether or not conducted by the sponsor, that suggest a significant risk to humans exposed to the drug.
- *Findings from animal or in vitro testing.* The sponsor must report any findings from animal or *in vitro* testing, whether or not conducted by the sponsor, that suggest a significant risk in humans exposed to the drug, such as reports of mutagenicity, teratogenicity, or carcinogenicity, or reports of significant organ toxicity t or near the expected human exposure.
- *Increased rate of occurrence of serious suspected adverse reactions.*

Additional Guidance
Please refer to 21CFR312.32 and "Guidance for Industry and Investigators: Safety Reporting Requirements for INDs and BA/BE Studies" for additional information and reporting requirements. All IND Safety Reports will be submitted in accordance with these regulations/guidances.

Merck Reporting Requirements:

As noted SAE/SAR reports and any other relevant safety information are to be forwarded to the Merck Global Safety facsimile number: +1-215-993-1220

All 15-Day Reports and Annual Progress Reports must be submitted as required to FDA. Investigators will cross-reference these reports to the Merck Investigational Compound Number (IND, CSA, etc.) at the time of submission. Additionally, investigators will submit a copy of these reports to Merck & Co., Inc. (Attn: Worldwide Product Safety; FAX 215 993-1220) at the time of submission to FDA.

In addition to SAE and SARS reports, Non-serious Events of Clinical Interest will also be forwarded to Merck Global Safety and will be handled in the same manner as SAEs.

Events of Clinical Interest

Selected non-serious and serious adverse events are also known as Events of Clinical Interest (ECI). ECI must be recorded as such on the Adverse Event case report forms/worksheets and reported within 24 hours to the Sponsor and within 2 working days to Merck Global Safety. (Attn: Worldwide Product Safety; FAX 215 993-1220)

For the time period beginning when the consent form is signed until treatment allocation/randomization, any ECI, or follow up to an ECI, that occurs to any subject must be reported within 24 hours to the Sponsor and within 2 working days to Merck Global Safety if it causes the subject to be excluded from the trial, or is the result of a protocol-specified intervention, including but not limited to washout or discontinuation of usual therapy, diet, placebo treatment or a procedure.

For the time period beginning at treatment through 90 days following cessation of treatment, or 30 days following cessation of treatment if the subject initiates new anticancer therapy, whichever is earlier, any ECI, or follow up to an ECI, whether or not related to Merck product, must be reported within 24 hours to the Sponsor and within 24 hours to Merck Global Safety.

<u>Events of clinical interest for this trial include:</u>

1. An overdose of Merck product, as defined 5.1.14
2. An elevated AST or ALT lab value that is greater than or equal to 3X the upper limit of normal and an elevated total bilirubin lab value that is greater than or equal to 2X the upper limit of normal (or greater than or equal to 3X the upper limit of normal in patients with Gilbert's syndrome) and, at the same time, an alkaline phosphatase lab value that is less than 2X the upper limit of normal, as determined by way of protocol-specified laboratory testing or unscheduled laboratory testing.* *Note: These criteria are based upon available regulatory guidance documents. The purpose of the criteria is to specify a threshold of abnormal hepatic tests that may require an additional evaluation for an underlying etiology.

<u>Overdose</u>

If a dose of Merck's product meeting the protocol definition of overdose is taken without any associated clinical symptoms or abnormal laboratory results, the overdose is reported as a non-serious Event of Clinical Interest (ECI), using the terminology "accidental or intentional overdose without adverse effect."
All reports of overdose with and without an adverse event must be reported within 24 hours to the Sponsor and within 2 working days hours to Merck Global Safety. (Attn: Worldwide Product Safety; Fax: 215 993-1220).

7.4 Data and Safety Monitoring Plan

The Principal Investigator will provide continuous monitoring of patient safety in this trial with periodic reporting to the Data and Safety Monitoring Committee (DSMC).

Meetings/teleconferences will be held at a frequency dependent on study accrual, and in consultation with the study Biostatistician. These meetings will include the investigators as well as protocol nurses, clinical research associates, regulatory associates, data managers, biostatisticians, and any other relevant personnel the principal investigators may deem appropriate. At these meetings, the research team will discuss all issues relevant to study progress, including enrollment, safety, regulatory, data collection, etc.

The team will produce summaries or minutes of these meetings. These summaries will be available for inspection when requested by any of the regulatory bodies charged with the safety of human subjects and the integrity of data including, but not limited to, the oversight (Office of Human Research Ethics (OHRE) Biomedical IRB, the Oncology Protocol Review Committee (PRC) or the North Carolina TraCS Institute Data and Safety Monitoring Board (DSMB).

The UNC LCCC Data and Safety Monitoring Committee (DSMC) will review the study on a regular (quarterly to annually) basis, with the frequency of review based on risk and complexity as determined by the UNC Protocol Review Committee. The UNC PI will be responsible for submitting the following information for review: 1) safety and accrual data including the number of patients treated; 2) significant developments reported in the literature that may affect the safety of participants or the ethics of the study; 3) preliminary response data; and 4) summaries of team meetings that have occurred since the last report. Findings of the DSMC review will be disseminated by memo to the UNC PI, PRC, and the UNC IRB and DSMB.

8.0 STATISTICAL CONSIDERATIONS

8.1 Study Design/Study Endpoints

This multicenter phase II trial seeks to evaluate pembrolizumab in combination with gemcitabine and cisplatin as neoadjuvant therapy prior to radical cystectomy using a Simon's two-stage design. This is an open-label trial; therefore, the Sponsor, investigator, and subject will know the treatment administered.

The study will enroll 39 evaluable patients, using a Simon's two-stage design, with a primary objective of estimating the rate of pathological downstaging to <pT2. Secondary objectives include toxicity assessment, complete pathologic response rate (pT0), event-free survival (EFS), overall survival (OS), and OS at 3 years. Exploratory objectives include an evaluation of the predictive capacity of PD-L1 expression, immune gene signatures, and RNA subtypes of bladder cancer, and a characterization of the change in phenotype in tumor-infiltrating lymphocytes before and after neoadjuvant therapy.

8.2 Sample Size and Accrual

The primary endpoint of this study is rate of pathological downstaging, defined as the proportion of patients who achieve absence of muscle-invasive disease at the time of cystectomy (<pT2). Based on a prior retrospective study [14] and two prospective randomized clinical trials (MRC/EORTC trial and Intergroup 0080 trial) utilizing a conventional schedule of cisplatin-based neoadjuvant chemotherapy, the null pathological downstaging rate is 35%.[5, 16]

A Simon's two-stage minimax design will be used for this study.[66] The null hypothesis that the true pathological downstaging rate is 35% will be tested against a one-sided alternative that it is higher. In the first stage, 21 patients will be accrued. If there are 8 or fewer patients with pathologic downstaging (<pT2) in these 21 patients, then the study will be stopped for futility, and the treatment regimen would be considered as uninteresting. Otherwise, 18 additional patients will be accrued for a total of 39. The null hypothesis will be rejected if 19 or more of the 39 patients have been downstaged to <pT2, and the treatment regimen will be considered worthy of future investigation. This design yields a type I error rate of 0.05 and power of 80% when the true response rate is 55%, representing a 20% improvement on the pathological downstaging rate previously reported. (http://cancer.unc.edu/biostatistics/program/ivanova/SimonsTwoStageDesign.aspx)

Accrual of 39 patients is estimated to take 3 years.

8.3 Continuous Monitoring for Toxicity

Sequential boundaries will be used to suspend the trial if unacceptable toxicity is seen in subjects enrolled after the approval of Amendment 9 of this protocol. If the study reaches a stopping boundary, it may be terminated by the PI, or submitted to the Data and Safety Monitoring Committee with a description of the

failures to date and a rationale for why the study should be continued. An unacceptable toxicity is defined as any treatment-related grade 4-5 toxicity through the first cycle of concomitant therapy. The accrual will be halted if the number of patients with unacceptable toxicities is equal to or exceeds b_n out of n patients (see table below). This is a Pocock-type stopping boundary that yields the probability of crossing the boundary at most 0.10 when the rate of unacceptable toxicity is equal to the acceptable rate, 0.2. [http://cancer.unc.edu/biostatistics/program/ivanova/ContinuosMonitoringForToxicity.aspx]

Number of Patients, *n*	1	2	3	4	5	6	7	8	9	10	11	12	13	14	15	16	17	18	19	20
Boundary, b_n	-	-	3	3	4	4	5	5	5	6	6	6	6	7	7	7	8	8	8	8
Number of Patients, *n*	21	22	23	24	25	26	27	28	29	30	31	32	33	34	35	36	37	38	39	
Boundary, b_n	9	9	9	10	10	10	10	11	11	11	11	12	12	12	13	13	13	13	14	

8.4 Data Analysis Plans

The proportion of patients who have pathological downstaging to <pT2 will be reported along with a 95% confidence interval. Patients who progress prior to surgery (expected to be a small number), and do not have surgery, will still be included in this estimate and will be considered to have not been downstaged. Similarly, the proportion of patients who have a complete response (pT0) will be estimated.

The Kaplan-Meier method will be used to estimate median times, along with 95% confidence intervals, for the secondary objectives of EFS and OS, as defined in 6.9.3 and 6.9.4. Toxicity data will be summarized and reported, and continuously monitored, as described in 8.3. Quality of life data from PRO will also be summarized and reported. To further summarize safety, we will report on treatment delays and the percentage of patients who are unable to proceed to cystectomy due to treatment-related toxicities.

For exploratory objectives, the association of biological markers with EFS and OS will be analyzed using Log Rank tests and Cox regression models. Fisher's Exact tests will be used to compare the response rates between subtypes of MIBC (basals versus luminals). For each T cell phenotype, the change in percentage of T cells will be calculated at the patient level, and descriptive statistics will be provided. TCR and BCR repertoire profiles will be characterized as having high or low sequence diversity, and the association of these categorical variables with response rate and EFS/OS will be made using Fisher's Exact tests and Log Rank tests.

9.0 STUDY MANAGEMENT

9.1 Institutional Review Board (IRB) Approval and Consent

It is expected that the IRB will have the proper representation and function in accordance with federally mandated regulations. The IRB should approve the consent form and protocol.

In obtaining and documenting informed consent, the investigator should comply with the applicable regulatory requirement(s) and should adhere to Good Clinical Practice (GCP) and to ethical principles that have their origin in the Declaration of Helsinki.

Before recruitment and enrollment onto this study, the patient will be given a full explanation of the study and will be given the opportunity to review the consent form. Each consent form must include all the relevant elements currently required by the FDA Regulations and local or state regulations. Once this essential information has been provided to the patient and the investigator is assured that the patient understands the implications of participating in the study, the patient will be asked to give consent to participate in the study by signing an IRB-approved consent form.

Prior to a patient's participation in the trial, the written informed consent form should be signed and personally dated by the patient and by the person who conducted the informed consent discussion.

9.2 Required Documentation

Before the study can be initiated at any site, the following documentation must be provided to the Clinical Protocol Office (CPO) at the University of North Carolina.

- A copy of the official IRB approval letter for the protocol and informed consent
- IRB membership list
- Financial Disclosures
- CVs and medical licensure for the principal investigator and any sub-investigators who will be involved in the study.
- Form FDA 1572 appropriately filled out and signed with appropriate documentation
- CAP and CLIA Laboratory certification numbers and institution lab normal values
- Executed clinical research contract

9.3 Registration Procedures

All patients must be registered with the CPO at the University of North Carolina before enrollment to study. For UNC patients, prior to registration, eligibility

criteria must be confirmed with the UNC Project Manager. To register a patient, call the Oncology Protocol Office at 919-966-7359 Monday through Friday, 9:00AM-5:00PM.

Affiliate sites: All subjects must be registered with the LCCC CPO Multicenter Office at the University of North Carolina before enrollment to study. To register a subject call the Multicenter office at 919-966-7359 Monday-Friday 8:30 am – 5:00 pm EST. Scan and email the UNC Project Manager (CPOMultiCenter@med.unc.edu; preferred) or fax (919-966-4300) the registration form, signed informed consents, signed eligibility form and all source documents to confirm eligibility. Eligibility may be confirmed by the UNC Study Coordinator for subjects treated at UNC. When sending registration request with eligibility documentation, please allow 24 hours for source to be reviewed.

9.4 Data Management and Monitoring/Auditing

The CPO of the UNC LCCC will serve as the coordinating center for this trial. Data will be collected through a web based clinical research platform, OnCore®. Other study institutions will be given a password to directly enter their own data onto the web site via electronic case report forms (eCRFs). UNC Multicenter personnel will coordinate and manage data for quality control assurance and integrity.

All data will be collected and entered into OnCore® by Clinical Research Associates (CRAs) from UNC LCCC and participating institutions. The investigators at each site will allow monitors to review all source documents supporting data entered into OnCore®. The UNC Multicenter Data Coordinator can be reached at 919-843-2742 or 1-877-668-0683.

All data will be monitored, and source data will be verified on selected subjects. Participating sites must send source documents to LCCC upon request, for remote monitoring review. Queries will be issued on an ongoing basis on all subjects. Participating sites should respond to data queries within 14 days of receipt.

As an investigator initiated study, this trial will also be audited by the Lineberger Cancer Center compliance committee every six or twelve months, depending on the participation of affiliate sites.

9.5 Adherence to the Protocol

Except for an emergency situation in which proper care for the protection, safety, and well-being of the study patient requires alternative treatment, the study shall be conducted exactly as described in the approved protocol.

9.5.1 Emergency Modifications

UNC and Affiliate investigators may implement a deviation from, or a change of, the protocol to eliminate an immediate hazard(s) to trial subjects without prior UNC or their respective institution's IRB/IEC approval/favorable opinion.

For Institutions Relying on UNC's IRB:
For any such emergency modification implemented, a UNC IRB modification form must be completed by UNC Research Personnel within five (5) business days of making the change.

For Institutions Relying on Their Own IRB:
For Affiliate investigators relying on their own institution's IRB, as soon as possible after the modification has been made, the implemented deviation or change and the reasons for it should be submitted to:

- To UNC Principal Investigator for agreement
- The Affiliate institution's IRB for review and approval. (Once IRB's response is received, this should be forwarded to the UNC Multicenter Regulatory Associate).

9.5.2 Single Patient/Subject Exceptions

Eligibility single subject exceptions are not permitted for Lineberger Comprehensive Cancer Center Investigator Initiated Trials under any circumstances. Other types of single subject exceptions may be allowed if proper regulatory review has been completed in accordance with Lineberger Comprehensive Cancer Center's Single Subject Exceptions Policy.

9.5.3 Other Protocol Deviations/Violations

According to UNC's IRB, a protocol deviation is any unplanned variance from an IRB approved protocol that:

- Is generally noted or recognized after it occurs
- Has no substantive effect on the risks to research participants
- Has no substantive effect on the scientific integrity of the research plan or the value of the data collected
- Did not result from willful or knowing misconduct on the part of the investigator(s).

An unplanned protocol variance is considered a violation if the variance meets any of the following criteria:

- Has harmed or increased the risk of harm to one or more research participants.
- Has damaged the scientific integrity of the data collected for the study.
- Results from willful or knowing misconduct on the part of the investigator(s).
- Demonstrates serious or continuing noncompliance with federal

regulations, State laws, or University policies.

If a deviation or violation occurs, please follow the guidelines below:

For Institutions Relying on UNC's IRB:
Protocol Deviations: UNC or Affiliate personnel will record the deviation in OnCore®, and report to any sponsor or data and safety monitoring committee in accordance with their policies. Deviations should be summarized and reported to the IRB at the time of continuing review.

Protocol Violations: Violations should be reported by UNC personnel within one (1) week of the investigator becoming aware of the event using the same IRB online mechanism used to report Unanticipated Problems.

For Institutions Relying on Their Own IRB:
In addition to adhering to the policies regarding protocol compliance set forth by your institution's IRB, the following is also required:

Protocol Deviations: In the event a deviation from protocol procedures is identified, record the deviation in OnCore®.

Protocol Violations: Any protocol violation that occurs must be reported to your IRB per institutional policies and reported to the UNC Multicenter Project Manager within 5 days. UNC-CH will determine if the violation affects the safety of the patient and integrity of the data. Once your institution's IRB response is received, please forward to the UNC Multicenter Regulatory Associate.

Unanticipated Problems:
Affiliate Sites:
Any events that meet the criteria for "Unanticipated Problems (UPs)" as defined by UNC's IRB must also be reported to the UNC Multicenter Project Manager. The UNC Multicenter Project Manager will report the event to the UNC IRB using the IRB's web-based reporting system. Examples of such UPs include a lost or stolen laptop computer that contains sensitive study information.

UNC
Any events that meet the criteria for "Unanticipated Problems" as defined by UNC's IRB must be reported by the study team using the IRB's web-based reporting system.

9.6 Amendments to the Protocol

Should amendments to the protocol be required, the amendments will be originated and documented by the Principal Investigator at UNC. It should also be noted that when an amendment to the protocol substantially alters the study design or the potential risk to the patient, a revised consent form might be required.

For Institutions Relying on UNC's IRB:
The written amendment, and if required the amended consent form, must be sent to UNC's IRB for approval prior to implementation.

For Institutions Relying on Their Own IRB:
Investigators must submit the UNC IRB approved amendment to their institution's IRB for approval. For multi-center studies, any affiliate site must submit their informed consent revisions to the UNC Multicenter Regulatory Associate prior to submission to their IRB.

9.7 Record Retention

Study documentation includes all eCRFs, data correction forms or queries, source documents, Sponsor-Investigator correspondence, monitoring logs/letters, and regulatory documents (e.g., protocol and amendments, IRB correspondence and approval, signed patient consent forms).

Source documents include all recordings of observations or notations of clinical activities and all reports and records necessary for the evaluation and reconstruction of the clinical research study.

Government agency regulations and directives require that all study documentation pertaining to the conduct of a clinical trial must be retained by the study investigator. In the case of a study with a drug seeking regulatory approval and marketing, these documents shall be retained for at least two years after the last approval of marketing application in an International Conference on Harmonization (ICH) region. In all other cases, study documents should be kept on file until three years after the completion and final study report of this investigational study.

9.8 Compliance with Trial Registration and Results Posting Requirements

Under the terms of the Food and Drug Administration Modernization Act (FDAMA) and the Food and Drug Administration Amendments Act (FDAAA), the Sponsor of the trial is solely responsible for determining whether the trial and its results are subject to the requirements for submission to the Clinical Trials Data Bank, http://www.clinicaltrials.gov. Information posted will allow subjects to identify potentially appropriate trials for their disease conditions and pursue participation by calling a central contact number for further information on appropriate trial locations and trial site contact information.

9.9 Obligations of Investigators

The Principal Investigator is responsible for the conduct of the clinical trial at the site in accordance with Title 21 of the Code of Federal Regulations and/or the Declaration of Helsinki. The Principal Investigator is responsible for personally overseeing the treatment of all study patients. The Principal Investigator must

assure that all study site personnel, including sub-investigators and other study staff members, adhere to the study protocol and all FDA/GCP/NCI regulations and guidelines regarding clinical trials both during and after study completion.

The Principal Investigator at each institution or site will be responsible for assuring that all the required data will be collected and entered into the eCRFs. Periodically, monitoring visits will be conducted and the Principal Investigator will provide access to his/her original records to permit verification of proper entry of data. At the completion of the study, all eCRFs will be reviewed by the Principal Investigator and will require his/her final signature to verify the accuracy of the data.

10.0 REFERENCES

1. Siegel, R., et al., *Cancer statistics, 2014.* CA Cancer J Clin, 2014. **64**(1): p. 9-29.
2. Nowak, A.K., B.W. Robinson, and R.A. Lake, *Gemcitabine exerts a selective effect on the humoral immune response: implications for combination chemo-immunotherapy.* Cancer Res, 2002. **62**(8): p. 2353-8.
3. Grossman, H.B., et al., *Neoadjuvant chemotherapy plus cystectomy compared with cystectomy alone for locally advanced bladder cancer.* N Engl J Med, 2003. **349**(9): p. 859-66.
4. *Neoadjuvant cisplatin, methotrexate, and vinblastine chemotherapy for muscle-invasive bladder cancer: a randomised controlled trial. International collaboration of trialists.* Lancet, 1999. **354**(9178): p. 533-40.
5. *Neoadjuvant chemotherapy in invasive bladder cancer: update of a systematic review and meta-analysis of individual patient data advanced bladder cancer (ABC) meta-analysis collaboration.* Eur Urol, 2005. **48**(2): p. 202-5; discussion 205-6.
6. Stein, J.P., et al., *Radical cystectomy in the treatment of invasive bladder cancer: long-term results in 1,054 patients.* J Clin Oncol, 2001. **19**(3): p. 666-75.
7. Sternberg, C.N., et al., *Preliminary results of M-VAC (methotrexate, vinblastine, doxorubicin and cisplatin) for transitional cell carcinoma of the urothelium.* J Urol, 1985. **133**(3): p. 403-7.
8. Loehrer, P.J., Sr., et al., *A randomized comparison of cisplatin alone or in combination with methotrexate, vinblastine, and doxorubicin in patients with metastatic urothelial carcinoma: a cooperative group study.* J Clin Oncol, 1992. **10**(7): p. 1066-73.
9. von der Maase, H., et al., *Long-term survival results of a randomized trial comparing gemcitabine plus cisplatin, with methotrexate, vinblastine, doxorubicin, plus cisplatin in patients with bladder cancer.* J Clin Oncol, 2005. **23**(21): p. 4602-8.
10. Adamo, V., et al., *Phase II study of gemcitabine and cisplatin in patients with advanced or metastatic bladder cancer: long-term follow-up of a 3-week regimen.* Oncology, 2005. **69**(5): p. 391-8.
11. Soto Parra, H., et al., *Three-week versus four-week schedule of cisplatin and gemcitabine: results of a randomized phase II study.* Ann Oncol, 2002. **13**(7): p. 1080-6.
12. Maughan, B.L., et al., *Pooled analysis of phase II trials evaluating weekly or conventional cisplatin as first-line therapy for advanced urothelial carcinoma.* Clin Genitourin Cancer, 2013. **11**(3): p. 316-20.
13. Hussain, S.A., et al., *A study of split-dose cisplatin-based neo-adjuvant chemotherapy in muscle-invasive bladder cancer.* Oncol Lett, 2012. **3**(4): p. 855-859.
14. Dash, A., et al., *A role for neoadjuvant gemcitabine plus cisplatin in muscle-invasive urothelial carcinoma of the bladder: a retrospective experience.* Cancer, 2008. **113**(9): p. 2471-7.
15. Chism, D.D., M.E. Woods, and M.I. Milowsky, *Neoadjuvant paradigm for accelerated drug development: an ideal model in bladder cancer.* Oncologist, 2013. **18**(8): p. 933-40.
16. Griffiths, G., et al., *International phase III trial assessing neoadjuvant cisplatin, methotrexate, and vinblastine chemotherapy for muscle-invasive bladder cancer: long-term results of the BA06 30894 trial.* J Clin Oncol, 2011. **29**(16): p. 2171-7.

17. Sonpavde, G., et al., *Single agent taxane versus taxane-containing combination chemotherapy as salvage therapy for advanced urothelial carcinoma.* European Urology (in press), 2015.
18. Ko, H.J., et al., *A combination of chemoimmunotherapies can efficiently break self-tolerance and induce antitumor immunity in a tolerogenic murine tumor model.* Cancer Res, 2007. **67**(15): p. 7477-86.
19. Suzuki, E., et al., *Gemcitabine selectively eliminates splenic Gr-1+/CD11b+ myeloid suppressor cells in tumor-bearing animals and enhances antitumor immune activity.* Clin Cancer Res, 2005. **11**(18): p. 6713-21.
20. Tongu, M., et al., *Metronomic chemotherapy with low-dose cyclophosphamide plus gemcitabine can induce anti-tumor T cell immunity in vivo.* Cancer Immunol Immunother, 2013. **62**(2): p. 383-91.
21. Shevchenko, I., et al., *Low-dose gemcitabine depletes regulatory T cells and improves survival in the orthotopic Panc02 model of pancreatic cancer.* Int J Cancer, 2013. **133**(1): p. 98-107.
22. de Biasi, A.R., J. Villena-Vargas, and P.S. Adusumilli, *Cisplatin-induced antitumor immunomodulation: a review of preclinical and clinical evidence.* Clin Cancer Res, 2014. **20**(21): p. 5384-91.
23. Disis, M.L., *Immune regulation of cancer.* J Clin Oncol, 2010. **28**(29): p. 4531-8.
24. Dong, H.D., et al., *Tumor-associated B7-H1 promotes T-cell apoptosis: A potential mechanism of immune evasion (vol 8, pg 793, 2002).* Nature Medicine, 2002. **8**(9).
25. Sharpe, A.H. and G.J. Freeman, *The B7-CD28 superfamily.* Nat Rev Immunol, 2002. **2**(2): p. 116-26.
26. Brown, J.A., et al., *Blockade of programmed death-1 ligands on dendritic cells enhances T cell activation and cytokine production.* J Immunol, 2003. **170**(3): p. 1257-66.
27. Francisco, L.M., P.T. Sage, and A.H. Sharpe, *The PD-1 pathway in tolerance and autoimmunity.* Immunol Rev, 2010. **236**: p. 219-42.
28. Thompson, R.H., et al., *PD-1 is expressed by tumor-infiltrating immune cells and is associated with poor outcome for patients with renal cell carcinoma.* Clin Cancer Res, 2007. **13**(6): p. 1757-61.
29. Talmadge, J.E., M. Donkor, and E. Scholar, *Inflammatory cell infiltration of tumors: Jekyll or Hyde.* Cancer Metastasis Rev, 2007. **26**(3-4): p. 373-400.
30. Usubutun, A., et al., *Prognostic factors in renal cell carcinoma.* J Exp Clin Cancer Res, 1998. **17**(1): p. 77-81.
31. Al-Shibli, K.I., et al., *Prognostic effect of epithelial and stromal lymphocyte infiltration in non-small cell lung cancer.* Clin Cancer Res, 2008. **14**(16): p. 5220-7.
32. Deschoolmeester, V., et al., *Tumor infiltrating lymphocytes: an intriguing player in the survival of colorectal cancer patients.* BMC Immunol, 2010. **11**: p. 19.
33. Diez, M., et al., *Histopathologic prognostic score in colorectal adenocarcinomas.* Anticancer Res, 1998. **18**(1B): p. 689-94.
34. Galon, J., et al., *Type, density, and location of immune cells within human colorectal tumors predict clinical outcome.* Science, 2006. **313**(5795): p. 1960-4.
35. Hiraoka, N., *Tumor-infiltrating lymphocytes and hepatocellular carcinoma: molecular biology.* Int J Clin Oncol, 2010. **15**(6): p. 544-51.

36. Nobili, C., et al., *Prolonged survival of a patient affected by pancreatic adenocarcinoma with massive lymphocyte and dendritic cell infiltration after interleukin-2 immunotherapy. Report of a case.* Tumori, 2008. **94**(3): p. 426-30.
37. Hodi, F.S. and G. Dranoff, *The biologic importance of tumor-infiltrating lymphocytes.* J Cutan Pathol, 2010. **37 Suppl 1**: p. 48-53.
38. Kloor, M., *Lymphocyte infiltration and prognosis in colorectal cancer.* Lancet Oncol, 2009. **10**(9): p. 840-1.
39. Hillen, F., et al., *Leukocyte infiltration and tumor cell plasticity are parameters of aggressiveness in primary cutaneous melanoma.* Cancer Immunol Immunother, 2008. **57**(1): p. 97-106.
40. Lee, H.E., et al., *Prognostic implications of type and density of tumour-infiltrating lymphocytes in gastric cancer.* Br J Cancer, 2008. **99**(10): p. 1704-11.
41. Leffers, N., et al., *Prognostic significance of tumor-infiltrating T-lymphocytes in primary and metastatic lesions of advanced stage ovarian cancer.* Cancer Immunol Immunother, 2009. **58**(3): p. 449-59.
42. Nishimura, H., T. Honjo, and N. Minato, *Facilitation of beta selection and modification of positive selection in the thymus of PD-1-deficient mice.* J Exp Med, 2000. **191**(5): p. 891-8.
43. Liotta, F., et al., *Frequency of regulatory T cells in peripheral blood and in tumour-infiltrating lymphocytes correlates with poor prognosis in renal cell carcinoma.* BJU Int, 2011. **107**(9): p. 1500-6.
44. Maude, S.L., et al., *Chimeric antigen receptor T cells for sustained remissions in leukemia.* N Engl J Med, 2014. **371**(16): p. 1507-17.
45. Kandoth, C., et al., *Mutational landscape and significance across 12 major cancer types.* Nature, 2013. **502**(7471): p. 333-9.
46. Herbst, R.S., et al., *A study of MPDL3280A, an engineered PD-L1 antibody in patients with locally advanced or metastatic tumors.* ASCO Meeting Abstracts, 2013. **31**(15_suppl): p. 3000.
47. Soria, J., et al., *Clinical activity, safety and biomarkers of PD-L1 blockade in non-small cell lung cancer (NSCLC): Additional analyses from a clinical study of the engineered antibody MPDL3280A (anti-PDL1).* European Journal of Cancer, 2013. **49**(Supplement 2).
48. Powles, T., et al., *MPDL3280A (anti-PD-L1) treatment leads to clinical activity in metastatic bladder cancer.* Nature, 2014. **515**(7528): p. 558-62.
49. Plimack, E.R., et al., *LBA23 - A phase 1b study of pembrolizumab (Pembro; MK-3475) in patients (Pts) with advanced urothelial tract cancer.* 2014.
50. Rosenberg, J.E., et al., *Atezolizumab in patients with locally advanced and metastatic urothelial carcinoma who have progressed following treatment with platinum-based chemotherapy: a single-arm, multicentre, phase 2 trial.* Lancet, 2016.
51. Balar, A.V., et al., *Atezolizumab as first-line treatment in cisplatin-ineligible patients with locally advanced and metastatic urothelial carcinoma: a single-arm, multicentre, phase 2 trial.* Lancet, 2017. **389**(10064): p. 67-76.
52. Sharma, P., et al., *Nivolumab in metastatic urothelial carcinoma after platinum therapy (CheckMate 275): a multicentre, single-arm, phase 2 trial.* Lancet Oncol, 2017.

53. Massard, C., et al., *Safety and Efficacy of Durvalumab (MEDI4736), an Anti-Programmed Cell Death Ligand-1 Immune Checkpoint Inhibitor, in Patients With Advanced Urothelial Bladder Cancer.* J Clin Oncol, 2016. **34**(26): p. 3119-25.
54. Bellmunt, J., et al., *Pembrolizumab as Second-Line Therapy for Advanced Urothelial Carcinoma.* N Engl J Med, 2017.
55. Merck, *Investigator's Brochure Pembrolizumab (MK-3475)*. 2014.
56. Zaid, H.B., et al., *Trends in the utilization of neoadjuvant chemotherapy in muscle-invasive bladder cancer: results from the National Cancer Database.* Urology, 2014. **83**(1): p. 75-80.
57. Splinter, T.A., et al., *The prognostic value of the pathological response to combination chemotherapy before cystectomy in patients with invasive bladder cancer. European Organization for Research on Treatment of Cancer--Genitourinary Group.* J Urol, 1992. **147**(3): p. 606-8.
58. Schultz, P.K., et al., *Neoadjuvant chemotherapy for invasive bladder cancer: prognostic factors for survival of patients treated with M-VAC with 5-year follow-up.* J Clin Oncol, 1994. **12**(7): p. 1394-401.
59. Epstein, J.I., et al., *The World Health Organization/International Society of Urological Pathology consensus classification of urothelial (transitional cell) neoplasms of the urinary bladder. Bladder Consensus Conference Committee.* Am J Surg Pathol, 1998. **22**(12): p. 1435-48.
60. Damrauer, J.S., et al., *Intrinsic subtypes of high-grade bladder cancer reflect the hallmarks of breast cancer biology.* Proc Natl Acad Sci U S A, 2014. **111**(8): p. 3110-5.
61. Siefker-Radtke, A., et al., *The basal subtype to predict clinical benefit from neoadjuvant chemotherapy: Final results from a phase II clinical trial of DDMVAC plus bevacizumab.* ASCO Meeting Abstracts, 2015. **33**(Suppl 6): p. Abstr 291.
62. Choi, W., et al., *Identification of distinct basal and luminal subtypes of muscle-invasive bladder cancer with different sensitivities to frontline chemotherapy.* Cancer Cell, 2014. **25**(2): p. 152-65.
63. Kardos, J., et al., *Claudin-low bladder tumors are immune infiltrated and actively immune suppressed.* JCI Insight, 2016. **1**(3): p. e85902.
64. Levey, A.S., et al., *A new equation to estimate glomerular filtration rate.* Ann Intern Med, 2009. **150**(9): p. 604-12.
65. Johnson, D.B., et al., *Fulminant Myocarditis with Combination Immune Checkpoint Blockade.* N Engl J Med, 2016. **375**(18): p. 1749-1755.
66. Simon, R., *Optimal two-stage designs for phase II clinical trials.* Control Clin Trials, 1989. **10**(1): p. 1-10.

11.0 APPENDICES

11.1 Appendix A: ECOG Performance Status

Grade	Description
0	Normal activity. Fully active, able to carry on all pre-disease performance without restriction.
1	Symptoms, but ambulatory. Restricted in physically strenuous activity, but ambulatory and able to carry out work of a light or sedentary nature (e.g., light housework, office work).
2	In bed <50% of the time. Capable of only limited self-care, confined to bed or chair more than 50% of waking hours.
3	In bed >50% of the time. Capable of only limited self-care, confined to bed or chair more than 50% of waking hours.
4	100% bedridden. Completely disabled. Cannot carry on any self-care. Totally confined to bed or chair.
5	Dead.
* As published in Am. J. Clin. Oncol.: *Oken, M.M., Creech, R.H., Tormey, D.C., Horton, J., Davis, T.E., McFadden, E.T., Carbone, P.P. Toxicity and Response Criteria of the Eastern Cooperative Oncology Group. Am J Clin Oncol* 5:649-655, 1982. The Eastern Cooperative Oncology Group, Robert Comis M.D., Group Chair.	

11.2 Appendix B: Patient-Reported Symptom Assessment

Subject ID:___________ Date:_______________

Please respond to each item by marking one box per row

**General Health**	**Excellent**	**Very Good**	**Good**	**Fair**	**Poor**
1. In general, would you say your health is...	☐	☐	☐	☐	☐

**Physical Function**	**Completely**	**Mostly**	**Moderately**	**A little**	**Not at all**
2. To what extent are you able to carry out your everyday physical activities such as walking, climbing stairs, carrying groceries, or moving a chair?	☐	☐	☐	☐	☐

NCI PRO-CTCAE™ ITEMS

Item Library Version 1.0

As individuals go through treatment for their cancer they sometimes experience different symptoms and side effects. For each question, please check or mark an ☒ in the one box that best describes your experiences over the past 7 days...

1.	In the last 7 days, how OFTEN did you have NAUSEA?				
	○ Never	○ Rarely	○ Occasionally	○ Frequently	○ Almost constantly
	In the last 7 days, what was the SEVERITY of your NAUSEA at its WORST?				
	○ None	○ Mild	○ Moderate	○ Severe	○ Very severe

2.	In the last 7 days, how OFTEN did you have LOOSE OR WATERY STOOLS (DIARRHEA)?					
	○ Never	○ Rarely	○ Occasionally	○ Frequently	○ Almost constantly	○ Not applicable

3.	In the last 7 days, what was the SEVERITY of your SHORTNESS OF BREATH at its WORST?				
	○ None	○ Mild	○ Moderate	○ Severe	○ Very severe
	In the last 7 days, how much did your SHORTNESS OF BREATH INTERFERE with your usual or daily activities?				
	○ Not at all	○ A little bit	○ Somewhat	○ Quite a bit	○ Very much

4.	In the last 7 days, what was the SEVERITY of your COUGH at its WORST?				
	○ None	○ Mild	○ Moderate	○ Severe	○ Very severe
	In the last 7 days, how much did COUGH INTERFERE with your usual or daily activities?				
	○ Not at all	○ A little bit	○ Somewhat	○ Quite a bit	○ Very much

5.	In the last 7 days, did you have any RASH?	
	○ Yes	○ No

6.	In the last 7 days, what was the SEVERITY of your ITCHY SKIN at its WORST?				
	○ None	○ Mild	○ Moderate	○ Severe	○ Very severe

The PRO-CTCAE™ items and information herein were developed by the NATIONAL CANCER INSTITUTE at the NATIONAL INSTITUTES OF HEALTH, in Bethesda, Maryland, U.S.A. Use of the PRO-CTCAE™ is subject to NCI's Terms of Use.

NCI PRO-CTCAE™ ITEMS

Item Library Version 1.0

7.	In the last 7 days, what was the SEVERITY of your NUMBNESS OR TINGLING IN YOUR HANDS OR FEET at its WORST?				
	○ None	○ Mild	○ Moderate	○ Severe	○ Very severe
	In the last 7 days, how much did NUMBNESS OR TINGLING IN YOUR HANDS OR FEET INTERFERE with your usual or daily activities?				
	○ Not at all	○ A little bit	○ Somewhat	○ Quite a bit	○ Very much

8.	In the last 7 days, what was the SEVERITY of RINGING IN YOUR EARS at its WORST?				
	○ None	○ Mild	○ Moderate	○ Severe	○ Very severe

9.	In the last 7 days, how OFTEN did you have PAIN?				
	○ Never	○ Rarely	○ Occasionally	○ Frequently	○ Almost constantly
	In the last 7 days, what was the SEVERITY of your PAIN at its WORST?				
	○ None	○ Mild	○ Moderate	○ Severe	○ Very severe
	In the last 7 days, how much did PAIN INTEREFERE with your usual or daily activities?				
	○ Not at all	○ A little bit	○ Somewhat	○ Quite a bit	○ Very much

10.	In the last 7 days, how OFTEN did you have ACHING MUSCLES?				
	○ Never	○ Rarely	○ Occasionally	○ Frequently	○ Almost constantly
	In the last 7 days, what was the SEVERITY of your ACHING MUSCLES at their WORST?				
	○ None	○ Mild	○ Moderate	○ Severe	○ Very severe
	In the last 7 days, how much did ACHING MUSCLES INTEREFERE with your usual or daily activities?				
	○ Not at all	○ A little bit	○ Somewhat	○ Quite a bit	○ Very much

The PRO-CTCAE™ items and information herein were developed by the NATIONAL CANCER INSTITUTE at the NATIONAL INSTITUTES OF HEALTH, in Bethesda, Maryland, U.S.A. Use of the PRO-CTCAE™ is subject to NCI's Terms of Use.

NCI PRO-CTCAE™ ITEMS

Item Library Version 1.0

11.	In the last 7 days, how OFTEN did you have ACHING JOINTS (SUCH AS ELBOWS, KNEES, SHOULDERS)?				
	○ Never	○ Rarely	○ Occasionally	○ Frequently	○ Almost constantly
	In the last 7 days, what was the SEVERITY of your ACHING JOINTS (SUCH AS ELBOWS, KNEES, SHOULDERS) at their WORST?				
	○ None	○ Mild	○ Moderate	○ Severe	○ Very severe
	In the last 7 days, how much did ACHING JOINTS (SUCH AS ELBOWS, KNEES, SHOULDERS) INTEREFERE with your usual or daily activities?				
	○ Not at all	○ A little bit	○ Somewhat	○ Quite a bit	○ Very much

12.	In the last 7 days, what was the SEVERITY of your FATIGUE, TIREDNESS, OR LACK OF ENERGY at its WORST?				
	○ None	○ Mild	○ Moderate	○ Severe	○ Very severe
	In the last 7 days, how much did FATIGUE, TIREDNESS, OR LACK OF ENERGY INTERFERE with your usual or daily activities?				
	○ Not at all	○ A little bit	○ Somewhat	○ Quite a bit	○ Very much

Do you have any other symptoms that you wish to report?	
○ Yes	○ No

Please list any other symptoms:

The PRO-CTCAE™ items and information herein were developed by the NATIONAL CANCER INSTITUTE at the NATIONAL INSTITUTES OF HEALTH, in Bethesda, Maryland, U.S.A. Use of the PRO-CTCAE™ is subject to NCI's Terms of Use.

NCI PRO-CTCAE™ ITEMS

Item Library Version 1.0

1.	In the last 7 days, what was the SEVERITY of this symptom at its WORST?				
	○ None	○ Mild	○ Moderate	○ Severe	○ Very severe
2.	In the last 7 days, what was the SEVERITY of this symptom at its WORST?				
	○ None	○ Mild	○ Moderate	○ Severe	○ Very severe
3.	In the last 7 days, what was the SEVERITY of this symptom at its WORST?				
	○ None	○ Mild	○ Moderate	○ Severe	○ Very severe
4.	In the last 7 days, what was the SEVERITY of this symptom at its WORST?				
	○ None	○ Mild	○ Moderate	○ Severe	○ Very severe
5.	In the last 7 days, what was the SEVERITY of this symptom at its WORST?				
	○ None	○ Mild	○ Moderate	○ Severe	○ Very severe

The PRO-CTCAE™ items and information herein were developed by the NATIONAL CANCER INSTITUTE at the NATIONAL INSTITUTES OF HEALTH, in Bethesda, Maryland, U.S.A. Use of the PRO-CTCAE™ is subject to NCI's Terms of Use.

11.3 Appendix C: The CKD-EPI Creatinine Equation

The CKD-EPI creatinine equation is based on the same four variables as the MDRD Study equation, but uses a 2-slope spline to model the relationship between estimated GFR and serum creatinine, and a different relationship for age, sex and race. The equation was reported to perform better and with less bias than the MDRD Study equation, especially in patients with higher GFR. This results in reduced misclassification of CKD. As of November 2009, very few clinical laboratories report the estimated GFR using the CKD-EPI creatinine equation. In the future, other GFR estimating equations may outperform CKD-EPI.

The CKD-EPI creatinine equation is:

$$\mathbf{GFR = 141 \times min(Scr/\kappa, 1)^{\alpha} \times max(Scr/\kappa, 1)^{-1.209}\ 0.993^{Age} \times 1.018\ [if\ female] \times 1.159\ [if\ black]}$$

κ = 0.7 if female

κ = 0.9 if male

α = -0.329 if female

α = -0.411 if male

min = the minimum of Scr/κ or 1

min = the maximum of Scr/κ or 1

Scr = serum creatinine (mg/dL)

Ref: Levey AS, Stevens LA, et al. A New Equation to Estimate Glomerular Filtration Rate. Ann Intern Med. 2009; 150:604-612.
