# Supplementary material for: Immune features are associated with response to neoadjuvant chemo-immunotherapy for muscle-invasive bladder cancer
Source: Nat Commun. 2024 May 24;15:4448. doi: 10.1038/s41467-024-48480-1 (PMC11126571; doi:10.1038/s41467-024-48480-1)
Supplement: Supplementary file 3 — Description of Additional Supplementary Files [file 41467_2024_48480_MOESM3_ESM.pdf]

Description of Additional Supplementary Files

Supplementary Data 1: Clinical data from LCCC1520 used to generate the manuscript figures. This file corresponds to Clinical1520.csv in Supplementary Code.

Supplementary Code 1: provides all code necessary to generate the figures and supplementary figures for the manuscript.
