# Supplementary material for: Immune features are associated with response to neoadjuvant chemo-immunotherapy for muscle-invasive bladder cancer
Source: Nat Commun. 2024 May 24;15:4448. doi: 10.1038/s41467-024-48480-1 (PMC11126571; doi:10.1038/s41467-024-48480-1)
Supplement: Supplementary file 6 — Reporting Summary [file 41467_2024_48480_MOESM6_ESM.pdf]

nature portfolio

Corresponding author(s): Jonathan S. Serody, William Y. Kim, Benjamin G. Vincent

Last updated by author(s): April 25, 2024

Reporting Summary

Nature Portfolio wishes to improve the reproducibility of the work that we publish. This form provides structure for consistency and transparency in reporting. For further information on Nature Portfolio policies, see our Editorial Policies and the Editorial Policy Checklist.

Statistics

For all statistical analyses, confirm that the following items are present in the figure legend, table legend, main text, or Methods section.

n/a	Confirmed	
☐	☒	The exact sample size (n) for each experimental group/condition, given as a discrete number and unit of measurement
☐	☒	A statement on whether measurements were taken from distinct samples or whether the same sample was measured repeatedly
☐	☒	The statistical test(s) used AND whether they are one- or two-sided<br>*Only common tests should be described solely by name; describe more complex techniques in the Methods section.*
☐	☒	A description of all covariates tested
☐	☒	A description of any assumptions or corrections, such as tests of normality and adjustment for multiple comparisons
☐	☒	A full description of the statistical parameters including central tendency (e.g. means) or other basic estimates (e.g. regression coefficient) AND variation (e.g. standard deviation) or associated estimates of uncertainty (e.g. confidence intervals)
☐	☒	For null hypothesis testing, the test statistic (e.g. F, t, r) with confidence intervals, effect sizes, degrees of freedom and P value noted<br>*Give P values as exact values whenever suitable.*
☐	☒	For Bayesian analysis, information on the choice of priors and Markov chain Monte Carlo settings
☐	☒	For hierarchical and complex designs, identification of the appropriate level for tests and full reporting of outcomes
☐	☒	Estimates of effect sizes (e.g. Cohen's d, Pearson's r), indicating how they were calculated

Our web collection on statistics for biologists contains articles on many of the points above.

Software and code

Policy information about availability of computer code

Data collection	LENS, FlowJo v8
Data analysis	R packages: ggplot2 v3.4.2, plyr v1.8.8, dplyr v1.1.2, maftools v2.16.0, flipPlots v1.3.6, ggpubr v0.6.0, pROC 1.18.2, ggcorrplot v0.1.4, gplots v3.1.3, scales v1.2.1, reshape2 v1.4.4, ggh4x v0.2.4, ggnewscale v0.4.9, glmnet v4.1.7, caret v6.0.94, caTools v1.18.2, survival v3.5.5, survminer v0.4.9, ggpmisc v0.5.5, matrixStats 1.0.0, ggrepel v0.9.3

For manuscripts utilizing custom algorithms or software that are central to the research but not yet described in published literature, software must be made available to editors and reviewers. We strongly encourage code deposition in a community repository (e.g. GitHub). See the Nature Portfolio guidelines for submitting code & software for further information.

Data

Policy information about availability of data

All manuscripts must include a data availability statement. This statement should provide the following information, where applicable:

- Accession codes, unique identifiers, or web links for publicly available datasets
- A description of any restrictions on data availability
- For clinical datasets or third party data, please ensure that the statement adheres to our policy

LCCC1520 trial:
The processed DNA and RNA sequencing data generated in this study have been deposited in the dbGaP database under accession code phs003452.v1.p1 [https://

www.ncbi.nlm.nih.gov/projects/gap/cgi-bin/study.cgi?study_id=phs003452.v1.p1]. The sequencing data are available under restricted access due to data privacy laws. Request access can be obtained through dbGAP. Access is permitted for one year.
Study protocol is available in the Supplementary information file. Individual de-identified patient clinical variables, besides patient age, are shared (Supplementary Data 1). Additional source data to reproduce main and supplementary figures are available in Figshare [https://doi.org/10.6084/m9.figshare.23705790].
Additional individual de-identified participant data, including age, can be shared upon request to the corresponding authors.

ABACUS trial:
The raw sequencing data are available under restricted access in the European Genome-Phenome Archive under accession EGAD00001006205 [https://ega-archive.org/datasets/EGAD00001006205]. Data access can be granted via the EGA.

The authors declare that all other data supporting the findings of this study are publicly available within the paper and its supplementary information files.

Research involving human participants, their data, or biological material

Policy information about studies with human participants or human data. See also policy information about sex, gender (identity/presentation), and sexual orientation and race, ethnicity and racism.

Reporting on sex and gender	We report on differential survival by gender. We did not assess biological sex.
Reporting on race, ethnicity, or other socially relevant groupings	We did not report on differences in outcome by race.
Population characteristics	Population characteristics were reported in the clinical trial summary in Rose et al. JCO 2021 as well as in our Supplementary Data.
Recruitment	*Describe how participants were recruited. Outline any potential self-selection bias or other biases that may be present and how these are likely to impact results.*
Ethics oversight	Our research was reviewed and approved by the institutional review boards at Duke University (Duke University Health System Institutional Review Board) and UNC (UNC Institutional Review Board) and conducted in accordance with the ethical criteria set by the Declaration of Helsinki. All participants provided written informed consent for the use of their tissue or peripheral blood for these correlative assessments.

Note that full information on the approval of the study protocol must also be provided in the manuscript.

Field-specific reporting

Please select the one below that is the best fit for your research. If you are not sure, read the appropriate sections before making your selection.

[x] Life sciences [ ] Behavioural & social sciences [ ] Ecological, evolutionary & environmental sciences

For a reference copy of the document with all sections, see nature.com/documents/nr-reporting-summary-flat.pdf

Life sciences study design

All studies must disclose on these points even when the disclosure is negative.

Sample size	The primary endpoint of this study is rate of pathological downstaging, defined as the proportion of patients who achieve absence of muscle-invasive disease at the time of cystectomy (<pT2). Based on a prior retrospective study [14] and two prospective randomized clinical trials (MRC/EORTC trial and Intergroup 0080 trial) utilizing a conventional schedule of cisplatin-based neoadjuvant chemotherapy, the null pathological downstaging rate is 35%.[5, 16]<br>A Simon's two-stage minimax design will be used for this study.[66]<br>The null<br>hypothesis that the true pathological downstaging rate is 35% will be tested against a one-sided alternative that it is higher. In the first stage, 21 patients will be accrued. If there are 8 or fewer patients with pathologic downstaging (<pT2) in these 21 patients, then the study will be stopped for futility, and the treatment regimen would be considered as uninteresting. Otherwise, 18 additional patients will be accrued for a total of 39. The null hypothesis will be rejected if 19 or more of the 39 patients have been downstaged to <pT2, and the treatment regimen will be considered worthy of future investigation. This design yields a type I error rate of 0.05 and power of 80% when the true response rate is 55%, representing a 20% improvement on the pathological downstaging rate previously reported.<br>(http://cancer.unc.edu/biostatistics/program/ivanova/SimonsTwoStageDesign.aspx)
Data exclusions	None

Replication	None; this will be the first study of neoadjuvant chemo-immunotherapy in muscle-invasive bladder cancer with public correlative data.
Randomization	Not applicable to study (single-arm trial)
Blinding	Not applicable to study (single-arm trial)

Reporting for specific materials, systems and methods

We require information from authors about some types of materials, experimental systems and methods used in many studies. Here, indicate whether each material, system or method listed is relevant to your study. If you are not sure if a list item applies to your research, read the appropriate section before selecting a response.

Materials & experimental systems

n/a	Involved in the study
☐	☒ Antibodies
☒	☐ Eukaryotic cell lines
☒	☐ Palaeontology and archaeology
☒	☐ Animals and other organisms
☐	☒ Clinical data
☒	☐ Dual use research of concern
☒	☐ Plants

Methods

n/a	Involved in the study
☒	☐ ChIP-seq
☐	☒ Flow cytometry
☒	☐ MRI-based neuroimaging

Antibodies

Antibodies used

T cell panel
Live/Dead (dye) FVS 700 BD 564997 N/A 1:8000
CD45RA BV711 BioLegend 304138 HI100 5uL
CD4 APC-H7 BD 560837 L200 5uL
CD8 PerCP-Cy5.5 BD 565310 SK1 5uL
CD25 PE BD 557138 M-A251 20uL
CD127 PE-Cy7 BD 560822 HIL-7R-M21 5uL
TIGIT BV421 BioLegend 372710 A15153G 5uL
PD1 BV510 BD 563076 EH12.1 5uL
CD39 BV650 BD 563681 TU66 5uL
CTLA4 BV605 BioLegend 369610 BNI3 5uL
Foxp3 AlexaFluor 647 BD 560045 259D/C7 20uL

B cell panel
Live/Dead (dye) FVS 700 BD 564997 N/A 1:8000
CD3 APC-Cy7 BioLegend 344818 SK7 5uL
CD14 APC-Cy7 BioLegend 325620 HCD14 5uL
CD16 APC-Cy7 BioLegend 302018 3G8 5uL
CD10 BV650 BD 563734 HI10a 5uL
CD19 BV785 BioLegend 302240 HIB19 5uL
CD38 APC BioLegend 303510 HIT2 5uL
CD21 BV711 BD 563163 B-ly4 5uL
CD27 PE-CF594 BD 562297 M-T271 5uL
IL21R BV421 BioLegend 347809 2G1-K12 5uL
IgD FITC BD 555778 IA6-2 20uL
Ki67 PerCP-Cy5.5 eBioscience 46-5699-42 20Raj1 5uL

Myeloid panel
Live/Dead (dye) FVS 700 BD 564997 N/A 1:8000
CD3 APC BD555342 HIT3a 20uL
CD19 APC BD555415 HIB19 20uL
CD56 APC BD555518 B159 20uL
CD33 BV711 BD 563171 WM53 5uL
CD11b APC-Cy7 BD 557754 ICRF44 5uL
CD11c PE-Cy7 BD 561356 B-ly6 5uL
CD14 BV605 BD 564054 M5E2 5uL
CD15 PerCP-Cy5.5 BD 560828 HI98 5uL
CD16 PE BD 556619 3G8 20uL
CD86 BV421 BD 562433 2331 5uL
CD123 BV510 BD 563072 9F5 5uL

	HLA-DR BB515 BD 564516 G46-6 5uL
Validation	All antibodies were validated by manufacturer in human cell lines.

Clinical data

Policy information about clinical studies

All manuscripts should comply with the ICMJEguidelines for publication of clinical research and a completedCONSORT checklist must be included with all submissions.

Clinical trial registration	NCT01690558
Study protocol	https://www.clinicaltrials.gov/study/NCT02690558
Data collection	Data collection was performed according to the methods described in Rose et al. JCO 2021.
Outcomes	Outcomes were defined in Rose et al. JCO 2021.

Plants

Seed stocks	*Report on the source of all seed stocks or other plant material used. If applicable, state the seed stock centre and catalogue number. If plant specimens were collected from the field, describe the collection location, date and sampling procedures.*
Novel plant genotypes	*Describe the methods by which all novel plant genotypes were produced. This includes those generated by transgenic approaches, gene editing, chemical/radiation-based mutagenesis and hybridization. For transgenic lines, describe the transformation method, the number of independent lines analyzed and the generation upon which experiments were performed. For gene-edited lines, describe the editor used, the endogenous sequence targeted for editing, the targeting guide RNA sequence (if applicable) and how the editor was applied.*
Authentication	*Describe any authentication procedures for each seed stock used or novel genotype generated. Describe any experiments used to assess the effect of a mutation and, where applicable, how potential secondary effects (e.g. second site T-DNA insertions, mosiacism, off-target gene editing) were examined.*

Flow Cytometry

Plots

Confirm that:

- [x] The axis labels state the marker and fluorochrome used (e.g. CD4-FITC).
- [x] The axis scales are clearly visible. Include numbers along axes only for bottom left plot of group (a 'group' is an analysis of identical markers).
- [x] All plots are contour plots with outliers or pseudocolor plots.
- [x] A numerical value for number of cells or percentage (with statistics) is provided.

Methodology

Sample preparation	PBMC immunophenotyping was designed, performed, and analyzed by Immune Monitoring and Genomics Facility, UNC Lineberger Comprehensive Cancer Center. Cryopreserved PBMCs were thawed in Dextran-Albumin (CSL Behring #44206-251-10) solution and washed with AIM-V CTS (Gibco #0879122DK)/5% of Human AB Serum (Gemini#100-512), following cell resuspension in 1X HBSS (Gibco #14175-095). Viable PBMC numbers were determined and distributed at 2 million cells per patient per timepoint per assay tube. Flow cytometry panels were used to analyze T cell (Supplementary Table 1), B cell (Supplementary Table 2), and myeloid (Supplementary Table 3) subsets. Samples were aliquoted and analyses performed separately for T, B, and myeloid cells. Intracellular staining for Foxp3 in T cell panel and Ki67 in B cell panel were performed by utilizing eBioscienceFoxp3/TF staining kit (ThermoFisher #00-5523-00).
Instrument	BD LSRFortessa (Serial# H64717700116)
Software	FlowJo v8
Cell population abundance	Cell population abundance calculation is described in Methods.
Gating strategy	The gating strategies are available in Supplementary Material.

- [x] Tick this box to confirm that a figure exemplifying the gating strategy is provided in the Supplementary Information.
